# Mechanochemical Approach towards Multi-Functionalized 1,2,3-Triazoles and Anti-Seizure Drug Rufinamide Analogs Using Copper Beads

**DOI:** 10.3390/molecules27227784

**Published:** 2022-11-11

**Authors:** Dhananjay Bhattacherjee, Igor S. Kovalev, Dmitry S. Kopchuk, Matiur Rahman, Sougata Santra, Grigory V. Zyryanov, Pralay Das, Rituraj Purohit, Vladimir L. Rusinov, Oleg N. Chupakhin

**Affiliations:** 1 Department of Organic and Biomolecular Chemistry, Ural Federal University, 19 Mira Street, 620002 Yekaterinburg, Russia; 2 I. Ya. Postovsky Institute of Organic Synthesis, Ural Branch of the Russian Academy of Sciences, 22 S. Kovalevskoi Street, 620219 Yekaterinburg, Russia; 3 Chemical Technology Division, CSIR-Institute of Himalayan Bioresource Technology (CSIR-IHBT), Palampur 176061, India; 4 Academy of Scientific and Innovative Research (AcSIR), Ghaziabad 201002, India; 5 Structural Bioinformatics Lab, CSIR-Institute of Himalayan Bioresource Technology (CSIR-IHBT), Palampur 176061, India

**Keywords:** click chemistry, mechanochemical synthesis, 1,2,3-triazole, cycloaddition reaction, Rufinamide synthesis, solvent-free

## Abstract

Highly regiospecific, copper-salt-free and neat conditions have been demonstrated for the 1,3-dipolar azide-alkyne cycloaddition (AAC) reactions under mechanochemical conditions. A group of structurally challenging alkynes and heterocyclic derivatives was efficiently implemented to achieve highly functionalized 1,4-disubstituted-1,2,3-triazoles in good to excellent yield by using the Cu beads without generation of unwanted byproducts. Furthermore, the high-speed ball milling (HSBM) strategy has also been extended to the synthesis of the commercially available pharmaceutical agent, Rufinamide, an antiepileptic drug (AED) and its analogues. The same strategy was also applied for the synthesis of the Cl-derivative of Rufinamide. Analysis of the single crystal XRD data of the triazole was also performed for the final structural confirmation. The Cu beads are easily recoverable from the reaction mixture and used for the further reactions without any special treatment.

## 1. Introduction

**Scheme 1:**
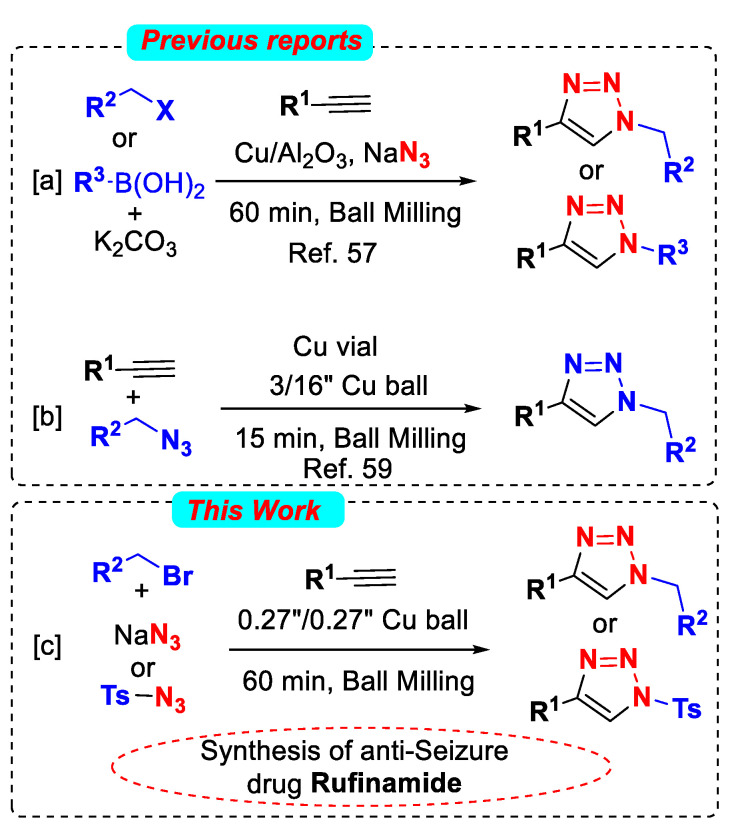
Mechanochemical strategies of CuAAC reaction. (**a**) Previous approach of AAC reaction using Cu/Al_2_O_3_ as catalyst under ball milling; (**b**) Previous approach using Cu-vial and 3/16” Cu ball under ball milling; (**c**) The present approach under ball milling conditions.

The 1,2,3-triazole moiety represents one of the versatile classes of heterocycles because of their widespread applications as pharmaceutical agents, agrochemicals, biochemicals and polymers [1,2,3,4,5,6,7,8,9,10,11,12,13,14]. The seminal work on “click chemistry” by Huisgen, followed by the further independent development by Meldal et al. and Sharpless-Fokin has encouraged extensive research on the 1,2,3-triazole molecule [15,16,17]. Over the last two decades, a plethora of reports have been documented for the 1,3-dipolar azide-alkyne cycloaddition (AAC) reaction and mostly involves Cu catalysis [18,19,20,21,22,23,24,25,26,27]. The assessment transition metal catalysis for the synthesis of heterocycles is common practice in modern research. Hence, several other transition metals, such as Pd, Ru, Zn, Ag, Ni, Au, etc., have also been efficiently manifested for the AAC reaction [28,29,30,31,32,33,34,35,36,37,38,39,40,41,42,43]. Among the 12 principles of green chemistry, the use of non-toxic and/or volatile organic solvents, minimal generation of organic wastes, atom economic synthesis and the use of environmentally benign chemicals have introduced an upsurging interest in contemporary organic synthesis. This field is also emerging with the use of various nonconventional energy sources, such as microwave, ultrasound, mechanical mixing, electrochemical methods and visible-light-driven organic transformations [44]. Complementing solution-based synthesis, the mechanochemical operations provide an environmentally benign alternative to negate the demand for bulk organic solvents, thereby finding an application in a plethora of organic transformations [45,46,47,48,49,50,51]. In a broader sense, the application of mechanical energies such as compression, shearing or friction under solvent-free conditions have been promising techniques for the utilization of mechanoresponsive materials to access active pharmaceutical ingredients (API) and thereby making a strong impact for pharmaceutical industries [52]. This technique also provides a cleaner source of energy for organic transformations. In 2011, the planetary ball mill was used by Schubert et al. for a solvent-free AAC reaction using catalytic amounts of Cu(OAc)_2_ and sodium ascorbate [53]. Later on, several reports for the solvent-free synthesis of 1,2,3-triazoles were completed in which a homogeneous Cu catalyst or stoichiometric amount of Cu powder were used [54,55,56]. To enhance the catalyst regeneration in these reactions, the immobilization of copper on the heterogenous matrix has been employed. In 2013, Ranu et al. efficiently demonstrated Cu/Al_2_O_3_ as catalyst for the AAC reaction under ball milling without using any solvent and additive [57] (Scheme 1a). Recently, Amini et al. showed the catalytic activity of immobilised Cu NPs on WO3 surface for the AAC reaction under solvent-free conditions [58]. For the first time, the Mack laboratory introduced the use of Cu-vial and 3/16” Cu ball in an efficient AAC reaction under 16 h milling in solvent-free conditions [59] (Scheme 1b).

In their strategy, three component reactions such as phenylacetylene, benzyl bromide and sodium azide, on grinding for 16h, afforded the desired 1-benzyl-4-phenyltriazole in quantitative yield under the one-step, one-vial multicomponent CuAAC reaction.

Epilepsy is a chronic neurological disorder in the brain that affects people of all ages worldwide. Rufinamide (brand name Banzel [60] or Inovelon [61], developed by Novartis and manufactured by Eisai) has been already reported as a sodium channel blocker and an antiepileptic drug (AED) with a broad spectrum of efficacy. It is an FDA-approved orphan drug used for the adjunctive treatment of seizures associated with Lennox–Gastaut syndrome (LGS). The common strategies for the synthesis of Rufinamide involve 1,3-dipolar cycloaddition reaction using 2-chloroacrylonitrile, propiolic acid and esters or (E)-methyl 3-methoxyacrylate [62,63,64,65,66,67]. Recent synthetic developments involve flow microreactor systems via multistep synthesis in a compartmentalized continuous flow integrated with in-line separation techniques [68,69,70,71]. We envisioned that mechanochemical conditions could be useful for the synthesis of Rufinamide and its analogs via [2+3] CuAAC reaction and, to the best of our knowledge, this approach is still uncommon in the literature. Herein we wish to report a mechanochemical strategy of 1,3-dipolar Huisgen cycloaddition of various azides, generated in situ, with a dipolarophile (alkyne) to construct structurally important 1,2,3-triazoles as well as Rufinamide and its analogs by using Cu beads (Scheme 1c).

## 2. Results and Discussion

**Figure 1:**
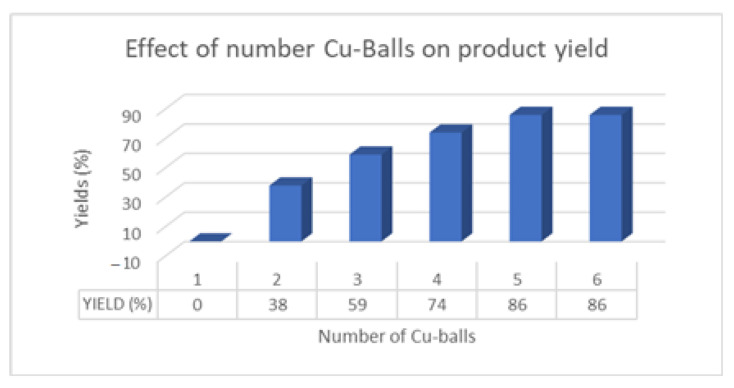
Effect of cylindrical Cu beads (0.27/0.27″) on the product yield.

At the commencement of our investigation, we chose 4-ethylnyl toluene (**1a**) and 2-bromo-1-(*m*-tolyl)ethan-1-one (**2a**) as bench stable substrates to react in the presence of inorganic azide. Different mechanochemical parameters of the reaction such as time, rpm limit, equivalency, and number of copper balls were optimized in order to obtain the desired 1,2,3-triazoles in the highest yield. The results of the optimization of the mechanochemical reaction conditions are reported below (Table 1). Thus, we have observed that **1a** (50 mg, 0.431 mmol), **2a** (183.6 mg, 0.862 mmol) and NaN_3_ (56 mg, 0.862 mmol) satisfactorily afforded **3a** in 86% of yield under neat reaction conditions for 3h of mechanochemical grinding with 5-Cu beads at 500 rpm (Table 1, Entry 4). Intriguingly, the overall yield of the triazoles also depends on the number of Cu beads in the reaction (Figure 1).

**Figure 2:**
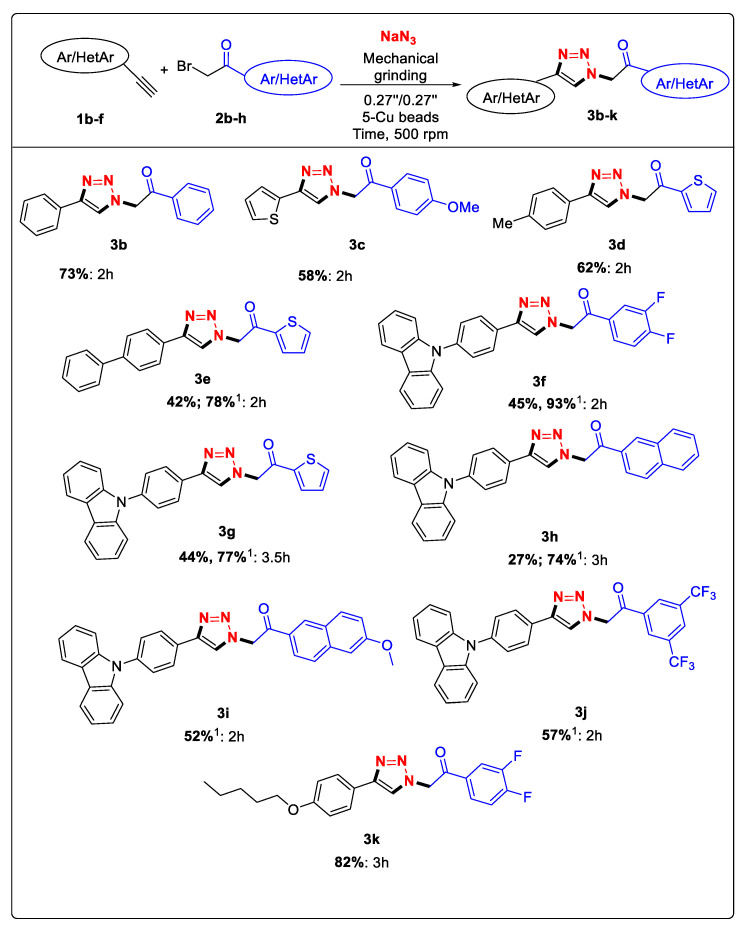
Scope of various alkynes and benzoylmethyl bromides in mechanochemical CuAAC reaction. Reaction conditions: Ar/HetAr alkynes **1b**–**f** (1 equiv.), benzoylmethyl bromides **2b**–**h** (1.2 equiv.), sodium azide (1.2 equiv.), 5-Cu beads, 500 rpm; ^1^ stepwise route.

**Figure 3:**
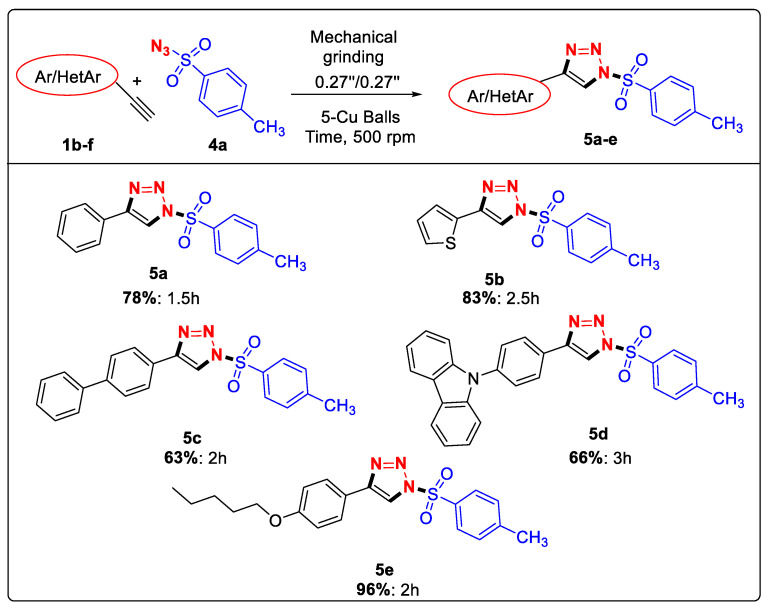
Scope of various alkynes in CuAAC with tosyl azides under mechanochemical conditions. Reaction conditions: Ar/HetAr alkynes **1b**–**f** (1 equiv.), tosyl azide **4a** (1.2 equiv.), 5-Cu beads, 500 rpm.

These conditions were useful for the further assessment of the various alkynes and benzoylmethyl bromides. We did not observe much electronic control of different alkynes as well as benzoylmethyl bromides over the reaction yield for the three component reactions, and products **3b**–**d** were obtained in 63–73% yield. In all the cases we isolated unreacted starting alkynes in small quantities. In the case of biphenyl acetylene, the observed yield of the product **3e** was 42% and the conversion of the starting material was poor. The poor yield may be attributed to the three-component reaction in the presence of unactivated copper beads, as well as the low reactivity of sterically and electronically unfavorable alkyne species. Changing the equivalency or the grinding time did not improve the yield of the desired product significantly. To our delight, an excellent yield of **3e** was found by changing the reaction technique. The same reaction was carried out in a stepwise fashion, in which the benzoylmethyl bromide derivatives were first converted into corresponding azido derivatives (see Supplementary Materials) and then employed under optimized mechanochemical conditions. Then, we introduced structurally interesting and highly sterically hindered alkynes for the anticipated CuAAC reaction. Under the conditions of three-component coupling, we again encountered low-to-moderate yield for the compounds **3f**–**k** (Figure 2). However, the stepwise fashion of the mechanochemical reaction gave excellent yield of the products **3f**–**k** (Figure 2). In all these reactions, benzoylmethyl bromide derivatives had no marked effect on the yield of the final 1,2,3-triazole derivatives (Figure 2). It is noteworthy that the mechanochemical synthesis of 1,2,3-triazole lead to the formation of only 1,4- regioisomers and formation of 1,2-isomers were not observed. Owing to the inherent biological activity of the sulphonamides, we have targeted triazole based sulphonamides molecules under mechanochemical conditions (Figure 3). We encountered the low yield of products in the case of three-component mechanochemical coupling of alkynes, sodium azides and tosylchlorides. Therefore, the tosylazides (**4a**) were prepared using the reported conditions (see Supplementary Materials) and then employed in the 1,3-dipolar cycloaddition. In all cases, good-to-excellent yield of the triazole-based sulphonamides **5a**–**e** were observed. Interestingly, only the formation of 1,4-regioisomer was observed to have excellent selectivity.

**Figure 4:**
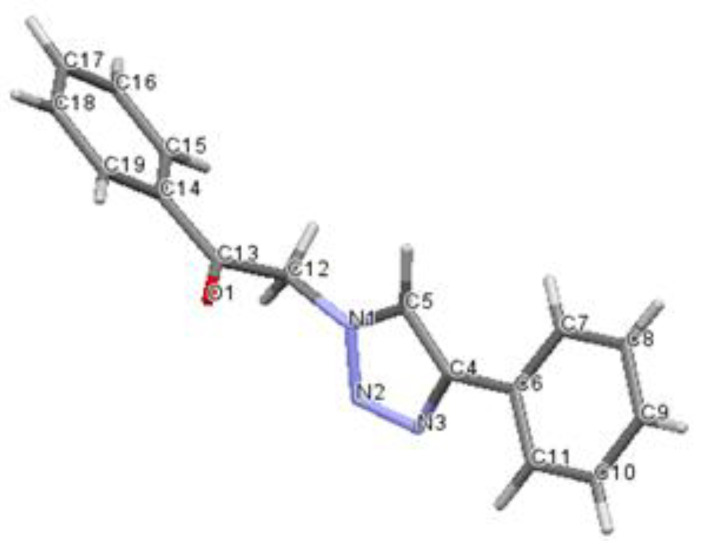
Single crystal XRD structure of **3b**. CCDC number for the compound **3b** is 2123952.

Finally, to confirm the structure of the obtained products, single-crystal XRD experiments were carried out for the 1,2,3-triazole **3b,** and the obtained single-crystal XRD structure is presented below (Figure 4).

**Scheme 2:**
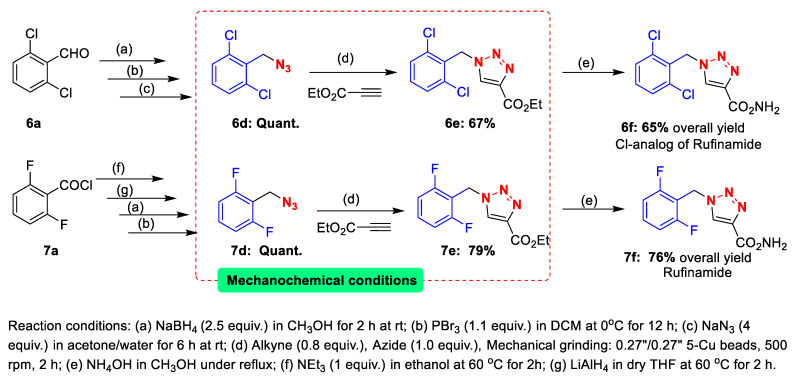
Total synthesis of anti-seizure drug Rufinamide and analogues.

To demonstrate the synthetic utility of the present reaction, we successfully prepared Rufinamide (compound **7f**), a commercially available antiepileptic drug (AED) and its Cl-analogue (compound **6f**) in good overall yields (Scheme 2). The Cl-analogue of Rufinamide was prepared by starting from easily available 2,6-dichloro benzaldehyde precursor followed by the synthesis of 2,6-dichlorobenzyl azides (see Supplementary Materials). Without any tedious purification of these organic azides, we treated with propiolic esters under optimised mechanochemical grinding using copper beads. The ethyl ester of the propiolic acid under our optimised mechanochemical conditions gave the formation of the desired 1,4-isomer only compared to the methyl ester derivative of propiolic acid derivatives. To our delight, we observed the formation of yellow crude after the reaction which contained only 1,4-regioisomer (**6e**) as a major product and gave 67% yield after purification. We have also observed that the mechanochemical conditions gave better results in the case of two-component reactions, i.e., alkyne and organic azide, rather than three. The triazlic esters (**6e**) can be easily converted into the amide derivatives using treatment with ammonia water in methanol. A similar experimental procedure was followed for the synthesis of the commercially available drug Rufinamide **7f** in 79% of overall yield with greater selectivity (Scheme 2).

### Plausible Catalytic Pathway

**Figure 5:**
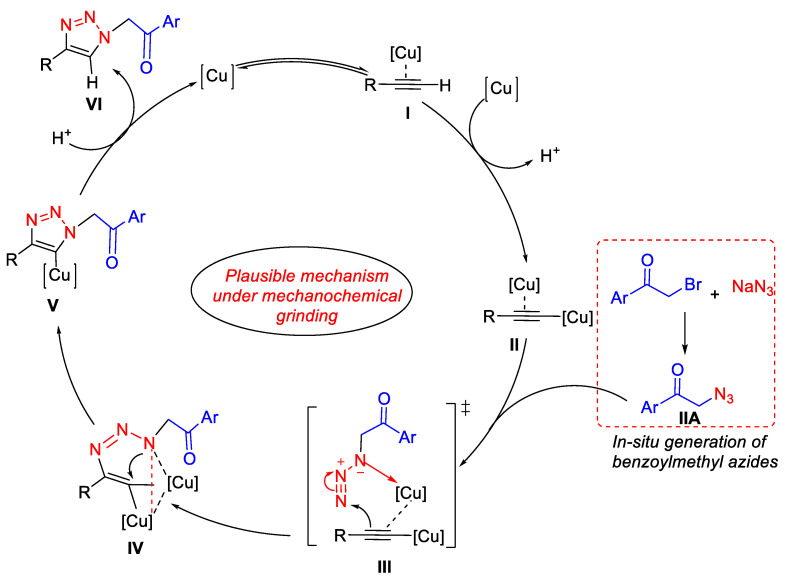
Plausible reaction pathway of the Huisgen cycloaddtion.

From the previous discussions, we have observed the in situ generation of stable and isolable organic azides (**IIA**) as the key intermediate, followed by 1,2,3-triazole formation. In some cases, we also performed reactions between the alkynes and organic azides to enhance the overall yield. Based on the previous literature reports [72,73,74,75,76] and the above experimental findings, a plausible reaction mechanism is suggested as shown in Figure 5. The proposed catalytic cycle for the CuAAC of alkynes with the azides consists of an initial copper acetylide formation to afford intermediate **I**. We surmised that, in the catalytic cycle, NaN_3_ can change the valence state of Cu during the reaction and that this might be responsible for the observed activity [77,78]. The Cu(I) species reacts with an alkyne to create a copper acetylide. On the other hand, benzoylmethyl bromides react with sodium azides to form benzoylmethyl azides, **IIA**, which are one of the key intermediates in the catalytic cycle. The 1,3-dipolar cyclization of the resulting dinuclear copper intermediate (**III** and **IV**) and benzoylmethyl azides **IIA**, followed by protonation, provided the formation of target 1,2,3-triazole **VI** and the regeneration of the Cu catalyst. The generation of intermediate **III** and **IV** is supported by reference [73]. It worth mentioning that Cu-beads are recyclable, and after sonication with acetone the Cu-beads can be returned to the reaction without losing both the grinding performance and the catalytic activity.

## 3. Conclusions

In summary, we have developed a regiospecific, environmentally benign mechanochemical grinding for a 1,3-dipolar Huisgen cycloaddition reaction between terminal alkynes and azides using Cu-beads. Highly functionalized 1,2,3-triazoles were prepared selectively in good-to-excellent yield using an easy workup technique and without generation of unwanted waste. This energy- and cost-effective process has also been extended for the synthesis of Rufinamide, a commercially available antiepileptic drug (AED) and its Cl-analog. The crystallographic data of the triazole molecule also established structural confirmation. Furthermore, the in silico studies of the prepared molecules are still under investigation and the results will be published in due course. Finally, this research may encourage the synthetic community to develop active pharmaceutical ingredients using greener energy sources and impact the pharmaceutical industries.

## 4. Experimental Section

General experimental procedure for the mechanochemical cycloaddition reaction: PM100 stainless steel grinding bowl with an internal volume of 25 mL, containing 0.27/0.27-inch cylindrical copper beads (5 beads) was charged with alkynes (1 equivalent), equimolar quantities of benzoylmethylbromide (2 equiv. unless otherwise mentioned) and sodium azide (2 equiv. otherwise mentioned). The grinding bowl was then equipped with a stainless steel bowl cap and placed in the mechanical ball milling instrument. The reaction mixture within the grinding bowl was allowed to rotate for 3 h (unless otherwise mentioned) at the speed of 500 rpm. The progress of the reaction was monitored by the TLC and the reaction mixture was extracted with dichloromethane. The crude was concentrated under reduced pressure and the product was isolated using silica gel (230–400) column chromatography under hexane/ethylacetate gradient.

## Figures and Tables

**Table 1:**
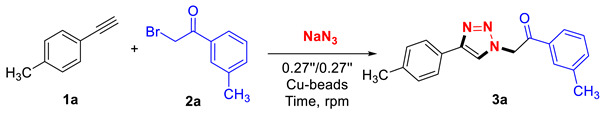
Mechanochemical optimization of reaction parameters ^1^.

Entry	Equiv. of Azide	RPM	Time (h)	Conversion (%)	Selectivity (%)	Yields (%) ^2^
1	1.2	300	1	68	35	24
2	1.2	400	2	80	70	56
3	1.2	500	3	88	89	79
4	2.0	500	3	98	88	86
5	2.0	500	4	98	88	86
6	2.0	400	3	82	78	64
7	2.4	500	3	98	88	86

^1^ Reaction conditions: **1a** (0.431 mmol), **2a** (0.862 mmol), sodium azide (0.862 mmol), 5-Cu beads, 500 rpm; ^2^ yields refer after chromatographic purification.

## Data Availability

Data are contained within the article or Supplementary Material.

## Supplementary Materials

The following are available online at https://www.mdpi.com/article/10.3390/molecules27227784/s1, Figure S1: GC-MS of azidation of tosylchloride, Figure S2: Synthesis of 2,6-dichlorobenzyl azide from its aldehyde precursor, Figure S3: Reduction of 2,6-difluorobenzoyl chloride to 2,6-difluorobenzyl azide, Figure S4: Spectral data (^1^H, ^13^C, GC-MS) for synthesized compounds, Figure S5: ORTEP diagram of compound **3b [79]**.
