# Supplementary material for: Mechanochemical Approach towards Multi-Functionalized 1,2,3-Triazoles and Anti-Seizure Drug Rufinamide Analogs Using Copper Beads
Source: Molecules. 2022 Nov 11;27(22):7784. doi: 10.3390/molecules27227784 (PMC9693609; doi:10.3390/molecules27227784)
Supplement: Supplementary file 1 [file molecules-27-07784-s001.zip › molecules-1989444-supplementary.pdf]

Mechanochemical Approach towards Multi-Functionalized 1,2,3-Triazoles and Anti-Seizure Drug Rufinamide Analogs Using Copper Beads

Dhananjay Bhattacherjee,*[a] Igor S. Kovalev,[a] Dmitry. S. Kopchuk,[a,b] Matiur Rahman,[a] Sougata Santra,*[a] Grigory V. Zyryanov,[a,b] Pralay Das,[c,d] Rituraj Purohit,[d,e] Vladimir L. Rusinov,[a,b] Oleg N. Chupakhin[a,b]

[a] Ural Federal University named after the first President of Russia B. N. Yeltsin, 19 ul. Mira, 620002 Ekaterinburg, Russian Federation; bhattacherjee130@gmail.com

[b] I. Ya. Postovsky Institute of Organic Synthesis, Ural Branch of the Russian Academy of Sciences, 22 ul. S. Kovalevskoi, 620219 Ekaterinburg, Russian Federation
[for corresponding author(s) please include phone and fax number(s) and e-mail address(es)]

[c] Chemical Technology Division, CSIR-Institute of Himalayan Bioresource Technology (CSIR-IHBT), Palampur, 176061, H.P., India

[d] *Academy of Scientific and Innovative Research (AcSIR), Ghaziabad, 201002, India*

[e] *Structural Bioinformatics Lab, CSIR-Institute of Himalayan Bioresource Technology (CSIR-IHBT), Palampur, 176061, H.P., India*

Table of Content

Section	Page
A. General Information	S2
B. Experimental Procedures	S2-S5
C. Synthesis and characterization data for the products triazoles (3a-i, 5a-d, 6e and 7e)	S5-S12
D. Spectral data (^{1}H, ^{13}C NMR, GC-MS) for synthesized compounds (3a-i, 5a-d, 6e and 7e)	S12-S40
E. Single Crystal XRD data for the compound **3b**	S41
F. References	S42

(A) General Information:

Materials and Methods: All the chemicals and solvents were purchased from the commercial sources and used without any further purification unless otherwise synthesized in the laboratory. The Retsch PM100 ball milling instrument equipped with PM100 stainless steel grinding bowl were used having volume is 25 mL for all the mechanochemical operations. The cylindrical copper beads were used at the dimension of 0.27″(height)X0.27″(diameter). The synthesized compounds were identified by the mass spectra and NMR data before implementation in the reaction. The solvents were distilled or purified using standard procedures prior to its use. All the column chromatography has been caried out using silica-gel (230-450 mesh) as stationary phase and hexane/ethylacetate as mobile phase. Thin layer chromatography was performed using precoated silica gel on aluminum foils F_{254} (Merck) in UV light detector. Mass spectra were recorded on a Shimadzu GCMS-QP2010 Ultra (Japan) and the ionization method was electron ionization (EI). ^{1}H, ^{13}C NMR spectra were recorded using a Bruker Advance 400 spectrometer operating at 400 MHz for ^{1}H, 100 MHz ^{13}C. Spectra were recorded at 25 °C in $CDCl_3$ [residual $CHCl_3$ (δ_H 7.26 ppm) or $CDCl_3$ (δ_C 77.00 ppm) and DMSO-d6 [δ_H 2.5, 3.35 ppm; δ_C 39.52 ppm as international standard] with TMS as internal standard. Chemical shifts were recorded in δ (ppm) relative to the TMS and $CDCl_3$ signal, coupling constants (J) are given in Hz and multiplicities of signals are reported as follows: s, singlet; d, doublet; t, triplet; m, multiplet; brs, broad singlet; qt, quartet. The melting point of the solids were recorded by using Biocote, SMP10 melting point apparatus. X-ray crystallography data for the compound **3b** were recorded by using single crystal X-ray automated Xcalibur 3 diffractometer following standard procedure (MoK radiation, graphite monochromator, ω-scanning with a step of 1°).

(B) Experimental procedures:

B1) General experimental procedure for the mechanochemical cycloaddition reaction: PM100 stainless steel grinding bowl having internal volume 25 mL containing 0.27/0.27inch cylindrical copper beads (5 beads) was charged with Alkynes (1 equivalent) and equimolar quantities of benzoylmethylbromide (2 equivalent otherwise mentioned) and sodium azide (2 equivalent otherwise mentioned).The grinding bowl was then equipped with stainless steel bowl cap and placed in the mechanical ball milling instrument. The reaction mixture within the grinding bowl allowed to vibrated for 3 hours (otherwise mentioned) at the speed of 500 rpm. The progress of the reaction was monitored by the TLC and the reaction mixture was extracted

with dichloromethane. The crude was concentrated under reduced pressure and the product was isolated using silica gel(230-400) column chromatography under hexane/ethylacetate gradient.

B2) Typical experimental procedure for the azidation of tosylchloride: Preparation of tosyl azides were followed as per reported literature.[1] To a mechanically stirred solution of sodium azide (1.9g) in water and acetone (1:2) was rapidly added to a solution of p-toluenesulpphonyl chloride (5.0g). The mixture warmed slightly, and the colour became darkened and the two phases were formed. After stirring at ambient temperature for 2hrs, acetone was removed under reduced pressure and the dichloromethane was added to it. The organic phase was washed with water and dried over anhydrous Na_2SO_4 and concentrated under reduced pressure. Colourless liquid oil which on cooling rapidly crystallises to white solid. Mpt: 21-23 ^oC. The TLC shows only one spot under 254nm UV light. The GC-MS were performed which shows (m/z): 197 [M].

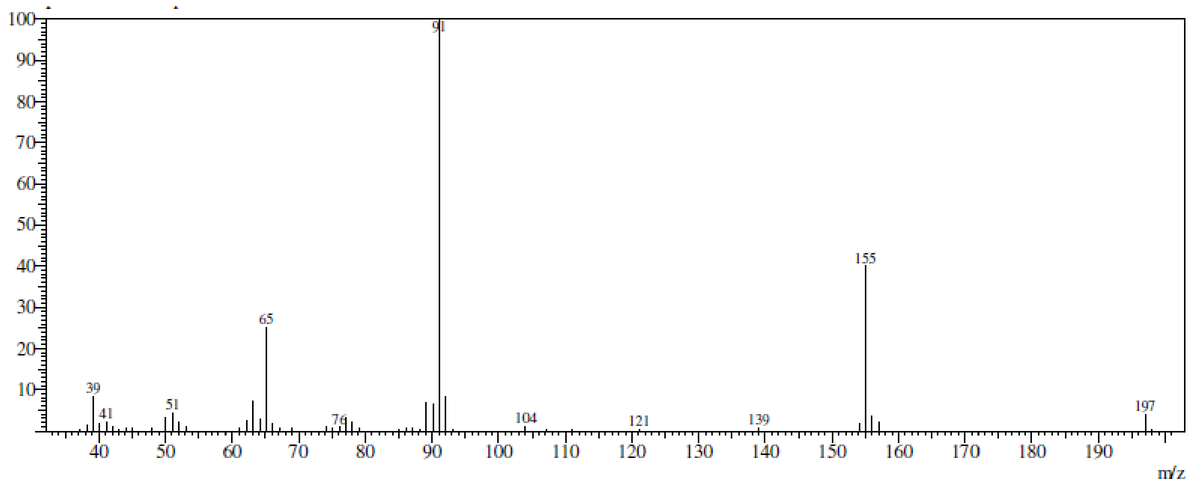

Figure S1: GC-MS of azidation of tosylchloride.

B3) General procedure for the synthesis of benzoylmethane azides derivatives: To a stirred solution of sodium azide (1.1 equiv.) in acetonitrile (5 mL) was added benzoylmethyl bromides (1.0 equiv.) at room temperature. After 8h of vigorous stirring, the reaction mixture was quenched by ice-water and extracted with dichloromethane (3x10 mL). The combined organic phases were dried (Na_2SO_4), filtered and evaporated in vacuo. The product was obtained quantitatively.

B4) Typical experimental procedure for synthesis of 2,6-dichlorobenzyl azide from its aldehyde precursor:

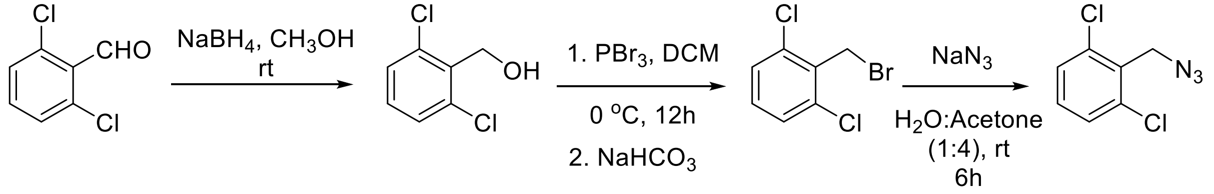

Figure S2: Synthesis of 2,6-dichlorobenzyl azide from its aldehyde precursor.

The 2,6-dichlorobenzylazides were obtained from its aldehyde derivatives in three steps**.** In the first step, a solution of 2,6-dichlorobenzaldehyde (1g) in CH_3OH was charged with sodium borohydride (540mg) portion wise for 10 mins. After 2h of continuous stirring at room temperature the solvent was evaporated, and the mass was dissolve in water (10 mL). The water solution was acidified with 3N HCl solution until the pH of the solution becomes acidic. After standing the solution for 5 mins white precipitate appears. The white precipitate was extracted with dichloromethane and dried over Na_2SO_4. The organic phase was concentrated under reduced pressure to obtain the 2,6-dichlorobenzyl alcohol (1g) in quantitative yield.

In the second step, A stirred solution of 2,6-dichlorobenzyl alcohol (1equiv.) in anhydrous DCM, PBr_3 (1.1equiv.) was added dropwise under cold condition. The reaction stirred for 12h at cold condition. After the end of the reaction, the reaction mixture was quenched by $NaHCO_3$ solution and extracted with EtOAc to produce the crude mass. The organic phase separated and dried over anhydrous Na_2SO_4 and concentrated under reduced pressure to obtain the pure 2,6-dichlorobenzyl bromide in quantitative yield.

In the third step, a stirred solution of the 2,6-dichlorobenzyl bromide (1.0 equiv.) in 10mL water/acetone (1:4) was added NaN_3 (4.0 equiv.). The resulting suspension was stirred at ambient temperature for 6h. Dichloromethane was added to the mixture and the organic layer was separated. The aqueous layer was extracted with 3X10mL aliquots of DCM and the combined organic phase was dried over anhydrous $NaSO_4$. The solvent was removed under reduced pressure and the obtained 2,6-dichlorobenzyl azide was sufficiently pure to use for the reaction without any purification.

B5) Typical reaction procedure for the reduction of 2,6-difluorobenzoyl chloride to 2,6-difluorobenzyl azide:

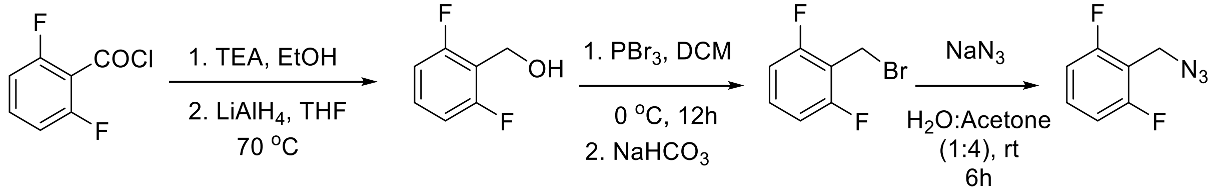

Figure S3: Reduction of 2,6-difluorobenzoyl chloride to 2,6-difluorobenzyl azide

The reaction was performed in three steps**:** In the first step, a 100 mL RBF containing 2,6-difluorobenzoyl chloride (1g, 5.66 mmol) in dry ethanol (25mL) was fitted with air condenser with magnetic stir bar and allowed to stir for 5mins at ambient conditions. To this solution, triethylamine (630mg, 6.22mmol) was added dropwise for 5 mins and white fumes appeared. The solution was then heated at 60 °C for 2h until complete conversion of 2,6-difluorobenzoyl chloride takes place. After the end of the reaction, the reaction mixture was with additional quantity of H_2O to quench the reaction. The organic phase was extracted with dichloromethane and dried over Na_2SO_4. The organic phase was then dried in vaccuo to get the final 2,6-difluoro ethyl benzoate in quantitative yield. The 2,6-difluoroethyl benzoate (1 equivalent) was dissolved in THF in a 100 mL RBF with a magnetic stir bar and placed over oil bath with a condenser. To this solution powdered $LiAlH_4$ (1 equivalent) was added portion wise and continue the reaction for 2h. At the end of the reaction excess water was added to quench and acidified with dilute HCl. The organic part was extracted by dichloromethane followed by evaporation under reduced pressure and the crude was purified by column chromatography to get the pure 2,6-difluorobenzyl alcohol in excellent yield. The second and third step of the reaction was similar as followed the same for B4. The quantitative yield of the 2,6-difluorobenzyl azide was observed.

(C) Synthesis and characterization data for the products triazoles (Table XX-XX)

1-(m-tolyl)-2-(4-(p-tolyl)-1H-1,2,3-triazol-1-yl)ethan-1-one (Table 1, 3a)

Prepared as described for B1, starting from **1a** (50 mg, 0.43mmol), **2a** (110mg, 0.52mmol) and NaN_3 (76.4mg, 0.52mmol) gave, after purification with silica gel column chromatography (20% EtOAc in n-Hexane) **3a** as white crystalline solid (105 mg, 86%). Mpt:140-142°C.

^{1}H NMR (400 MHz, DMSO-*d*6) δ 8.34 (s, 1H), 7.91 (d, *J* = 9.0 Hz, 2H), 7.76 (d, *J* = 8.0 Hz, 2H), 7.50 (dt, *J* = 14.9, 7.5 Hz, 2H), 7.25 (d, *J* = 8.0 Hz, 2H), 6.13 (s, 2H), 2.48 (s, 3H), 2.40 (s, 3H).
13C NMR (101 MHz, DMSO-*d*6) δ 192.73, 146.84, 138.95, 137.62, 135.35, 134.66, 129.97, 129.37, 129.03, 128.48, 125.94, 125.58, 123.09, 56.42, 21.33, 21.21.
GC-MS (m/z): 291 [M].

1-phenyl-2-(4-phenyl-1H-1,2,3-triazol-1-yl)ethan-1-one (Table 2, 3b)

Prepared as described for B1, starting from **1b** (100 mg, 0.9791 mmol), **2b** (233.8mg, 1.1749) and NaN_3 (76.4mg, 1.1749mmol) gave, after purification with silica gel column chromatography (20% EtOAc in n-Hexane) **3b** as white crystalline solid (188 mg, 73%).

^{1}H NMR (400 MHz, DMSO-*d*6) δ 8.42 (s, 1H), 8.12 (d, *J* = 7.3 Hz, 2H), 7.88 (d, *J* = 7.3 Hz, 2H), 7.73 (t, *J* = 7.4 Hz, 1H), 7.61 (t, *J* = 7.7 Hz, 2H), 7.45 (t, *J* = 7.6 Hz, 2H), 7.33 (t, *J* = 7.4 Hz, 1H), 6.19 (s, 2H).
GC-MS (m/z): 263 [M].

1-(4-methoxyphenyl)-2-(4-(thiophen-2-yl)-1H-1,2,3-triazol-1-yl)ethan-1-one (Table 2, 3c)

Prepared as described for B1, starting from **1c** (100 mg, 0.9245 mmol), **2c** (253mg, 1.10mmol) and NaN_3 (72mg, 1.10mmol) gave, after purification with silica gel column chromatography (20% EtOAc in n-Hexane) **3c** as yellow solid (160 mg, 58%).

^{1}H NMR (400 MHz, DMSO-*d*6) δ 8.43 (s, 1H), 8.08 (d, *J* = 8.8 Hz, 2H), 7.51 (dd, *J* = 32.3, 4.1 Hz, 3H), 7.15 (dd, *J* = 8.6, 3.3 Hz, 3H), 6.18 (s, 2H), 3.89 (s, 3H).
^{13}C NMR (101 MHz, DMSO-*d*6) δ 190.25, 163.94, 141.60, 133.02, 130.75, 130.46, 127.98, 126.91, 125.31, 124.07, 122.29, 114.13, 55.62, 40.16, 39.96, 39.75, 39.54, 39.33, 39.12, 38.91.
GC-MS (m/z): 299 [M].

1-(thiophen-2-yl)-2-(4-(p-tolyl)-1H-1,2,3-triazol-1-yl)ethan-1-one (Table 2, 3d)

Prepared as described for B1, starting from **1a** (100 mg, 0.8608 mmol), **2d** (212mg, 1.03mmol) and NaN_3 (67mg, 1.03mmol) gave, after purification with silica gel column chromatography (30% EtOAc in n-Hexane) **3d** as yellow solid (151 mg, 62%). Mpt:142-144°C.

^{1}H NMR (400 MHz, CDCl3) δ 7.94 (s, 1H), 7.88 (d, *J* = 3.7 Hz, 1H), 7.80 (d, *J* = 4.9 Hz, 1H), 7.76 (d, *J* = 7.9 Hz, 2H), 7.23-7.28 (m, 3H), 5.78 (s, 2H), 2.40 (s, 3H).
^{13}C NMR (101 MHz, DMSO-*d*6) δ 185.70, 146.87, 140.78, 137.68, 136.79, 135.15, 129.97, 129.61, 128.40, 125.59, 123.13, 55.94, 21.31.
GC-MS (m/z): 283 [M].

2-(4-([1,1'-biphenyl]-4-yl)-1H-1,2,3-triazol-1-yl)-1-(thiophen-2-yl)ethan-1-one (Table 2, 3e)

Prepared as described for B1, starting from **1d** (100 mg, 0.5610 mmol), **2d** (276mg, 1.34mmol) and NaN_3 (87mg, 1.34mmol) gave, after purification with silica gel column chromatography (40% EtOAc in n-Hexane) **3e** as brown solid (151 mg, 78%). Mpt:204-206^oC.

^{1}H NMR (400 MHz, $CDCl_3$) δ (ppm) 7.93 (s, 1H), 7.86 (d, *J* = 8.3 Hz, 2H), 7.80 (d, *J* = 3.3 Hz, 1H), 7.72 (d, *J* = 4.3 Hz, 1H), 7.58 (dd, *J* = 14.5, 7.8 Hz, 4H), 7.38 (t, *J* = 7.5 Hz, 2H), 7.28 (t, *J* = 7.3 Hz, 1H), 7.17 – 7.12 (m, 1H), 5.72 (s, 2H).

^{13}C NMR (101 MHz, $CDCl_3$) δ 183.25, 148.01, 141.03, 140.59, 140.42, 135.88, 133.26, 129.39, 128.83, 128.79, 127.54, 127.46, 127.01, 126.23, 121.33, 77.34, 77.02, 76.71, 55.45.
GC-MS (m/z): 345 [M].

2-(4-(4-(9H-carbazol-9-yl)phenyl)-1H-1,2,3-triazol-1-yl)-1-(3,4-difluorophenyl)ethan-1-one (Table 2, 3f)

Prepared as described for B1, starting from **1e** (100 mg, 0.374 mmol), **2e** (211mg, 0.897mmol) and NaN_3 (58.3mg, 0.897mmol) gave, after purification with silica gel column chromatography (30% EtOAc in n-Hexane) **3f** as off-white solid (161 mg, 93%). Mpt: 214-216^oC.

^{1}H NMR (400 MHz, $CDCl_3$) δ(ppm) 8.05 (dd, *J* = 21.7, 7.9 Hz, 4H), 7.94 (s, 1H), 7.86 – 7.73 (m, 2H), 7.58 (d, *J* = 8.2 Hz, 2H), 7.40 – 7.17 (m, 8H), 5.80 (s, 2H).

^{13}C NMR (101 MHz, $CDCl_3$) δ(ppm) 206.91, 187.98, 154.63 (dd, *J* = 260.5, 12.9 Hz), 150.85 (dd, *J* = 253.0, 13.0 Hz), 147.66, 140.75, 137.65, 130.97 (t, *J* = 4.0 Hz), 129.49, 127.37 (d, *J* = 17.1 Hz), 126.03, 125.42 (dd, *J* = 7.8, 3.6 Hz), 123.48, 121.55, 120.21 (d, *J* = 25.9 Hz), 118.35 (d, *J* = 18.1 Hz), 117.77 (dd, *J* = 18.3, 1.8 Hz), 109.79, 55.34, 30.91.
^{19}F NMR (376 MHz, $CDCl_3$) δ(ppm) -126.01 (d, *J* = 20.8 Hz), -134.01 (d, *J* = 20.9 Hz).
GC-MS (m/z): 464 [M].

2-(4-(4-(9H-carbazol-9-yl)phenyl)-1H-1,2,3-triazol-1-yl)-1-(thiophen-2-yl)ethan-1-one (Table 2, 3g)

Prepared as described for B1, starting from **1e** (100 mg, 0.374 mmol), **2d** (184mg, 0.897mmol) and NaN_3 (58.3mg, 0.897mmol) gave, after purification with silica gel column chromatography (30% EtOAc in n-Hexane) **3g** as brown solid (125 mg, 77%). Mpt:183-185^oC.

^{1}H NMR (400 MHz, $CDCl_3$) δ 8.27 – 8.07 (m, 5H), 7.93 (d, *J* = 3.6 Hz, 1H), 7.84 (d, *J* = 4.8 Hz, 1H), 7.67 (d, *J* = 8.3 Hz, 2H), 7.46 (q, *J* = 8.1 Hz, 4H), 7.32 (t, *J* = 7.1 Hz, 2H), 7.26 (d, *J* = 4.2 Hz, 1H), 5.86 (s, 2H).

^{13}C NMR (101 MHz, $CDCl_3$) δ 183.13, 147.58, 140.78, 140.37, 137.58, 135.95, 133.26, 129.60, 128.81, 127.45, 127.29, 126.02, 123.47, 121.55, 120.33, 120.06, 109.81, 55.46.
GC-MS (m/z): 434 [M].

2-(4-(4-(9H-carbazol-9-yl)phenyl)-1H-1,2,3-triazol-1-yl)-1-(naphthalen-2-yl)ethan-1-one (Table 2, 3h)

Prepared as described for B1, starting from **1e** (100 mg, 0.374 mmol), **2f** (223.5mg, 0.897mmol) and NaN_3 (58.3mg, 0.897mmol) gave, after purification with silica gel column chromatography (30% EtOAc in n-Hexane) **3h** as light yellow solid (132 mg, 74%). Mpt: 226-228°C.

^{1}H NMR (400 MHz, DMSO-*d*6) δ 8.93 (s, 1H), 8.74 (s, 1H), 8.28 (d, *J* = 7.7 Hz, 1H), 8.21 (d, *J* = 8.4 Hz, 1H), 8.12 (dt, *J* = 16.8, 8.4 Hz, 2H), 7.83–7.65 (m, 2H), 7.48 (d, *J* = 3.3 Hz, 2H), 7.38–7.21 (m, 1H), 6.47 (s, 1H).
^{13}C NMR (101 MHz, $CDCl_3$) δ 197.30, 150.98, 145.30, 141.52, 140.73, 137.34, 136.67, 135.21, 134.94, 134.44, 133.92, 133.07, 132.51, 132.42, 132.08, 131.53, 128.58, 128.04, 125.77, 125.37, 114.94, 61.36.
GC-MS (m/z): 478 [M].

2-(4-(4-(9H-carbazol-9-yl)phenyl)-1H-1,2,3-triazol-1-yl)-1-(6-methoxynaphthalen-2-yl)ethan-1-one (Table 2, 3i)

Prepared as described for B1, starting from **1e** (50 mg, 0.187 mmol), **2g** (223.5mg, 0.224mmol) gave, after purification with silica gel column chromatography (30% EtOAc in n-Hexane) **3i** as yellow solid (49 mg, 52%).

^{1}H NMR (400 MHz, DMSO-d6) δ 8.83 (s, 1H), 8.72 (s, 1H), 8.28 (d, *J* = 7.5 Hz, 2H), 8.20 (d, *J* = 8.4 Hz, 2H), 8.13 – 7.98 (m, 3H), 7.75 (d, *J* = 8.5 Hz, 2H), 7.47 (d, *J* = 3.7 Hz, 5H), 7.36 – 7.28 (m, 3H), 6.41 (s, 2H), 3.95 (s, 3H).

GC-MS (m/z): 508 [M].

2-(4-(4-(9H-carbazol-9-yl)phenyl)-1H-1,2,3-triazol-1-yl)-1-(3,5-bis(trifluoromethyl) phenyl)ethan-1-one (Table 2, 3j)

Prepared as described for B1, starting from **1e** (50 mg, 0.187 mmol), **2h** (75mg, 0.224mmol) and NaN_3 (36mg, 0.561mmol) gave, after purification with silica gel column chromatography (30% EtOAc in n-Hexane) **3j** as yellow solid (72 mg, 57%).

1H NMR (400 MHz, DMSO-*d*6) δ 8.69 (s, 2H), 8.54 (s, 1H), 8.34 (s, 1H), 8.19 (d, *J* = 6.4 Hz, 4H), 7.70 (d, *J* = 8.3 Hz, 2H), 7.50 – 7.35 (m, 4H), 7.28 (t, *J* = 7.8 Hz, 2H), 6.43 (s, 2H).

^{13}C NMR (101 MHz, DMSO-*d*6) δ 191.30, 146.30, 140.54, 136.84, 131.64, 131.30, 130.32, 129.43 (d, *J* = 4.2 Hz), 127.69, 127.37, 126.79, 124.80, 123.77, 123.29, 122.09, 121.04, 120.63, 110.19, 56.93, 31.15.

GC-MS (m/z): 564 [M].

1-(3,4-difluorophenyl)-2-(4-(4-(pentyloxy)phenyl)-1H-1,2,3-triazol-1-yl)ethan-1-one (Table 2, 3k)

Prepared as described for B1, starting from **1f** (100mg, 0.531mmol), **2e** (115mg, 0.584mmol) gave, after purification with silica gel column chromatography (20% EtOAc in n-Hexane) **3k** as white solid (167 mg, 82%).

1H NMR (400 MHz, DMSO-*d6*) δ 8.39 (s, 1H), 8.24-8.11 (m, 1H), 8.16-8.20 (m, 1H), 7.84-7.64 (m, 3H), 7.02 (d, *J* = 8.7 Hz, 2H), 6.23 (s, 2H), 4.01 (t, *J* = 6.5 Hz, 2H), 1.82-1.64 (m, 2H), 1.50-1.25 (m, 4H), 0.91 (t, *J* = 7.0 Hz, 3H).

^{13}C NMR (101 MHz, DMSO-*d6*) δ 158.95, 153.67 (dd, *J* = 255.0, 12.6 Hz), 150.06 (dd, *J* = 248.2, 13.1 Hz), 146.81, 132.10, 126.99, 126.72 (dd, *J* = 8.0, 3.4 Hz)., 123.62, 122.44, 118.82 (d, *J* = 17.8 Hz), 118.21 (d, *J* = 17.6 Hz), 115.32, 67.96, 56.40, 31.14, 28.85, 28.19, 22.37, 14.37.

^{19}F NMR (376 MHz, DMSO-*d6*) δ -129.70 (dd, *J* = 22.0, 3.1 Hz), -136.85 (dd, *J* = 22.0, 3.1 Hz).

GC-MS (m/z): 385 [M].

4-phenyl-1-tosyl-1H-1,2,3-triazole (Table 3, 5a)

Prepared as described for B1, starting from **1b** (100 mg, 0.97 mmol), 4a (231.7mg, 1.17mmol) gave, after purification with silica gel column chromatography (10% EtOAc in n-Hexane) **5a** as off-white solid (228 mg, 78%). Mpt:115-117°C.

^{1}H NMR (400 MHz, $CDCl_3$) δ 8.35 (s, 1H), 7.87 (d, *J* = 7.60 Hz, 2H), 7.44-7.51 (m, 4H), 7.38-7.34 (m, 1H), 7.13 (d, *J* = 7.60 Hz, 2H), 2.30 (s, 3H).
^{13}C NMR (101 MHz, DMSO-*d6*) δ 145.30, 145.07, 137.84, 130.22, 128.89, 128.10(2C), 125.52, 125.47(2C), 20.75.
GC-MS (m/z): 299 [M].

4-(thiophen-2-yl)-1-tosyl-1H-1,2,3-triazole (Table 3, 5b)

Prepared as described for B1, starting from **1c** (100 mg, 0.92 mmol), 4a (218.8mg, 1.10mmol) gave, after purification with silica gel column chromatography (10% EtOAc in n-Hexane) **5b** as pinkish white solid (233 mg, 83%).

^{1}H NMR (400 MHz, DMSO-*d6*) δ 8.24 (s, 1H), 7.56 (d, *J* = 4.7 Hz, 1H), 7.50 (d, *J* = 8.0 Hz, 3H), 7.13 (d, *J* = 7.8 Hz, 3H), 2.30 (s, 3H).
GC-MS (m/z): 305 [M].

4-([1,1'-biphenyl]-4-yl)-1-tosyl-1H-1,2,3-triazole (Table 3, 5c)

Prepared as described for B1, starting from **1d** (100 mg, 0.56 mmol), 4a (265.5mg, 1.346mmol) gave, after purification with silica gel column chromatography (20% EtOAc in n-Hexane) **5c** as white solid (132 mg, 63%). Mpt:147-149°C.

^{1}H NMR (400 MHz, DMSO-*d6*) δ 8.41 (s, 1H), 7.97 (d, *J* = 8.1 Hz, 2H), 7.75 (dd, *J* = 19.4, 7.8 Hz, 4H), 7.50 (m, 4H), 7.38 (m, 1H), 7.14 (d, *J* = 7.8 Hz, 2H), 2.30 (s, 3H).

^{13}C NMR (101 MHz, DMSO-*d6*) δ 145.23, 139.70, 139.52, 137.90, 129.31, 129.04, 128.95, 128.13, 127.56, 127.13, 126.52, 126.08, 125.48, 121.13, 20.75.
GC-MS (m/z): 375 [M].

9-(4-(1-tosyl-1H-1,2,3-triazol-4-yl)phenyl)-9H-carbazole (Table 3, 5d)

Prepared as described for B1, starting from **1e** (100 mg, 0.374 mmol), 4a (88.5mg, 0.448mmol) gave, after purification with silica gel column chromatography (20% EtOAc in n-Hexane) **5a** as light yellow solid (114.7 mg, 66%).

^{1}H NMR (400 MHz, $CDCl_3$) δ 8.33 (s, 1H), 8.03 (m, 6H), 7.58 (d, *J* = 7.9 Hz, 2H), 7.35 (m, 5H), 7.20 (d, *J* = 17.1 Hz, 3H), 2.39 (s, 3H).

^{13}C NMR (101 MHz, $CDCl_3$) δ 147.51, 146.63, 140.62, 138.43, 133.05, 130.54, 128.80, 127.92, 127.60, 127.50, 126.07, 123.57, 120.39, 120.22, 119.13, 109.71, 77.34, 77.22, 77.02, 76.70, 21.88.
GC-MS (m/z): 464 [M].

4-(4-(pentyloxy)phenyl)-1-tosyl-1H-1,2,3-triazole (Table 3, 5e)

Prepared as described for B1, starting from **1f** (100mg, 0.531mmol), **4a** (126mg, 0.638mmol) gave, after purification with silica gel column chromatography (20% EtOAc in n-Hexane) **5e** as white solid (196 mg, 96%).

^{1}H NMR (400 MHz, DMSO-*d6*) δ 8.22 (s, 1H), 7.77 (d, *J* = 8.6 Hz, 2H), 7.50 (d, *J* = 7.9 Hz, 2H), 7.13 (d, *J* = 7.9 Hz, 2H), 7.01 (d, *J* = 8.7 Hz, 2H), 4.00 (t, *J* = 6.5 Hz, 2H), 2.30 (s, 3H), 1.86 – 1.60 (m, 2H), 1.47 – 1.25 (m, 4H), 0.91 (t, *J* = 6.9 Hz, 3H).

^{13}C NMR (101 MHz, DMSO-*d6*) δ 159.16, 145.80, 138.34, 128.61, 127.41, 125.98, 122.91, 115.31, 67.97, 28.83, 28.17, 22.36, 21.25, 14.38.

GC-MS (m/z): 385 [M].

ethyl 1-(2,6-dichlorobenzyl)-1H-1,2,3-triazole-4-carboxylate (Scheme 1, 6e)

Prepared as described for B1, starting from **6d** (100 mg, 0.495 mmol), ethylpropiolate (60µlt, 0.594mmol) gave, after purification with silica gel column chromatography (30% EtOAc in n-Hexane) **6e** as light yellow solid (99.5 mg, 67%).

^{1}H NMR (600 MHz, DMSO-*d6*) *δ* 8.82 (s, 1H), 7.57 (d, *J* = 8.2 Hz, 2H), 7.50 – 7.45 (m, 1H), 5.87 (s, 2H), 4.30 (q, *J* = 7.1 Hz, 2H), 1.29 (t, *J* = 7.1 Hz, 3H).
13C NMR (151 MHz, DMSO-*d6*) *δ* 160.62, 138.97, 136.45, 132.24, 130.36, 130.00, 129.44, 61.07, 49.41, 14.60.

ethyl 1-(2,6-difluorobenzyl)-1H-1,2,3-triazole-4-carboxylate (Scheme 1, 7e)

Prepared as described for B1, starting from **7d** (100 mg, 0.591 mmol), ethylpropiolate (72µlt, 0.709 mmol) gave, after purification with silica gel column chromatography (40% EtOAc in n-Hexane) **7e** as light yellow solid (124.6 mg, 79%).

^{1}H NMR (400 MHz, DMSO-*d6*) *δ* 8.84 (s, 1H), 7.59 – 7.46 (m, 1H), 7.19 (t, *J* = 8.1 Hz, 2H), 5.74 (s, 2H), 4.31 (q, *J* = 7.1 Hz, 2H), 1.30 (t, *J* = 7.1 Hz, 3H).
13C NMR (101 MHz, DMSO-*d6*) δ 162.02 (d, *J* = 7.3 Hz), 160.07, 159.54 (d, *J* = 7.3 Hz), 138.71, 131.84 (t, *J* = 10.4 Hz), 129.36, 112.02 (d, *J* = 5.7 Hz), 111.84 (d, *J* = 5.7 Hz), 110.81 (t, *J* = 19.0 Hz), 60.54, 41.29, 14.09.
GC-MS (m/z): 267 [M].

D. Spectral data (^{1}H, ^{13}C, GC-MS) for synthesized compounds (3a-k, 5a-e, 6e and 7e).

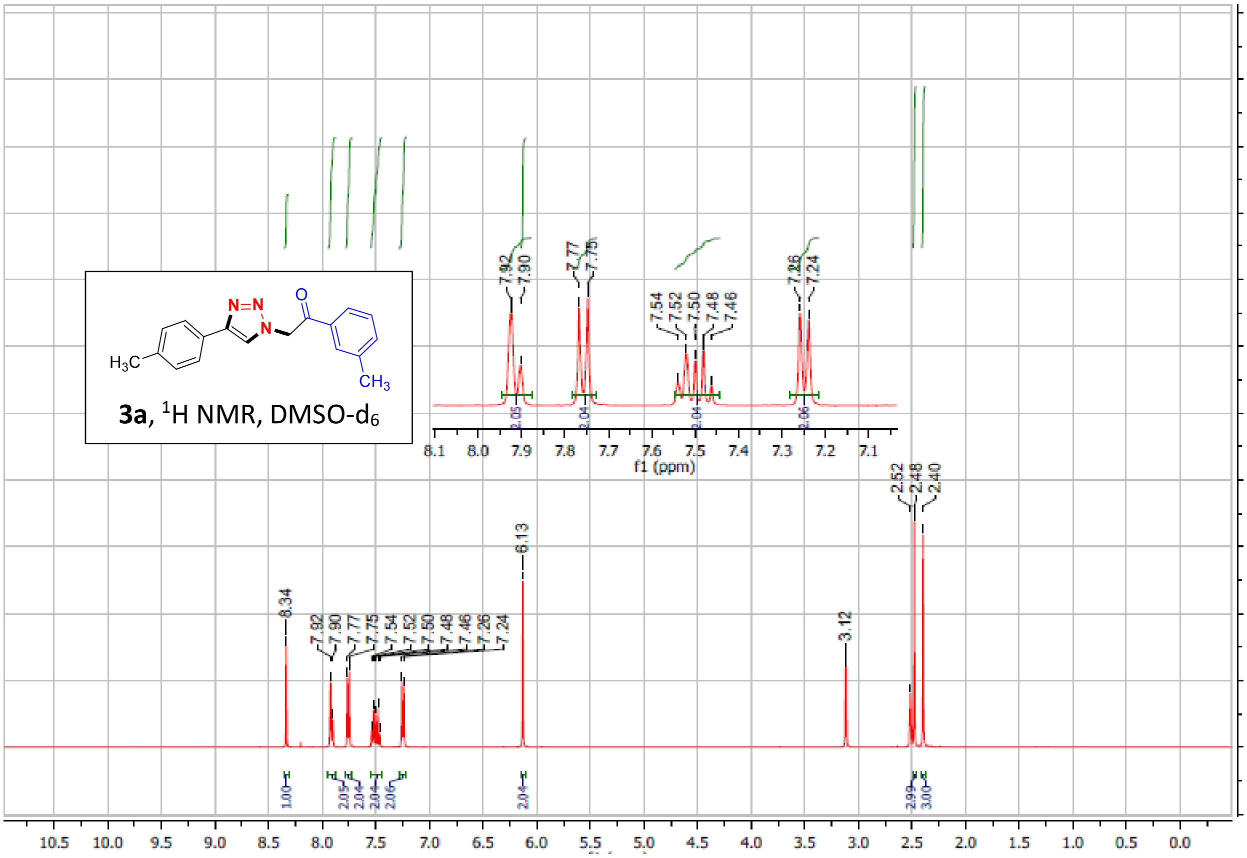

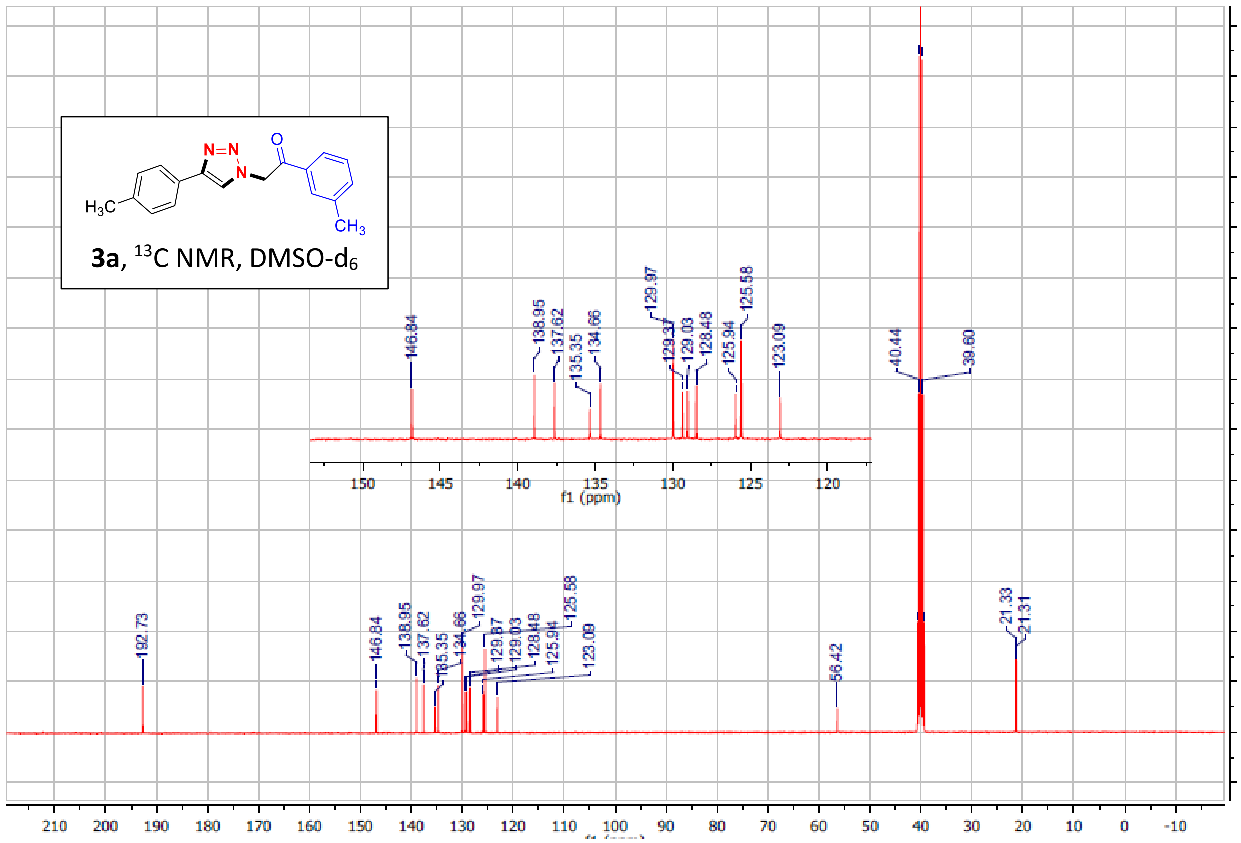
3a, ^{13}C NMR, DMSO-d$_6$
192.73
146.84
138.95
137.62
135.35
134.66
129.97
129.37
129.03
128.48
125.94
125.58
123.09
56.42
40.44
39.60
21.33
21.31
f1 (ppm)

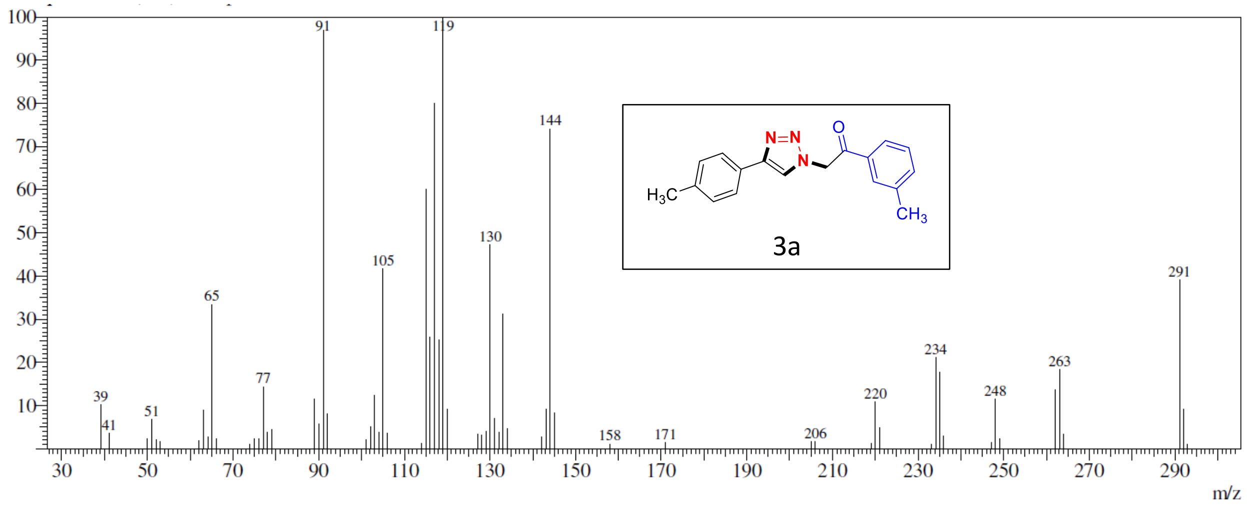
3a
91
119
144
130
105
65
77
39
41
51
158
171
206
220
234
248
263
291
m/z

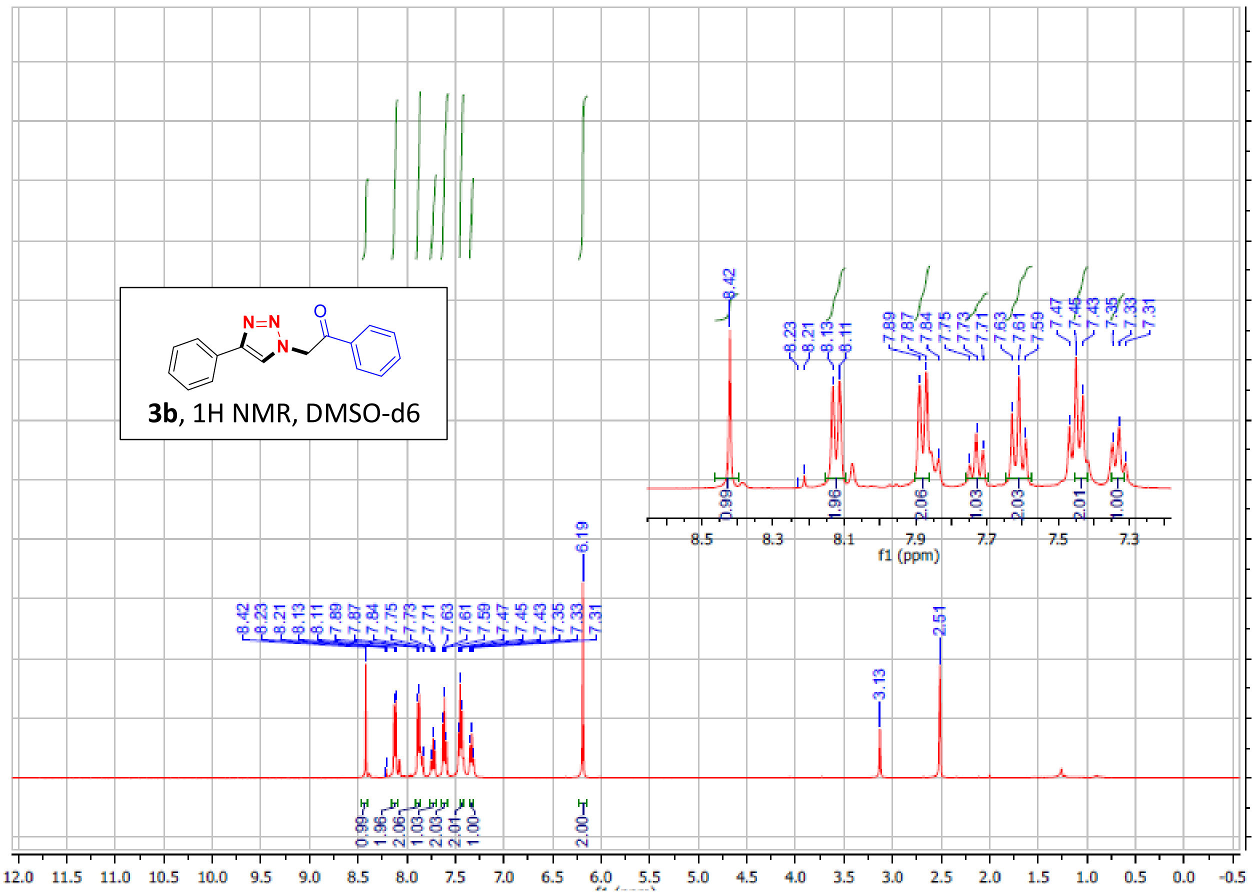
3b, 1H NMR, DMSO-d6
f1 (ppm)

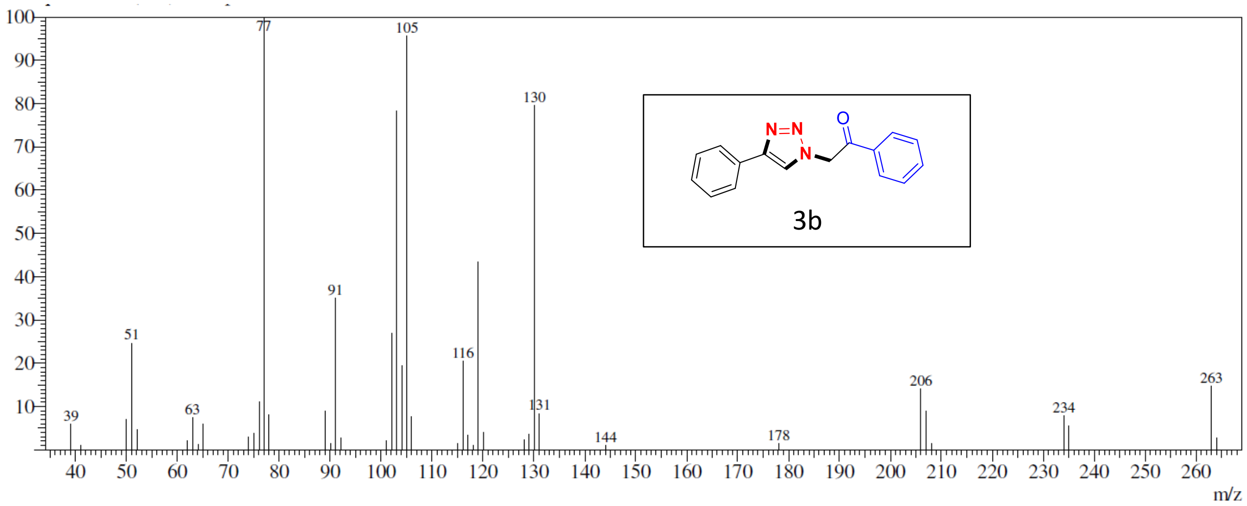
3b
m/z

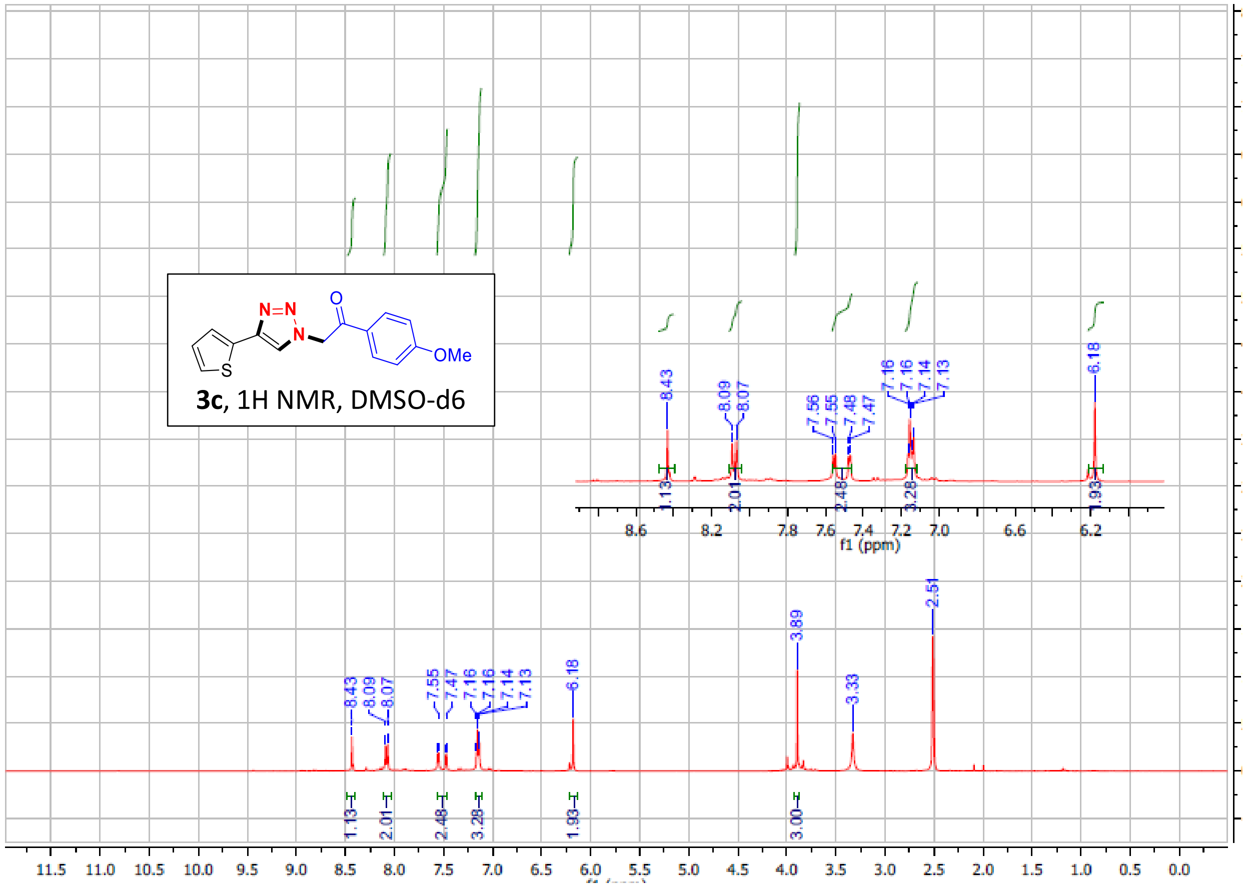
N=N
N
O
OMe
S
3c, 1H NMR, DMSO-d6
8.43
8.09
8.07
7.56
7.55
7.48
7.47
7.16
7.16
7.14
7.13
6.18
1.13
2.01
2.48
3.28
1.93
8.6
8.2
7.8
7.6
7.4
7.2
7.0
6.6
6.2
f1 (ppm)
3.89
3.33
2.51
3.00
11.5
11.0
10.5
10.0
9.5
9.0
8.5
8.0
7.5
7.0
6.5
6.0
5.5
5.0
4.5
4.0
3.5
3.0
2.5
2.0
1.5
1.0
0.5
0.0

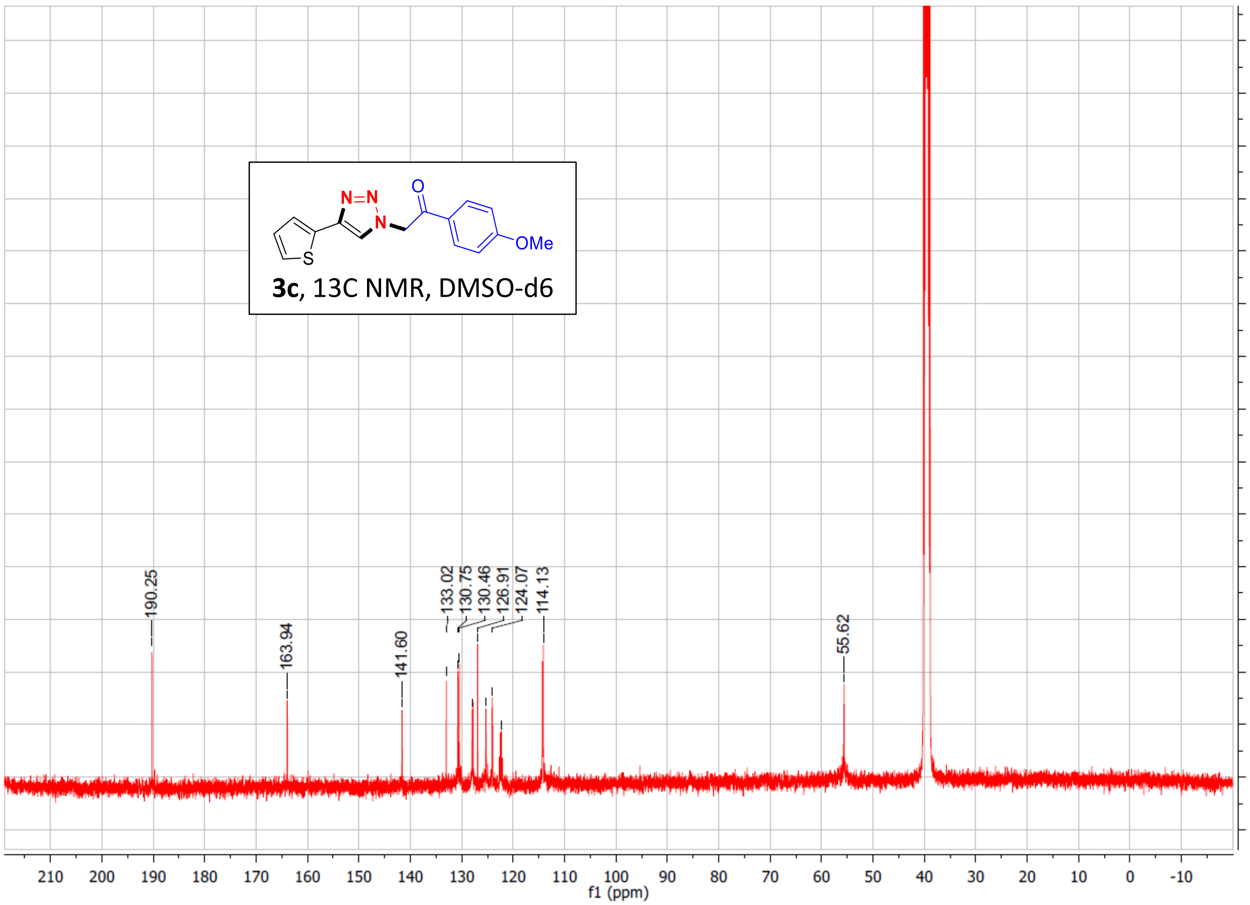
N=N
N
O
OMe
S
3c, 13C NMR, DMSO-d6
190.25
163.94
141.60
133.02
130.75
130.46
126.91
124.07
114.13
55.62
210 200 190 180 170 160 150 140 130 120 110 100 90 80 70 60 50 40 30 20 10 0 -10
f1 (ppm)

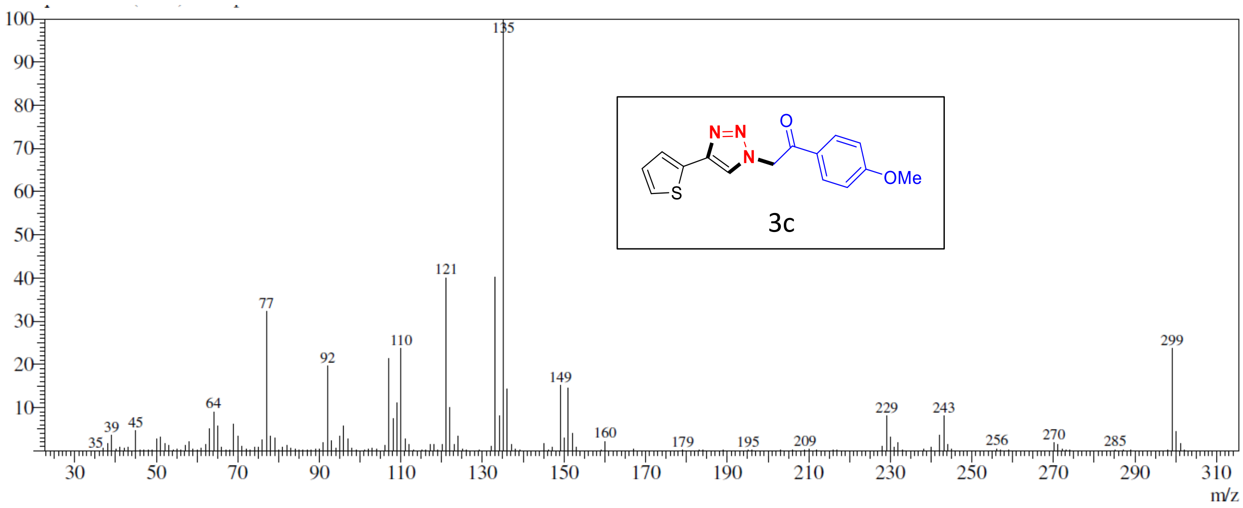
100 90 80 70 60 50 40 30 20 10
135
121
77
92
110
149
299
64
45
39
35
160
179
195
209
229
243
256
270
285
N=N
N
O
OMe
S
3c
30 50 70 90 110 130 150 170 190 210 230 250 270 290 310
m/z

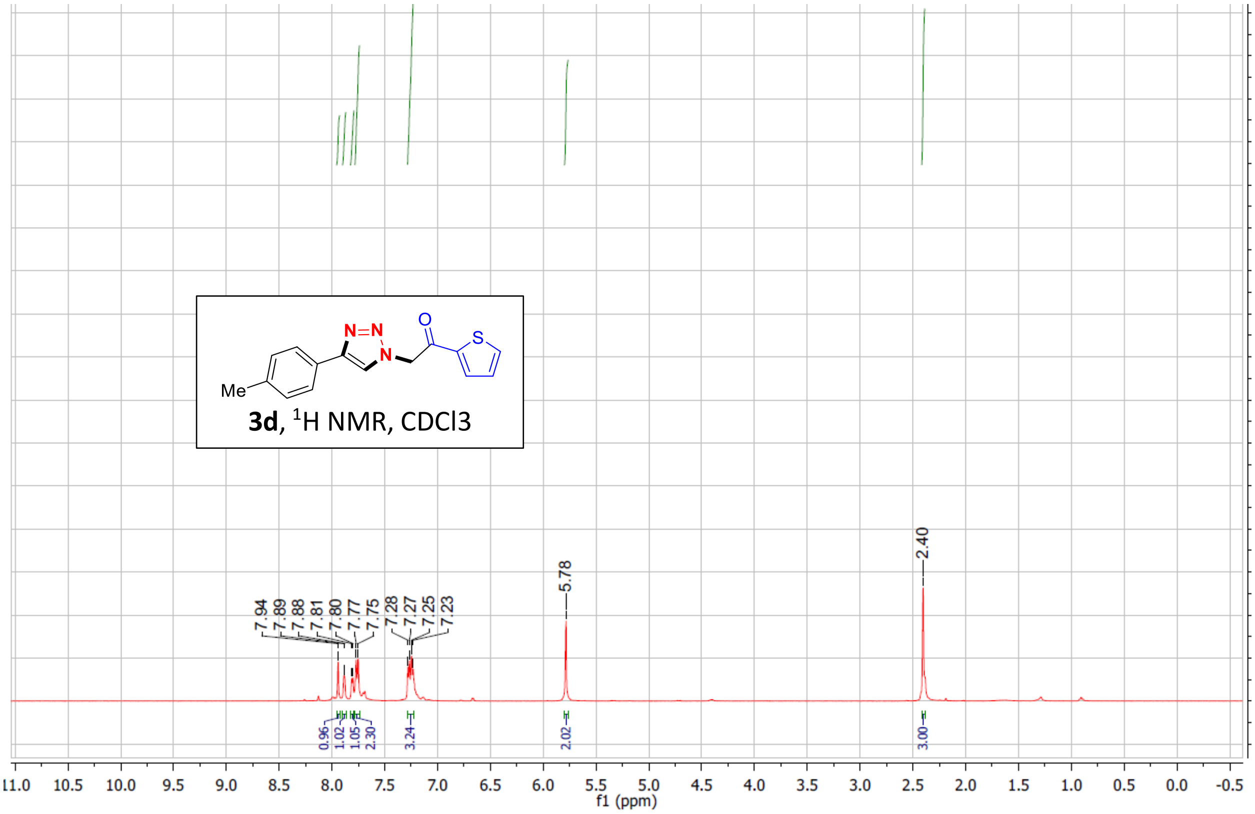

N=N
N
O
S
Me
3d, 1H NMR, CDCl3
7.94
7.89
7.88
7.81
7.80
7.77
7.75
7.28
7.27
7.25
7.23
5.78
2.40
0.96
1.02
1.05
2.30
3.24
2.02
3.00
11.0 10.5 10.0 9.5 9.0 8.5 8.0 7.5 7.0 6.5 6.0 5.5 5.0 4.5 4.0 3.5 3.0 2.5 2.0 1.5 1.0 0.5 0.0 -0.5
f1 (ppm)

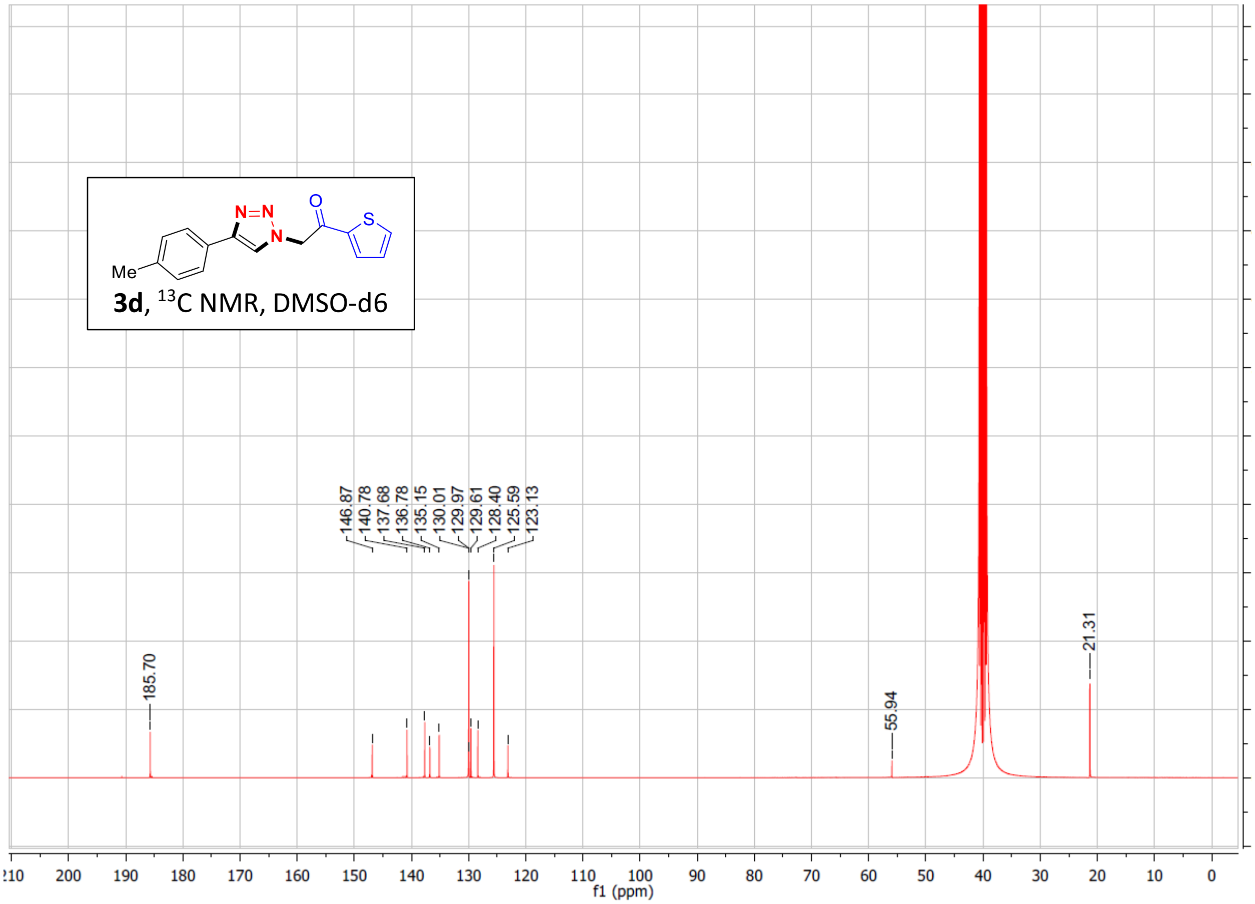

Me
N=N
N
O
S
3d, ^{13}C NMR, DMSO-d6
185.70
146.87
140.78
137.68
136.78
135.15
130.01
129.97
129.61
128.40
125.59
123.13
55.94
21.31
f1 (ppm)

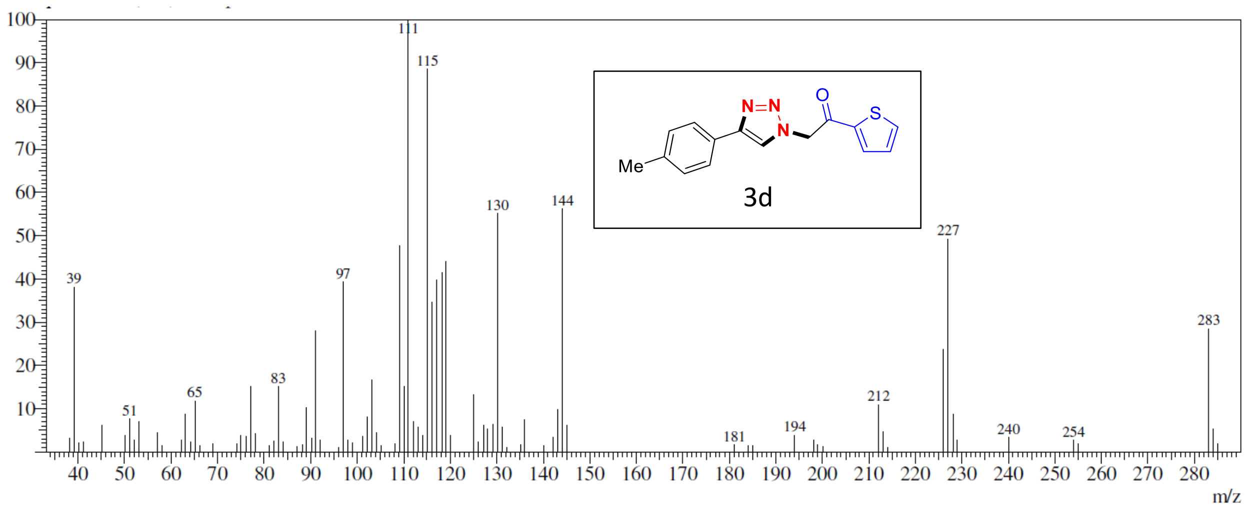

Me
N=N
N
O
S
3d
111
115
130
144
227
39
97
83
65
51
283
212
181
194
240
254
m/z

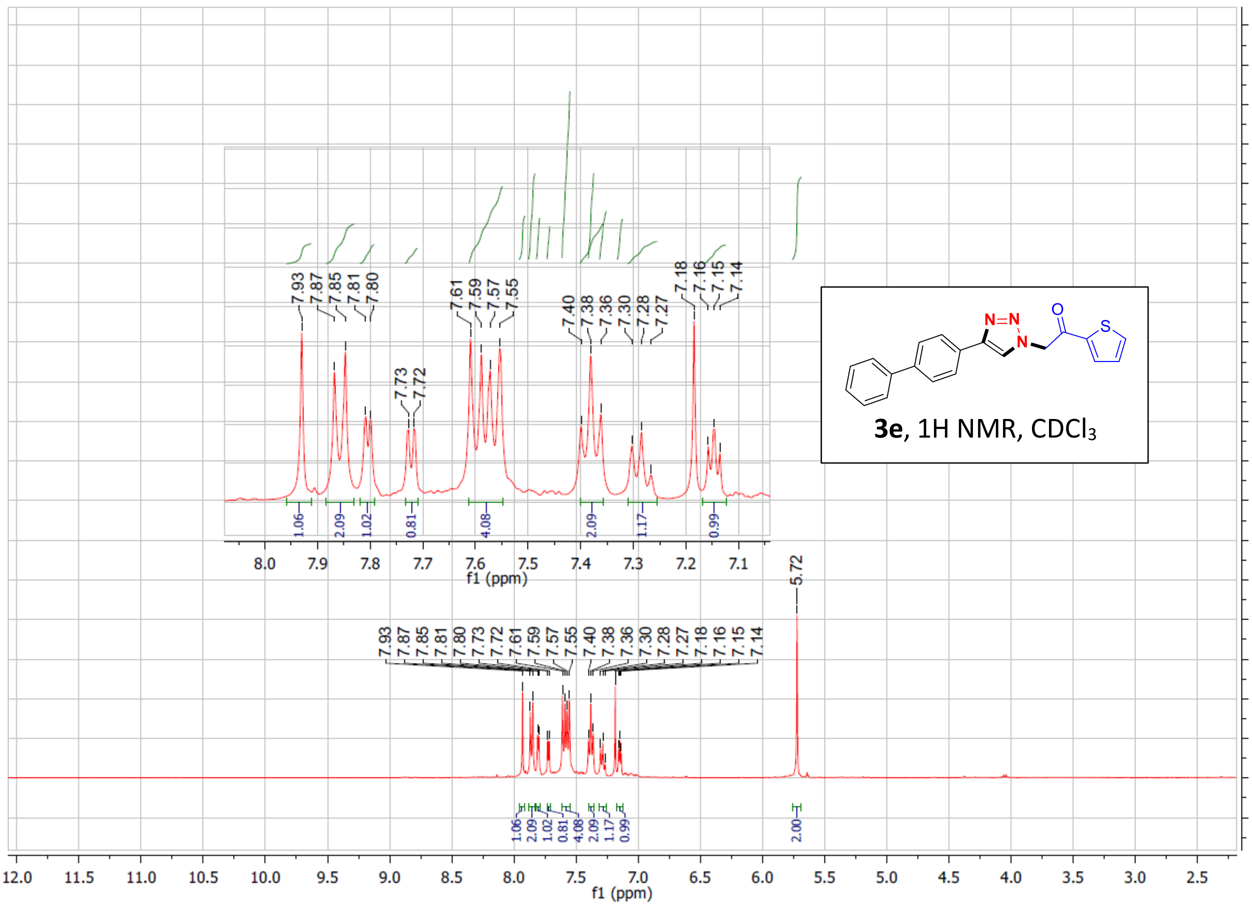
3e, 1H NMR, CDCl3
f1 (ppm)
5.72

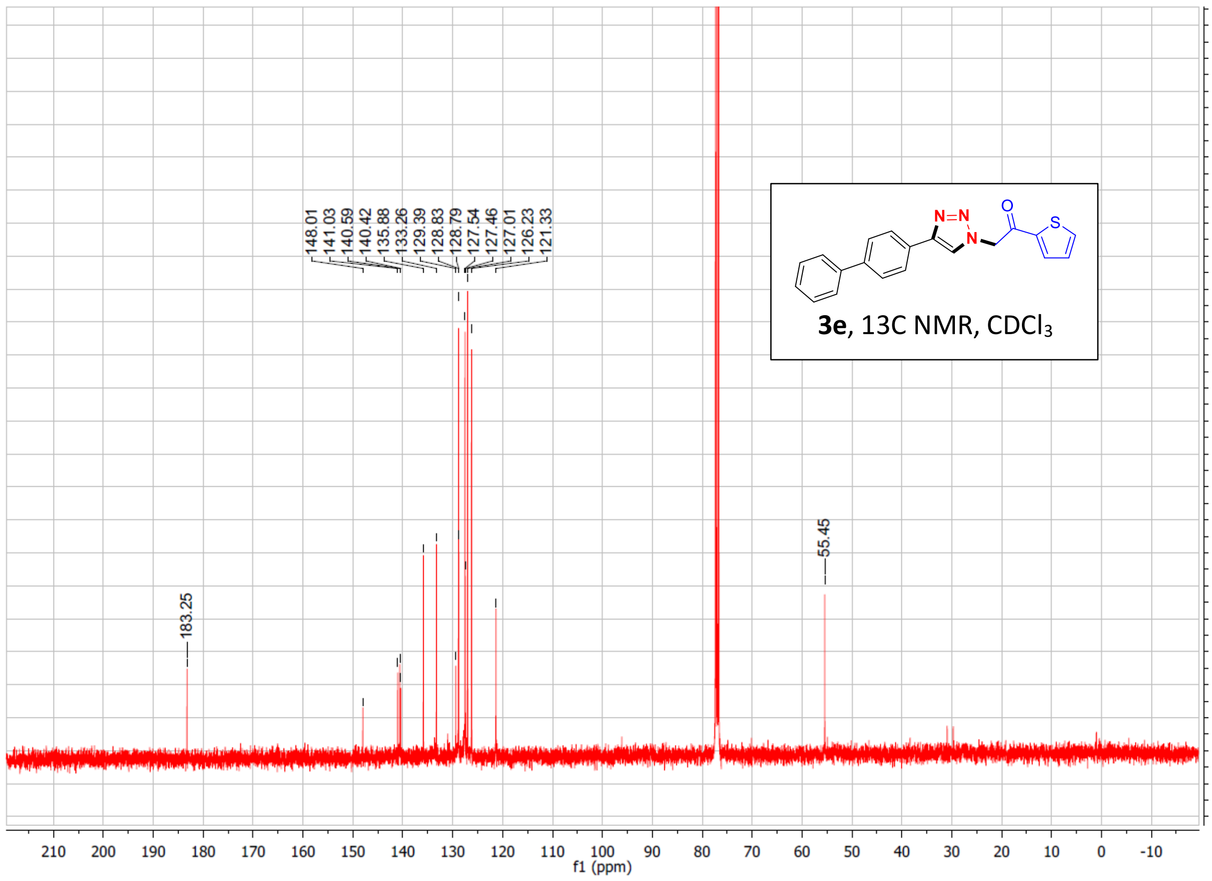
148.01
141.03
140.59
140.42
135.88
133.26
129.39
128.83
128.79
127.54
127.46
127.01
126.23
121.33
183.25
55.45
N=N
N
O
S
3e, 13C NMR, CDCl3
210 200 190 180 170 160 150 140 130 120 110 100 90 80 70 60 50 40 30 20 10 0 -10
f1 (ppm)

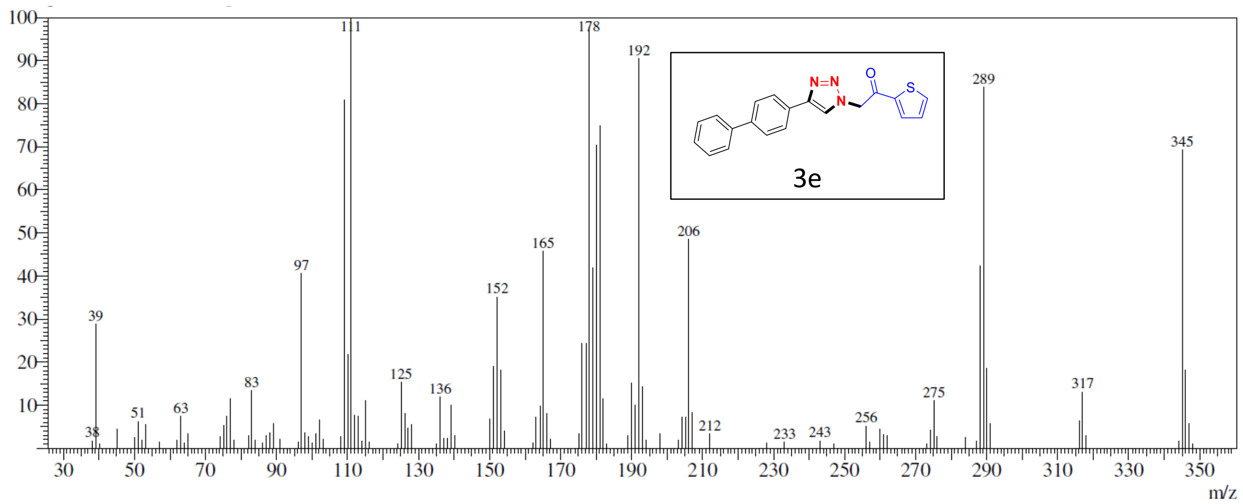
100
90
80
70
60
50
40
30
20
10
111
178
192
289
345
206
165
97
152
39
83
125
136
63
51
38
212
233
243
256
275
317
N=N
N
O
S
3e
30 50 70 90 110 130 150 170 190 210 230 250 270 290 310 330 350
m/z

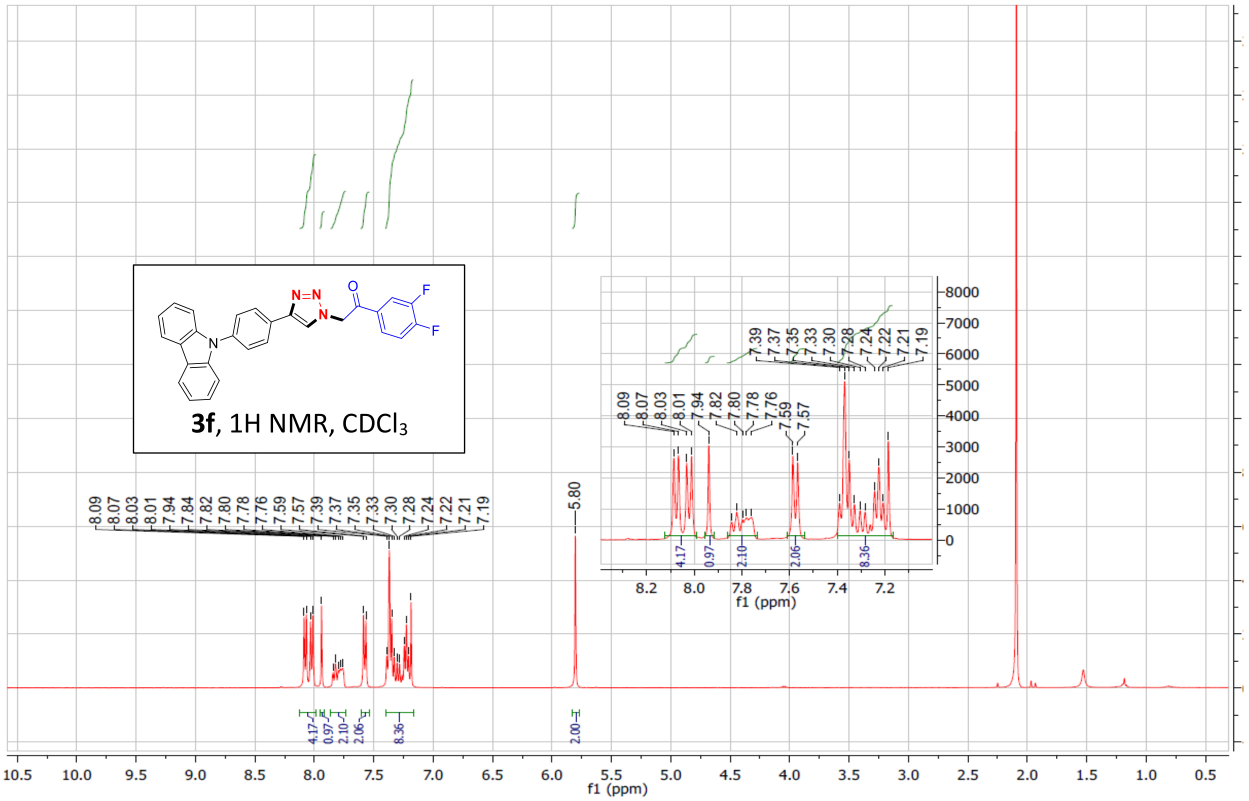
3f, 1H NMR, CDCl3
8.09 8.07 8.03 8.01 7.94 7.84 7.82 7.80 7.78 7.76 7.59 7.57 7.39 7.37 7.35 7.33 7.30 7.28 7.24 7.22 7.21 7.19
5.80
4.17 0.97 2.10 2.06 8.36 2.00
f1 (ppm)

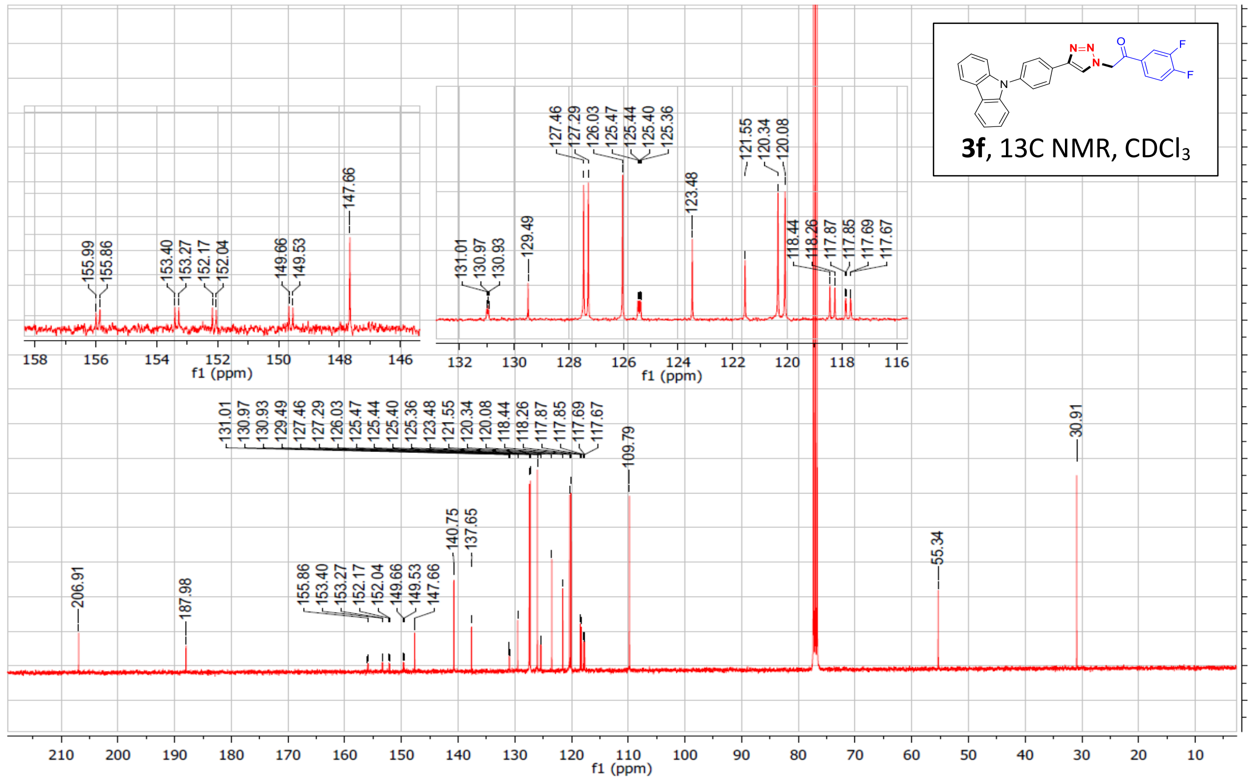
3f, 13C NMR, CDCl3
206.91
187.98
155.86 153.40 153.27 152.17 152.04 149.66 149.53 147.66
140.75 137.65
131.01 130.97 130.93 129.49 127.46 127.29 126.03 125.47 125.44 125.40 125.36 123.48 121.55 120.34 120.08 118.44 118.26 117.87 117.85 117.69 117.67
109.79
55.34
30.91
f1 (ppm)

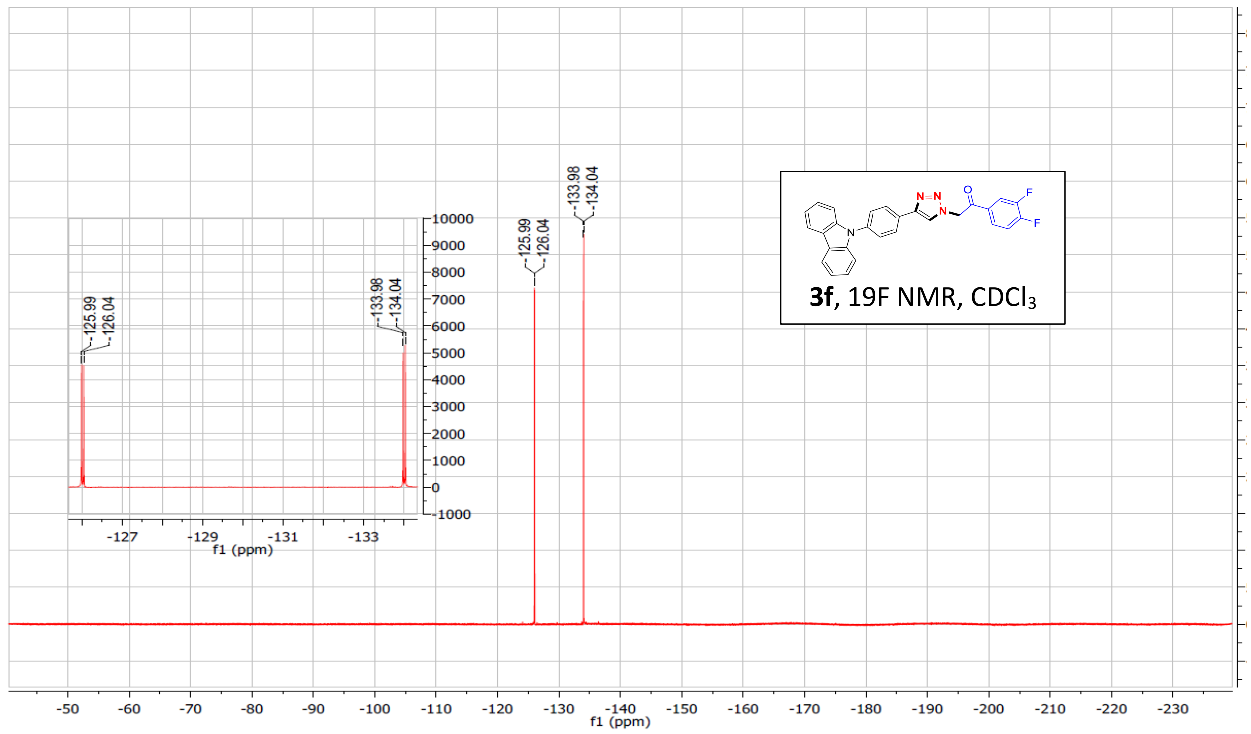
3f, 19F NMR, CDCl3
-125.99
-126.04
-133.98
-134.04
f1 (ppm)

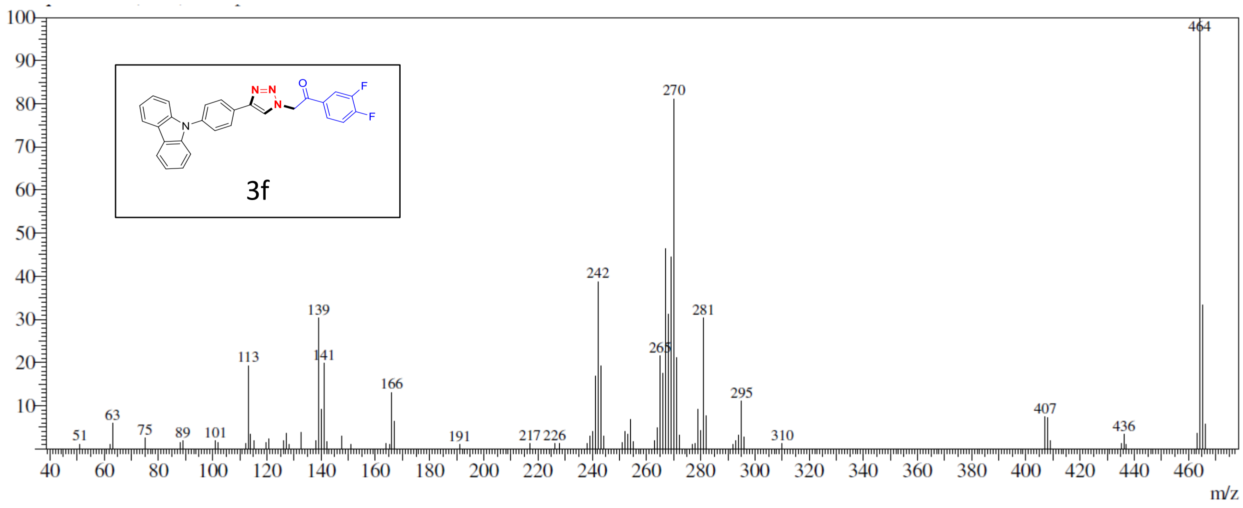
3f
464
270
242
281
139
141
113
265
166
295
407
436
63
75
89
101
51
191
217
226
310
m/z

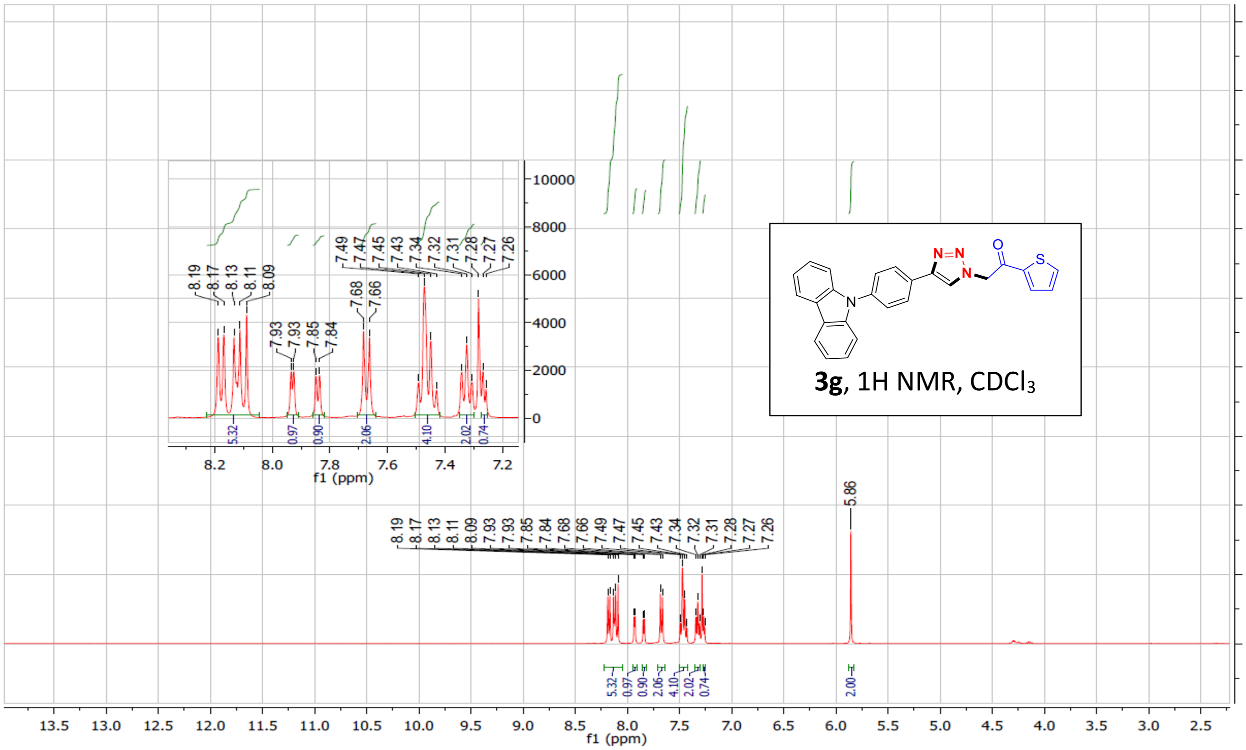
3g, 1H NMR, CDCl3
8.19 8.17 8.13 8.11 8.09 7.93 7.93 7.85 7.84 7.68 7.66 7.49 7.47 7.45 7.43 7.34 7.32 7.31 7.28 7.27 7.26
5.86
5.32 0.97 0.90 2.06 4.10 2.02 0.74
2.00
f1 (ppm)

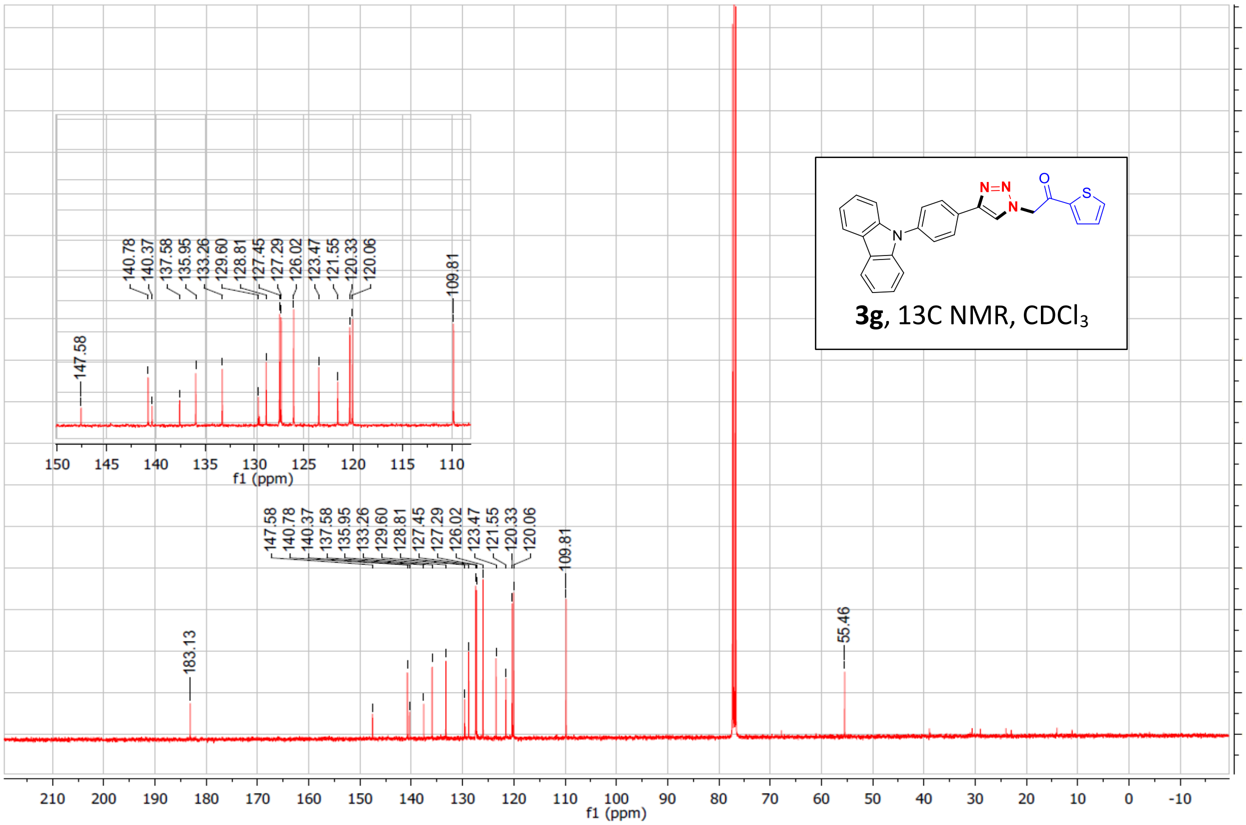
3g, 13C NMR, CDCl3
147.58 140.78 140.37 137.58 135.95 133.26 129.60 128.81 127.45 127.29 126.02 123.47 121.55 120.33 120.06
109.81
183.13
55.46
f1 (ppm)

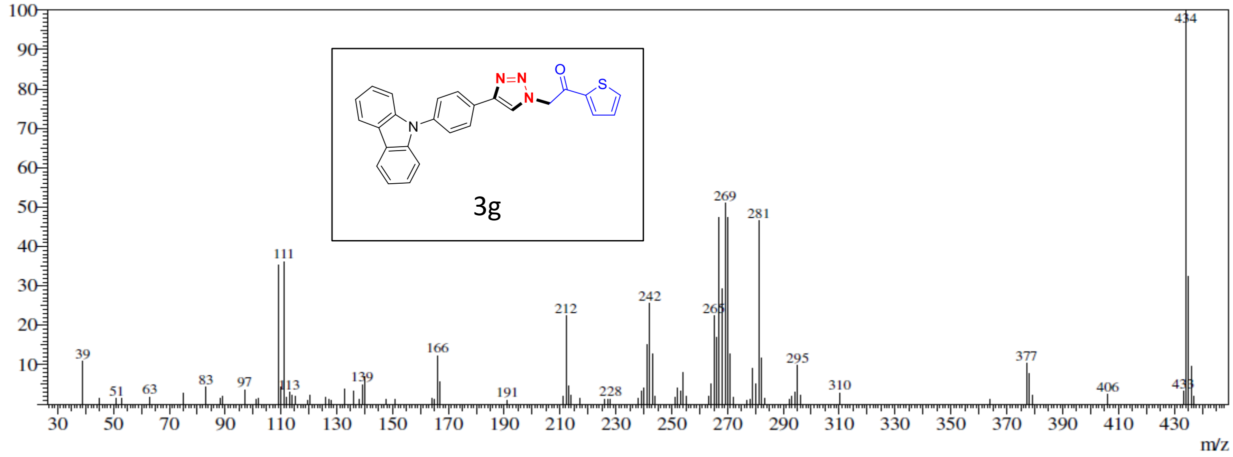
3g
m/z

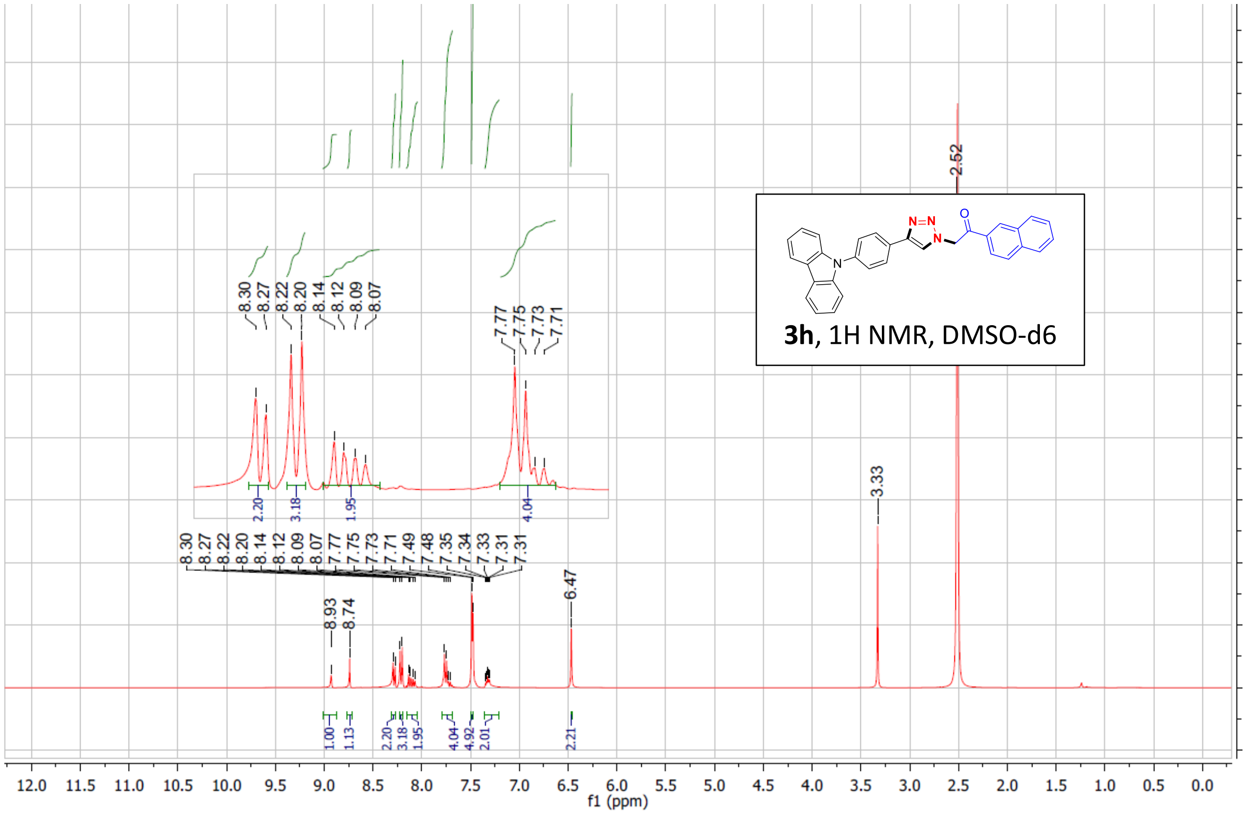
3h, 1H NMR, DMSO-d6
f1 (ppm)

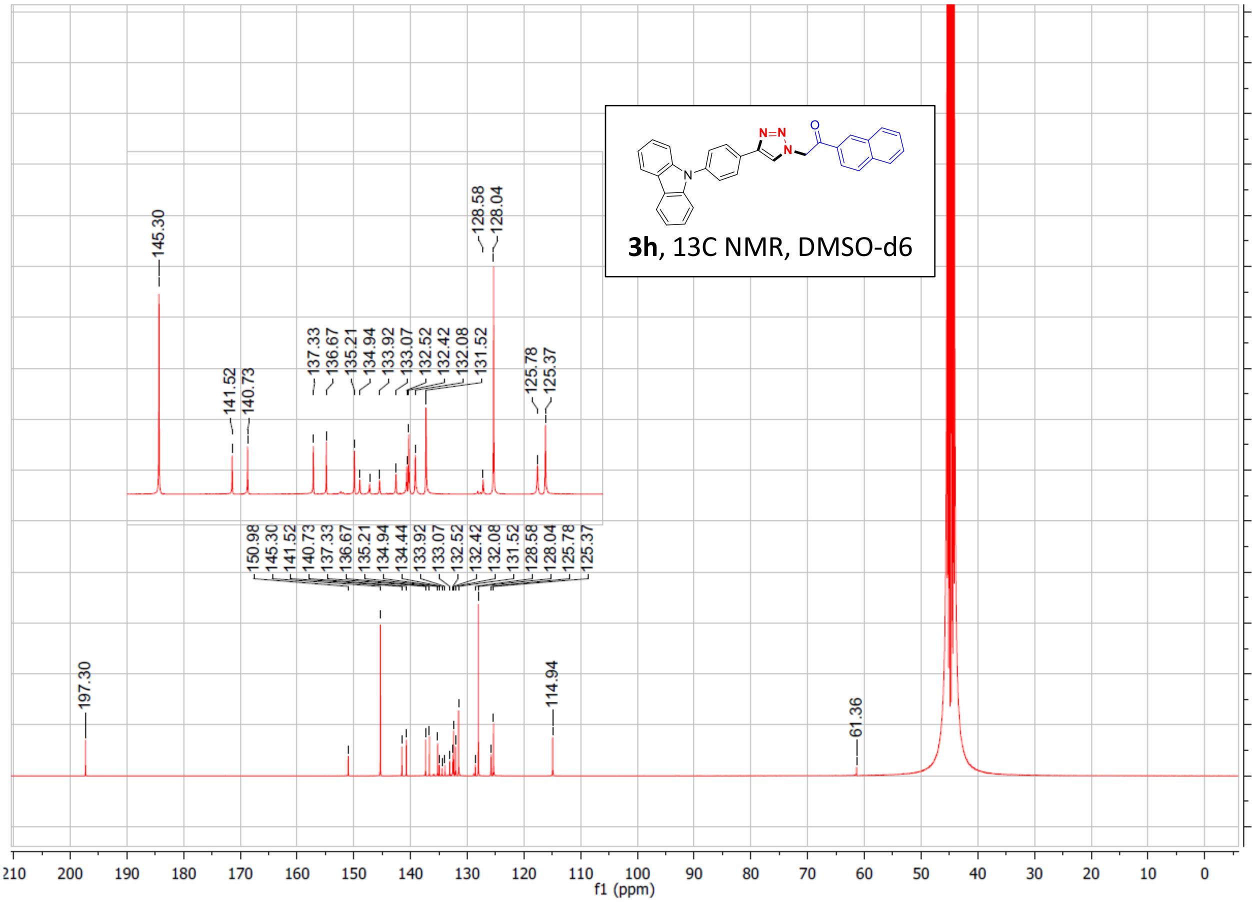
3h, 13C NMR, DMSO-d6
197.30
150.98
145.30
141.52
140.73
137.33
136.67
135.21
134.94
134.44
133.92
133.07
132.52
132.42
132.08
131.52
128.58
128.04
125.78
125.37
114.94
61.36
f1 (ppm)

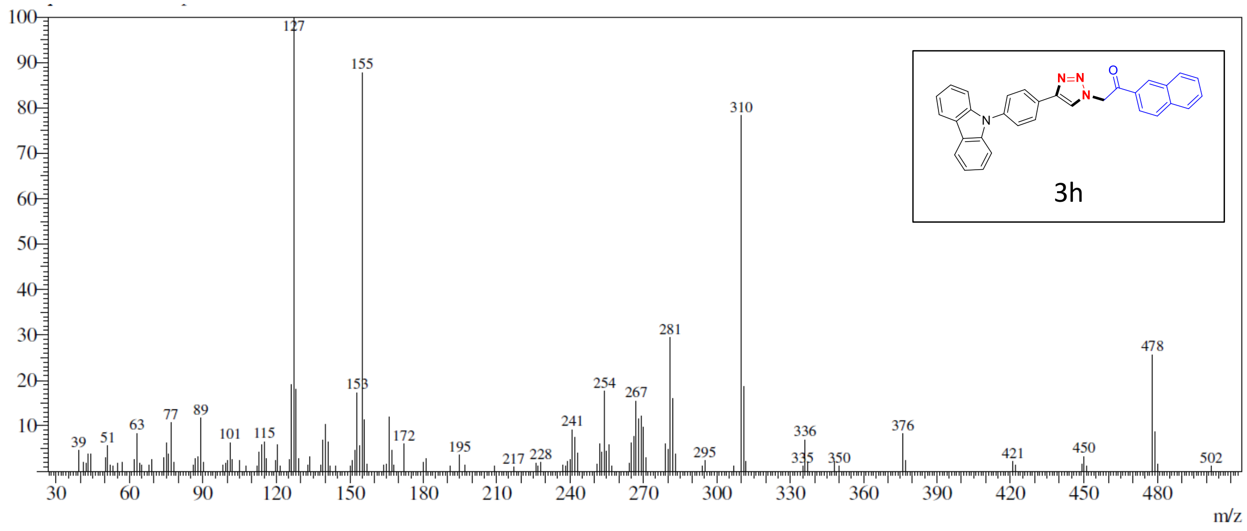
3h
127
155
310
281
478
153
254
267
241
39
51
63
77
89
101
115
172
195
217
228
295
336
335
350
376
421
450
502
m/z

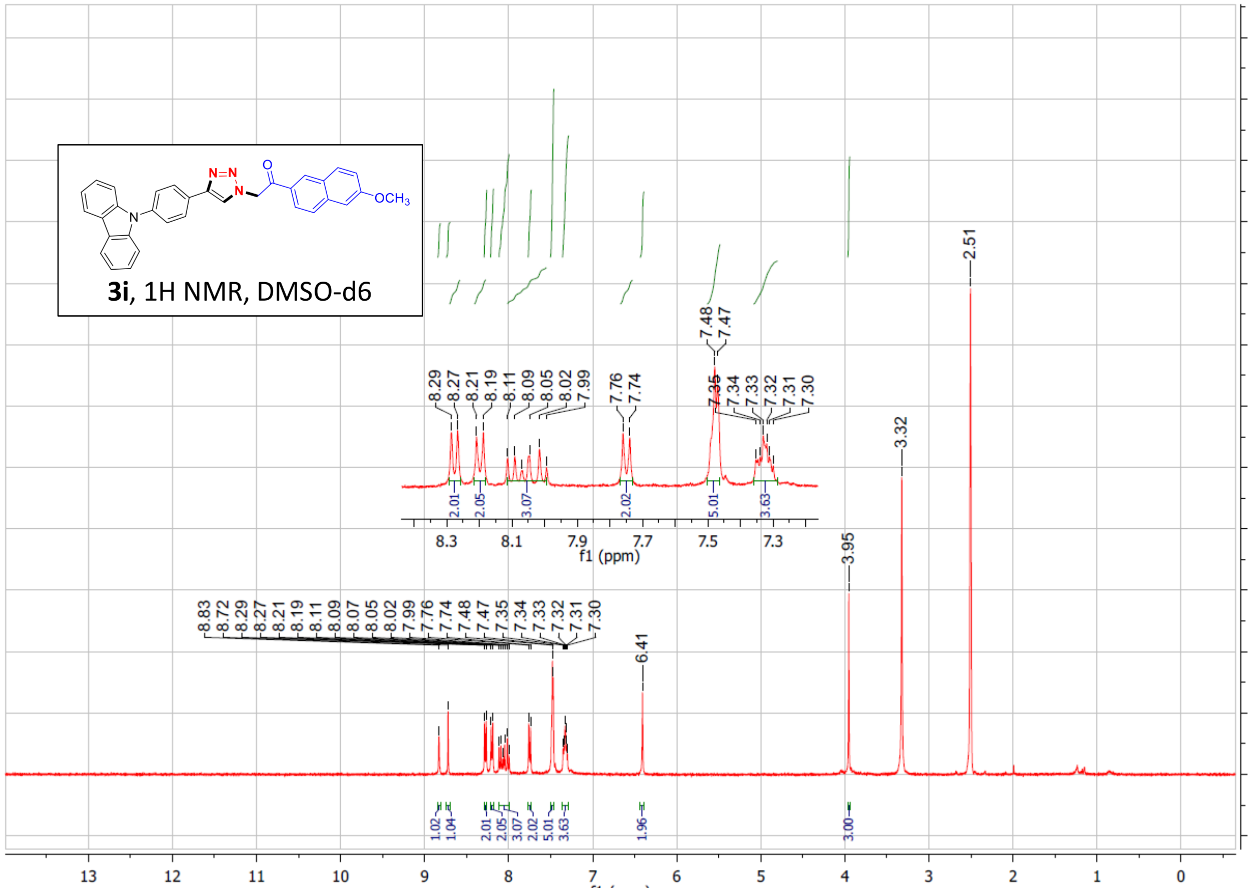
3i, 1H NMR, DMSO-d6
OCH3
2.51
3.32
3.95
6.41
f1 (ppm)

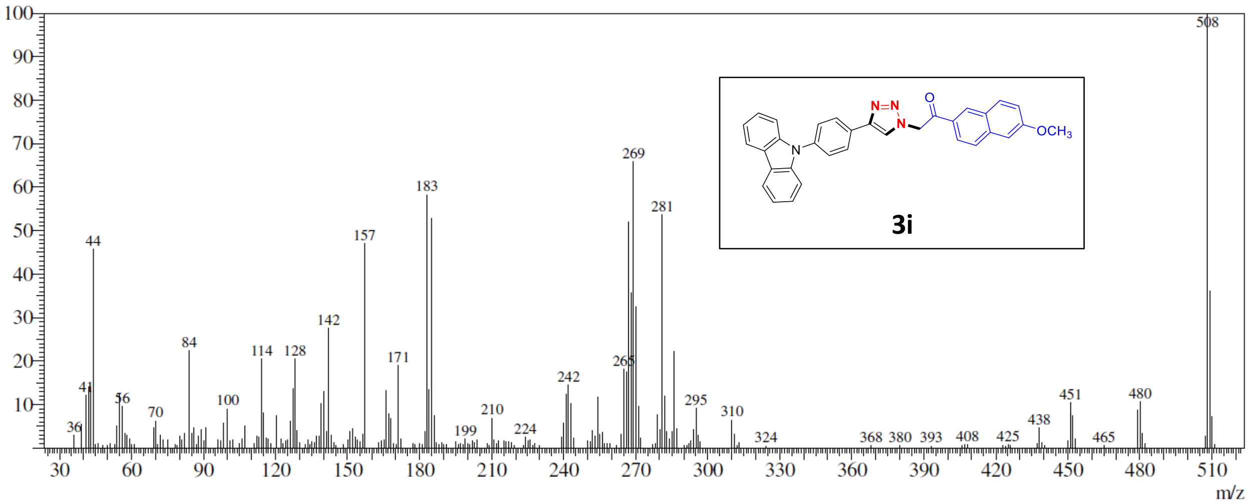
OCH3
3i
508
269
183
281
157
44
142
m/z

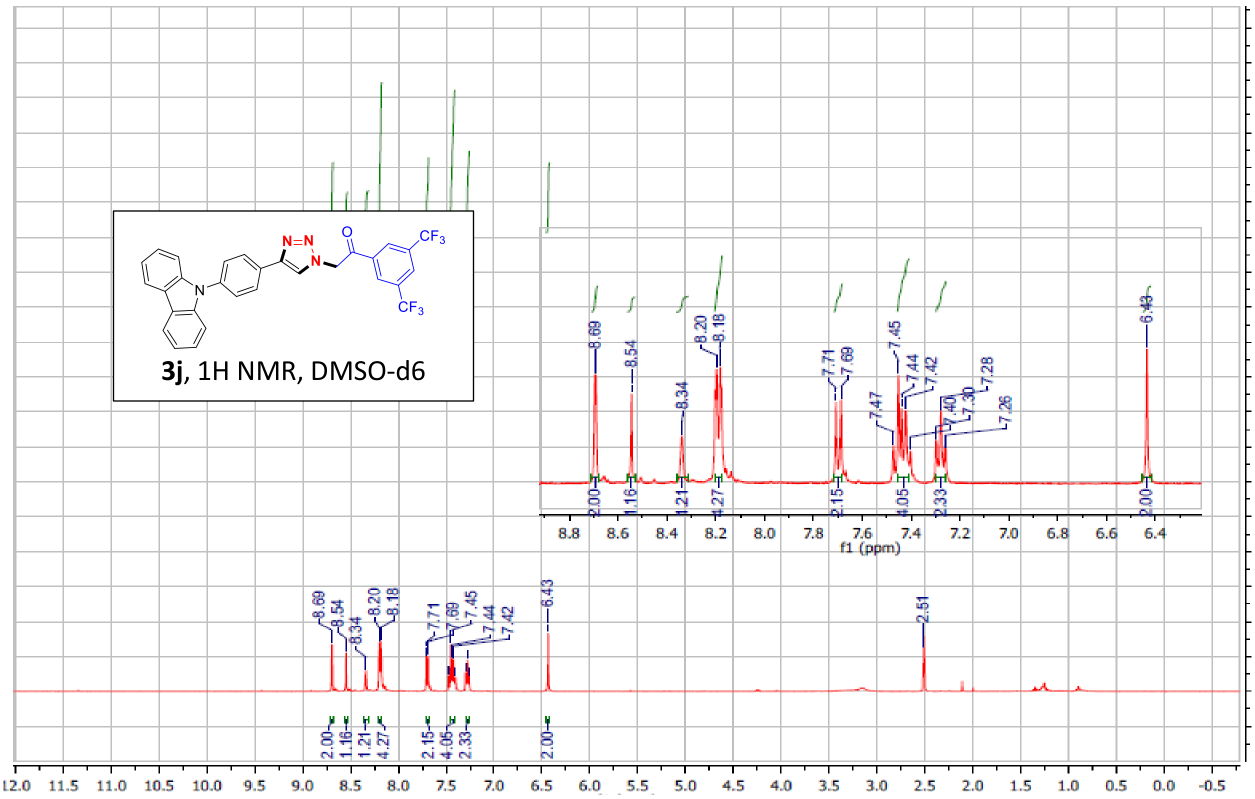
N=N
O
CF3
N
CF3
3j, 1H NMR, DMSO-d6
8.69
8.54
8.34
8.20
8.18
7.71
7.69
7.47
7.45
7.44
7.42
7.40
7.30
7.28
7.26
6.43
2.51
2.00
1.16
1.21
4.27
2.15
4.05
2.33
2.00
f1 (ppm)

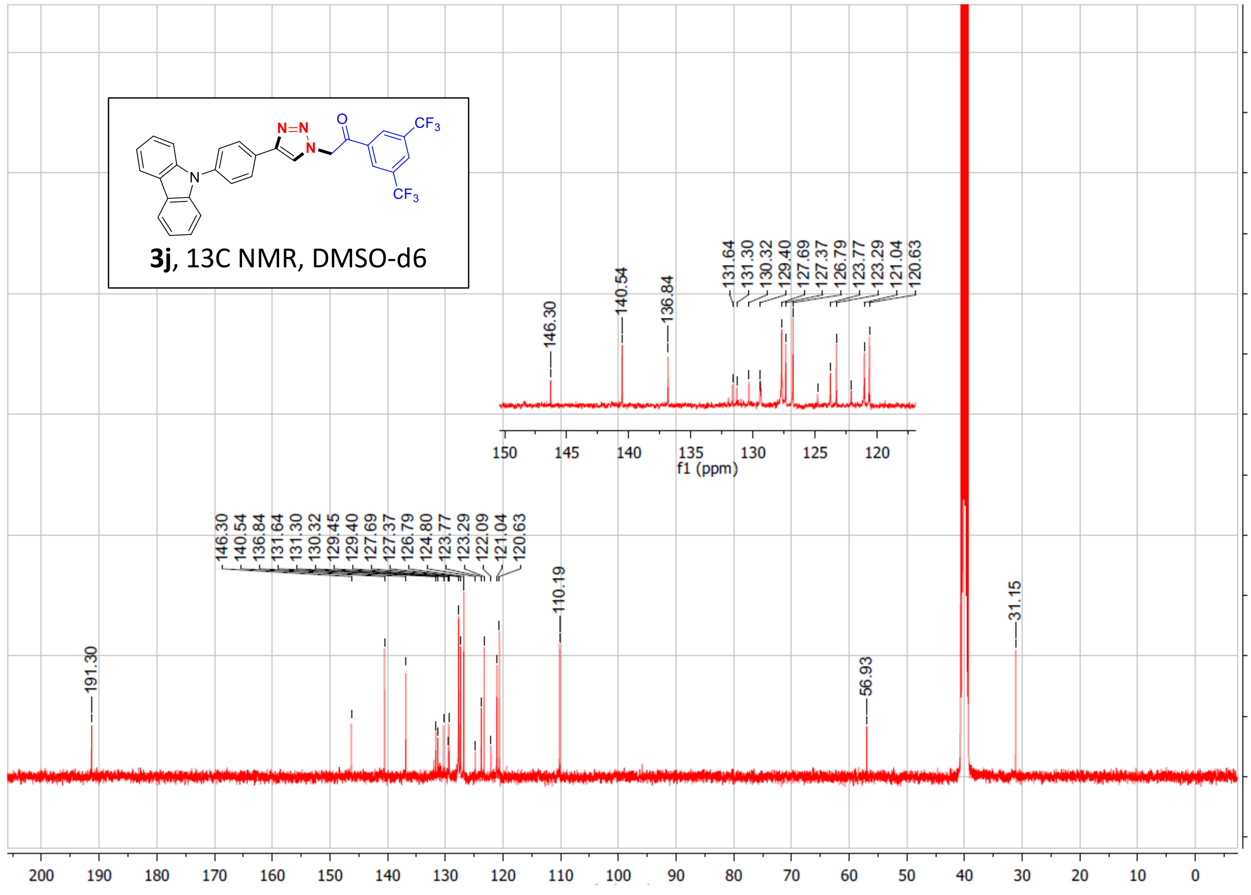
N=N
O
CF3
N
CF3
3j, 13C NMR, DMSO-d6
191.30
146.30
140.54
136.84
131.64
131.30
130.32
129.45
129.40
127.69
127.37
126.79
124.80
123.77
123.29
122.09
121.04
120.63
110.19
56.93
31.15
f1 (ppm)

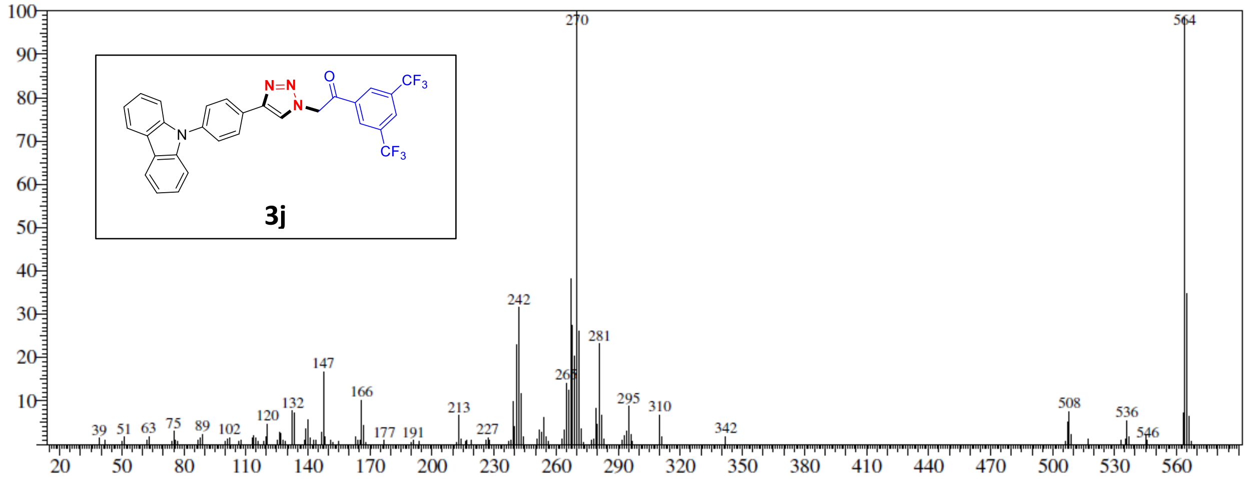

3j
270
564
242
281
147
166
508
536

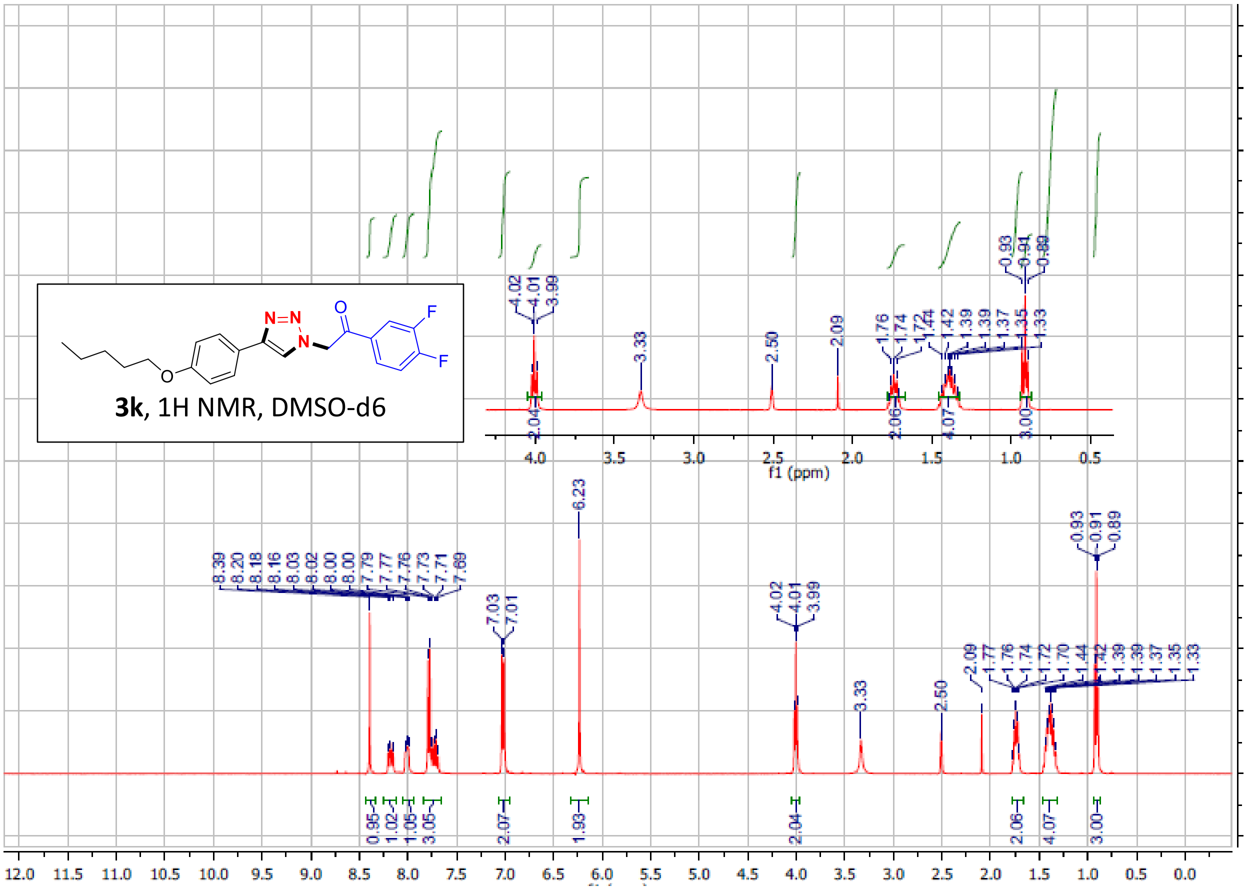

3k, 1H NMR, DMSO-d6
f1 (ppm)

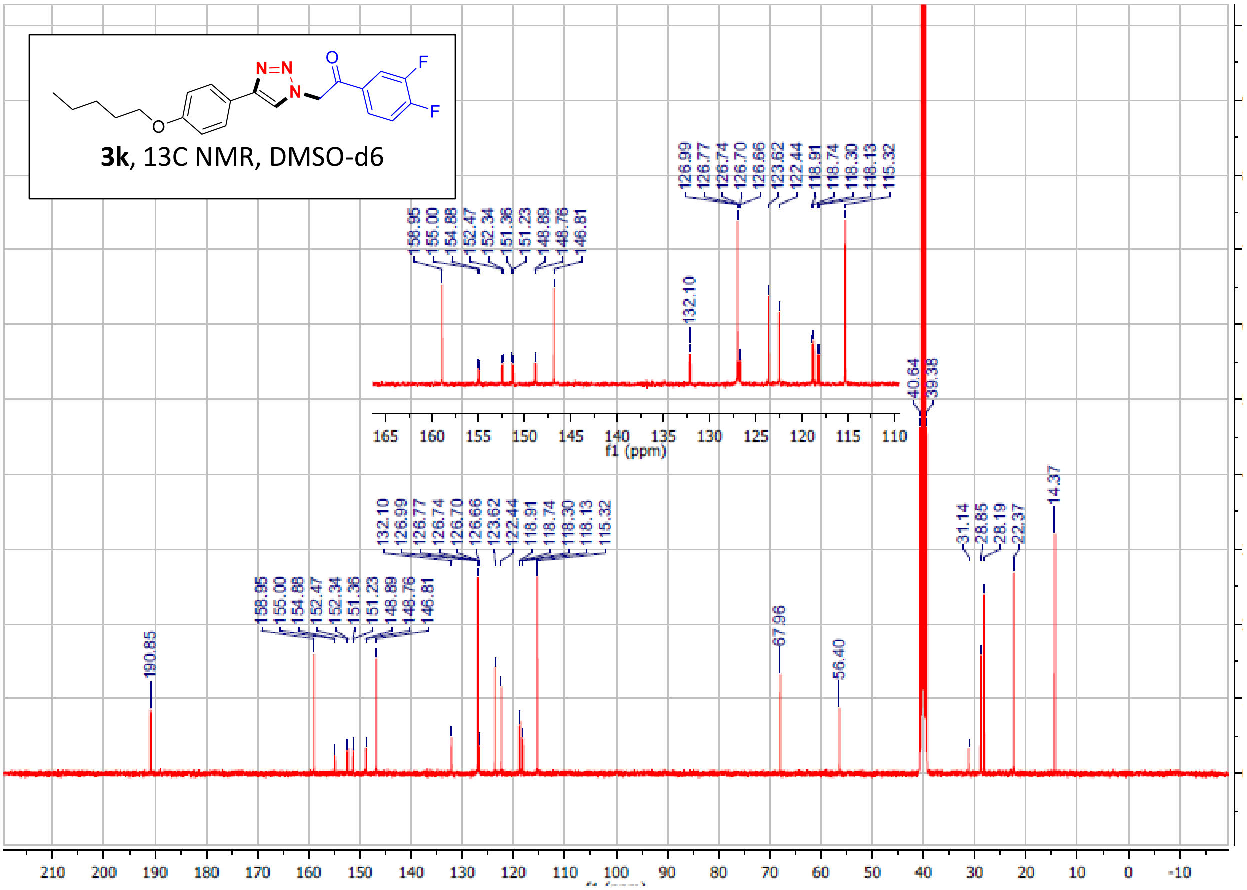
3k, 13C NMR, DMSO-d6
190.85
158.95
155.00
154.88
152.47
152.34
151.36
151.23
148.89
148.76
146.81
132.10
126.99
126.77
126.74
126.70
126.66
123.62
122.44
118.91
118.74
118.30
118.13
115.32
67.96
56.40
40.64
39.38
31.14
28.85
28.19
22.37
14.37
f1 (ppm)

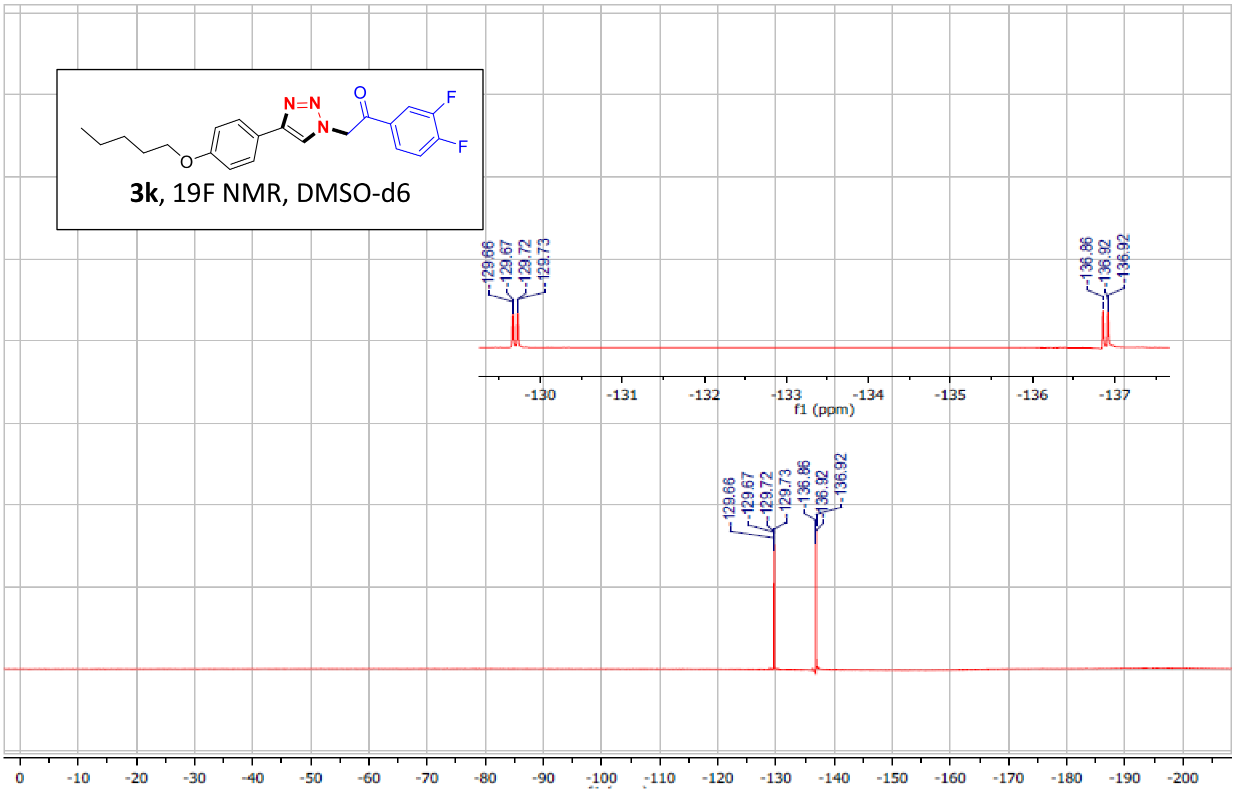
3k, 19F NMR, DMSO-d6
-129.66
-129.67
-129.72
-129.73
-136.86
-136.92
-136.92
f1 (ppm)

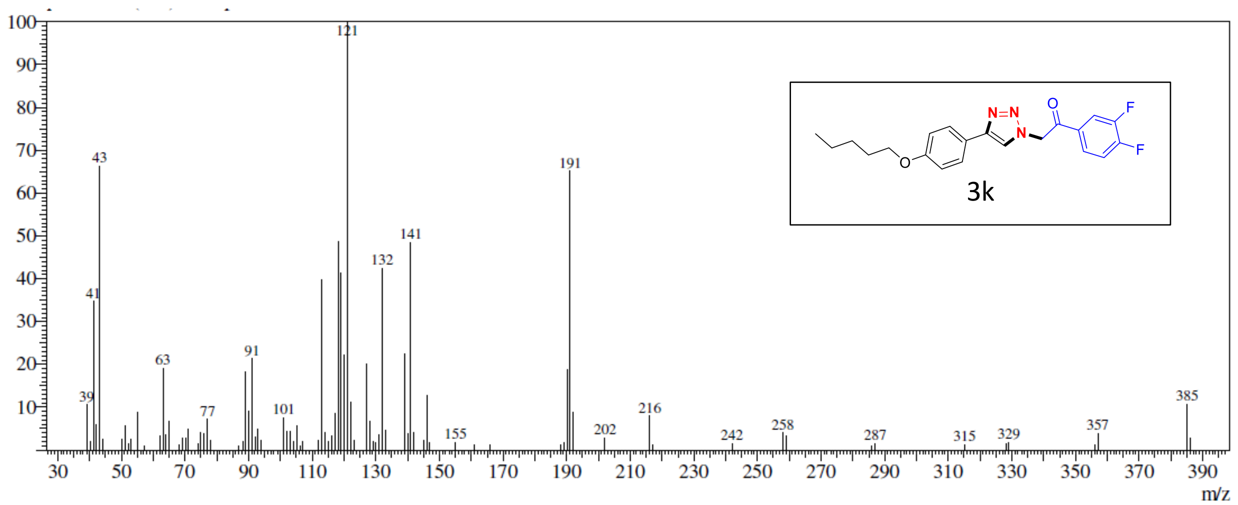
3k
m/z
121
191
43
141
132
41
63
91
77
101
39
155
202
216
242
258
287
315
329
357
385

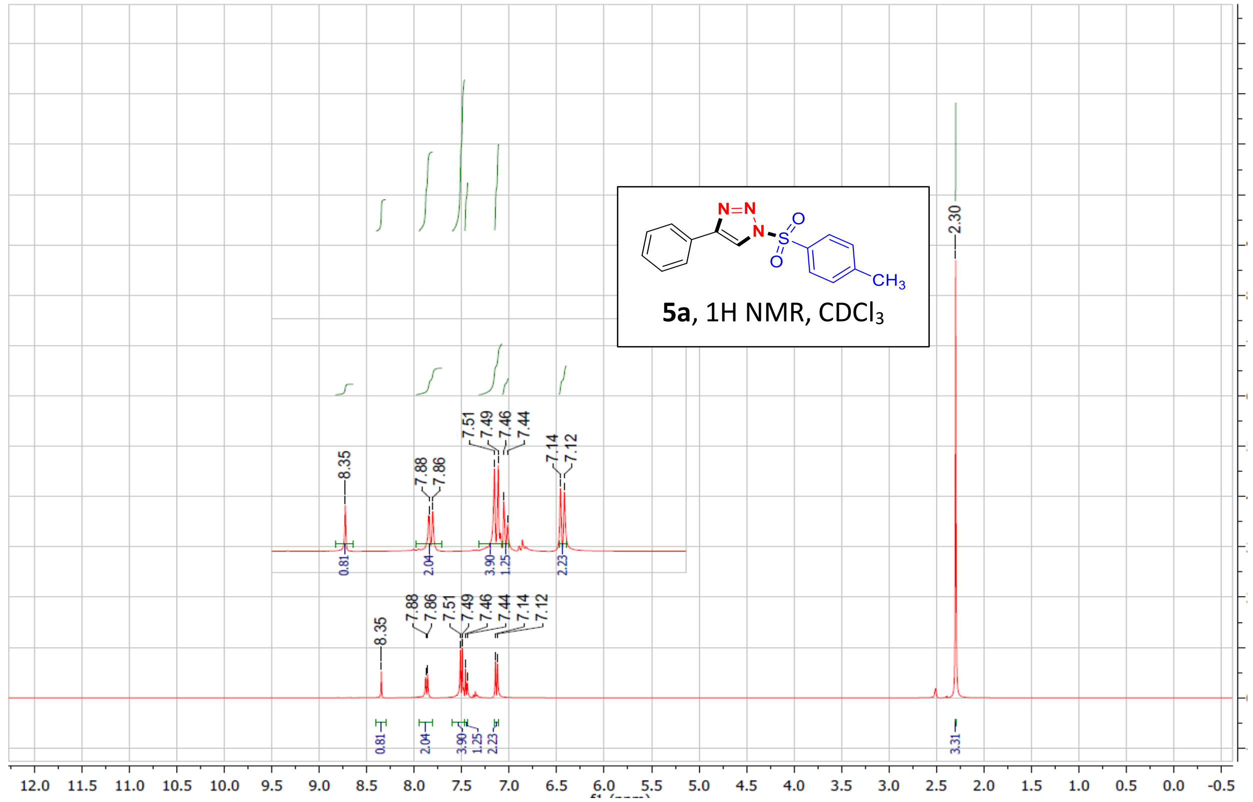
5a, 1H NMR, $CDCl_3$
8.35
7.88
7.86
7.51
7.49
7.46
7.44
7.14
7.12
2.30
0.81
2.04
3.90
1.25
2.23
3.31
f1 (ppm)

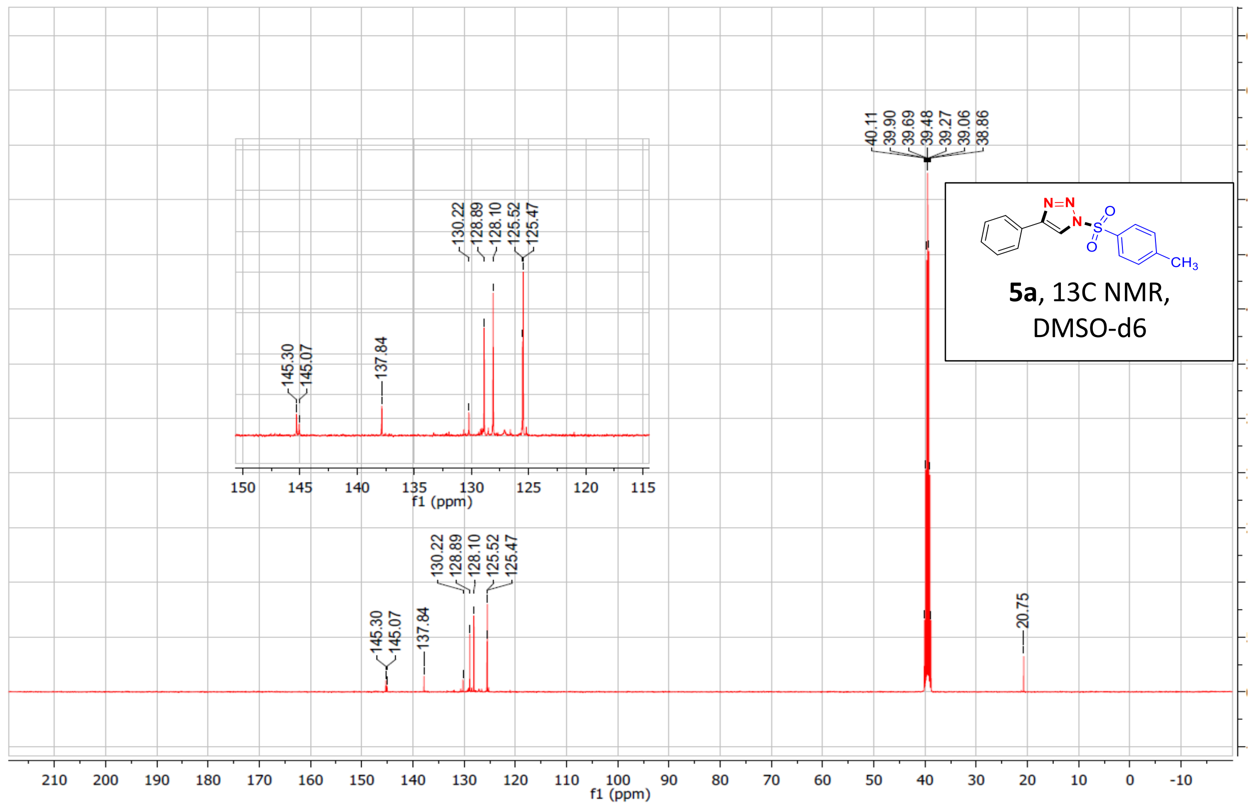
5a, 13C NMR,
DMSO-d6
f1 (ppm)
20.75
137.84
145.30
145.07
130.22
128.89
128.10
125.52
125.47
40.11
39.90
39.69
39.48
39.27
39.06
38.86

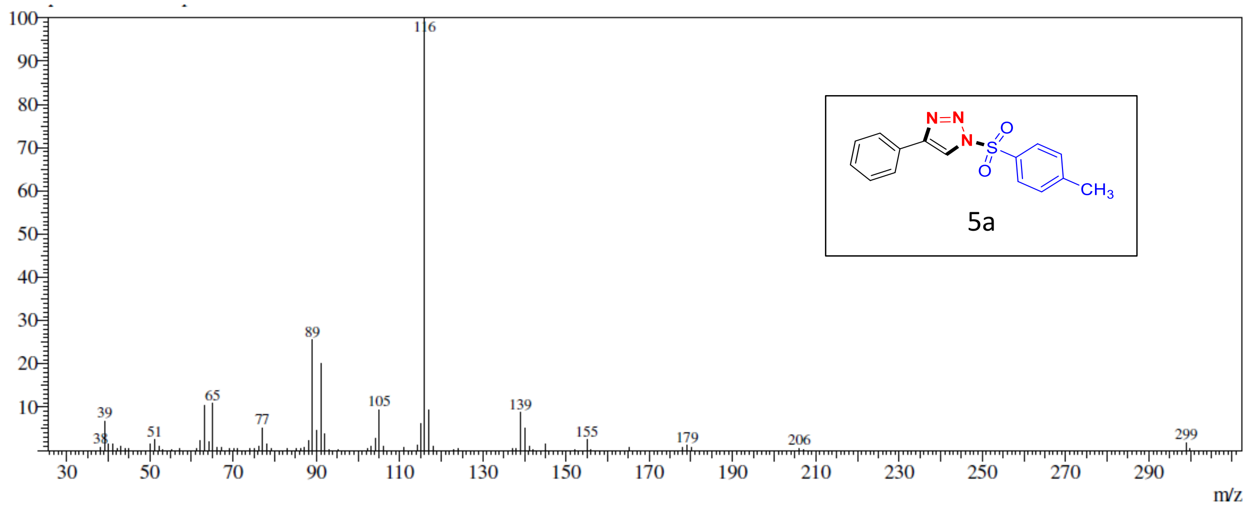
5a
m/z
116
89
105
139
65
77
39
51
155
179
206
299

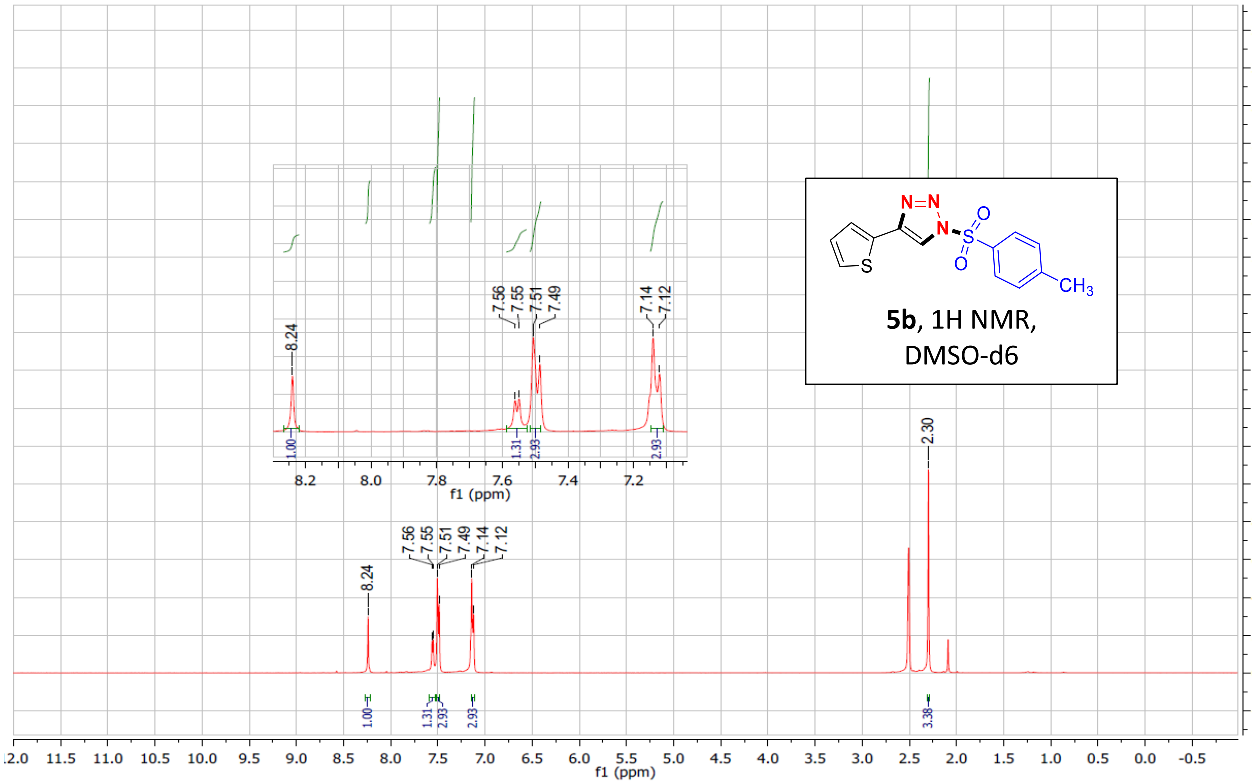
5b, 1H NMR,
DMSO-d6
N=N
N
S
O
O
CH3
8.24
7.56
7.55
7.51
7.49
7.14
7.12
2.30
1.00
1.31
2.93
2.93
3.38
f1 (ppm)

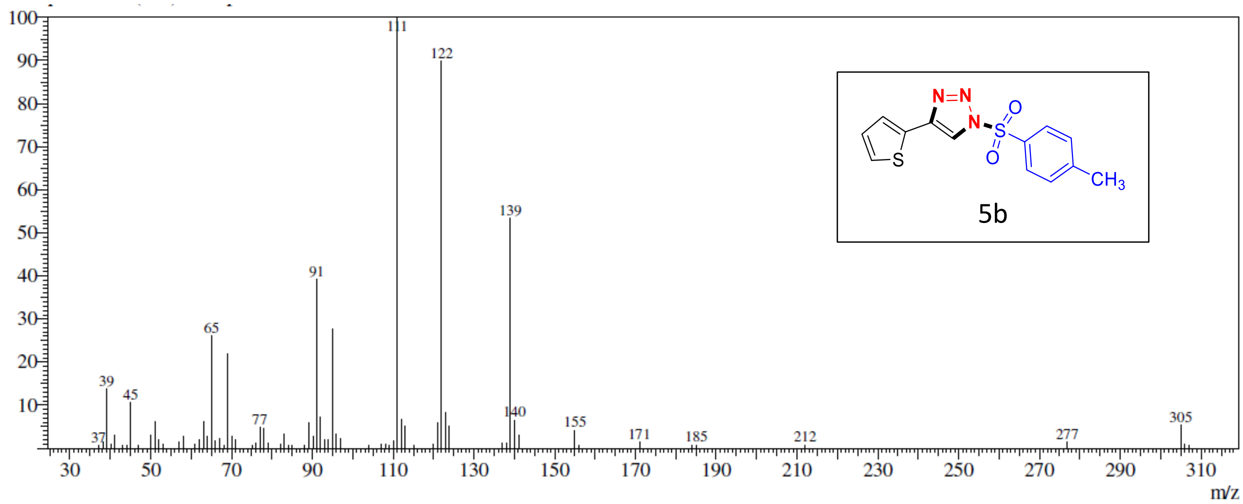
5b
N=N
N
S
O
O
CH3
111
122
139
91
65
39
45
37
77
140
155
171
185
212
277
305
m/z

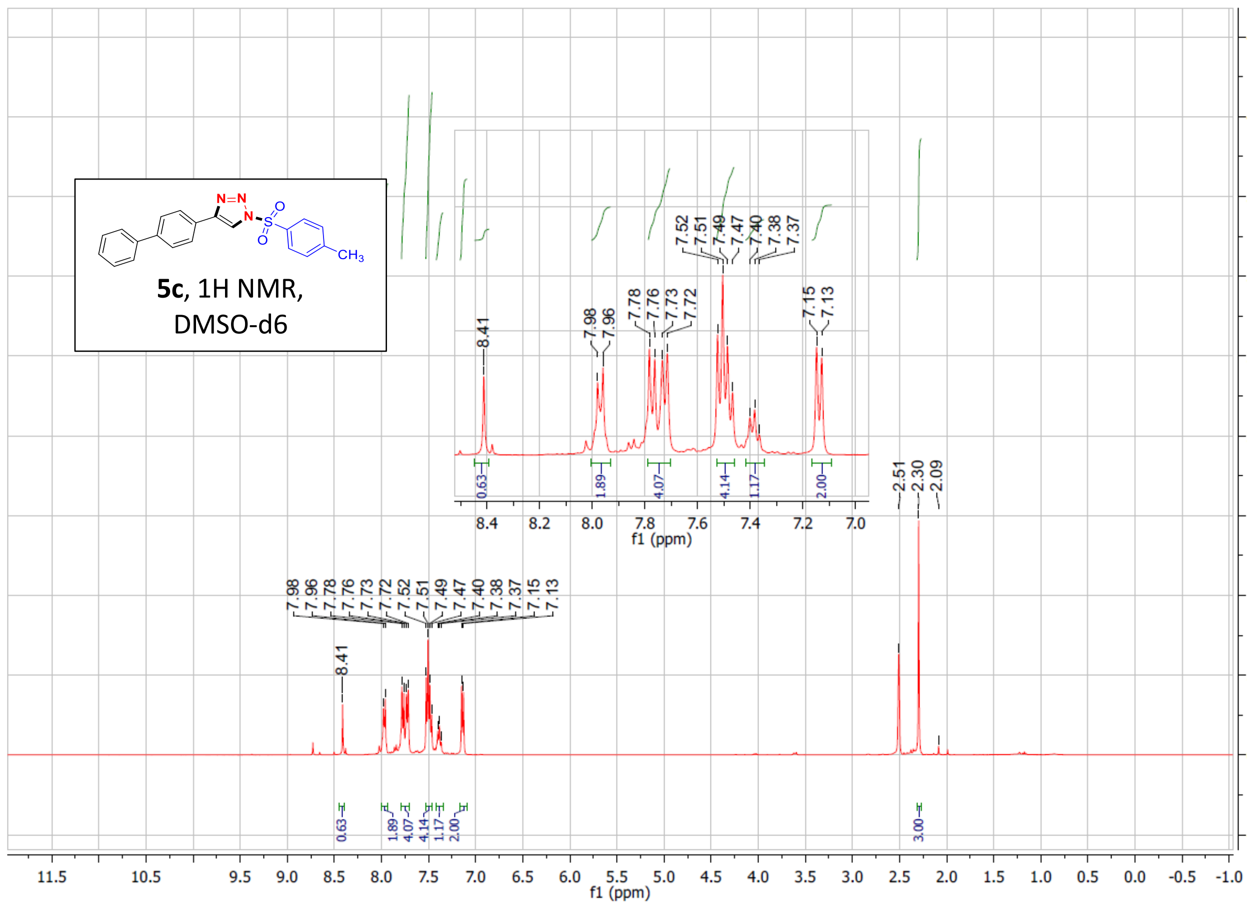
5c, 1H NMR, DMSO-d6
8.41
7.98 7.96 7.78 7.76 7.73 7.72 7.52 7.51 7.49 7.47 7.40 7.38 7.37 7.15 7.13
2.51 2.30 2.09
0.63 1.89 4.07 4.14 1.17 2.00 3.00
f1 (ppm)

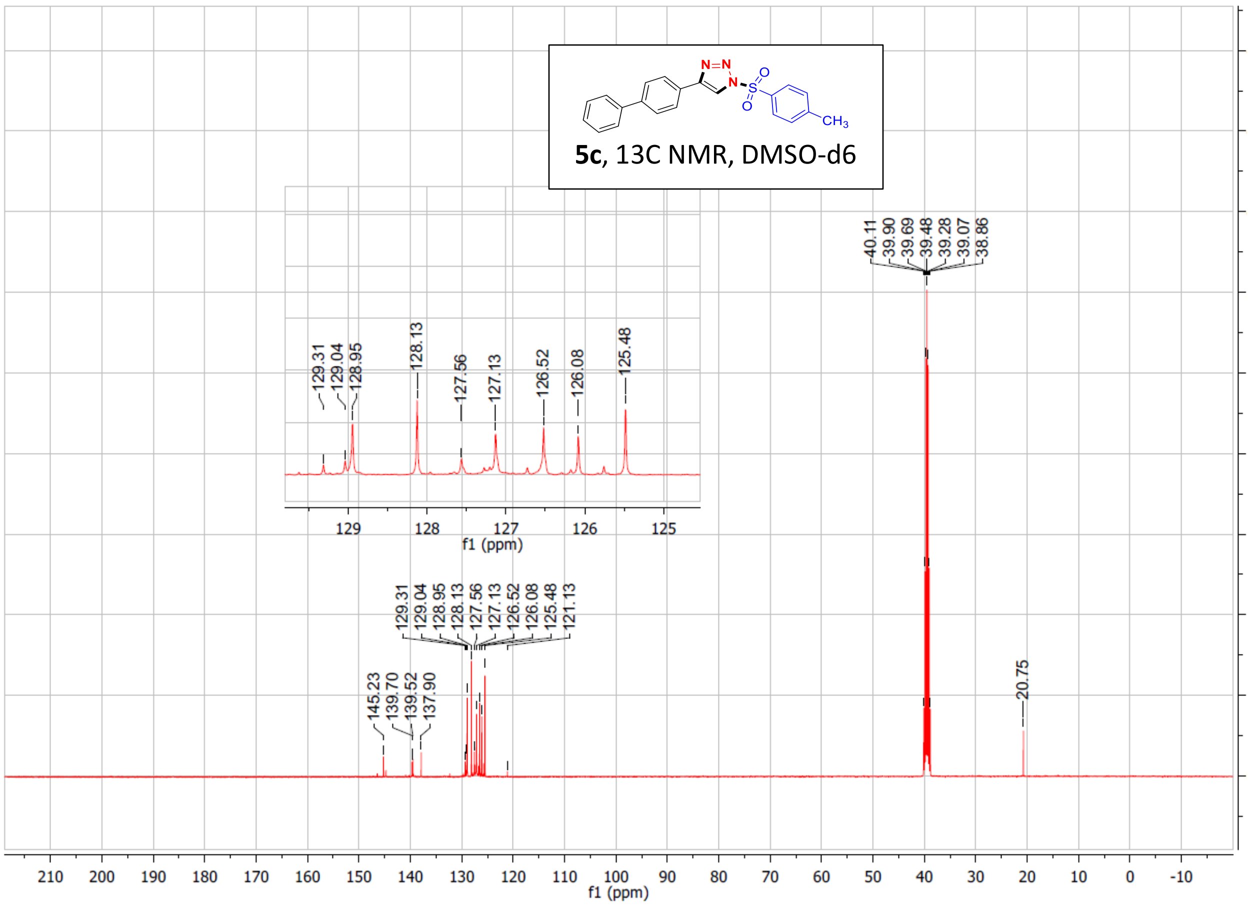
5c, 13C NMR, DMSO-d6
N=N
N
S
O
O
CH3
40.11
39.90
39.69
39.48
39.28
39.07
38.86
129.31
129.04
128.95
128.13
127.56
127.13
126.52
126.08
125.48
121.13
145.23
139.70
139.52
137.90
20.75
f1 (ppm)

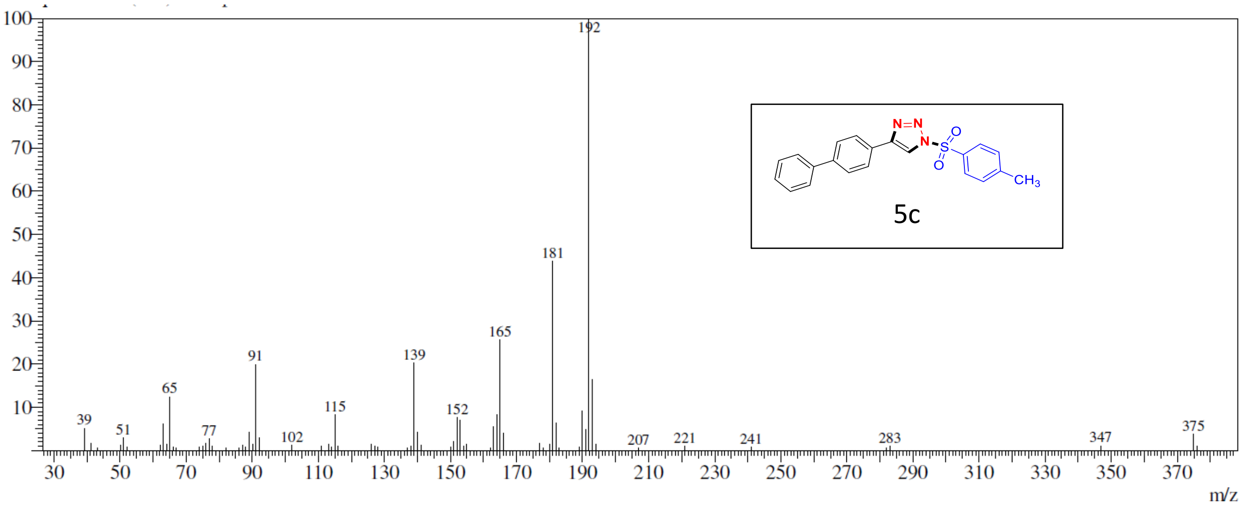
5c
N=N
N
S
O
O
CH3
192
181
165
152
139
115
102
91
77
65
51
39
207
221
241
283
347
375
m/z

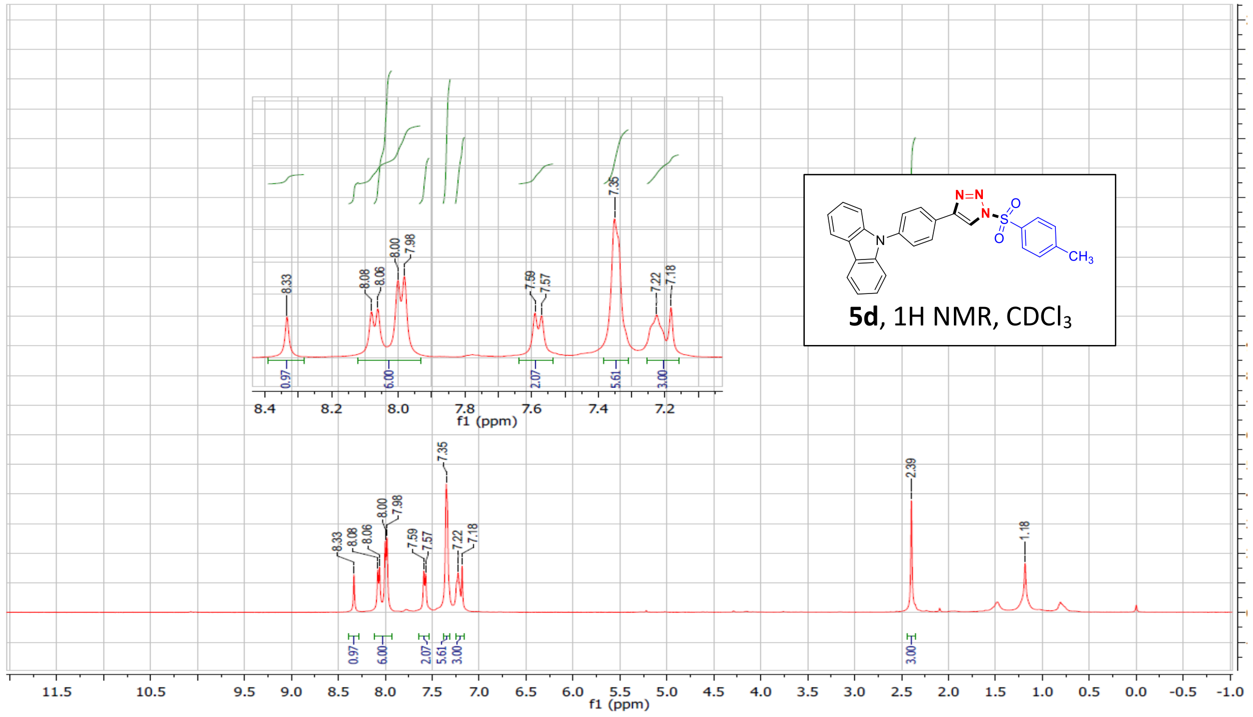
5d, 1H NMR, CDCl3
8.33
8.08
8.06
8.00
7.98
7.59
7.57
7.35
7.22
7.18
2.39
1.18
0.97
6.00
2.07
5.61
3.00
3.00
f1 (ppm)

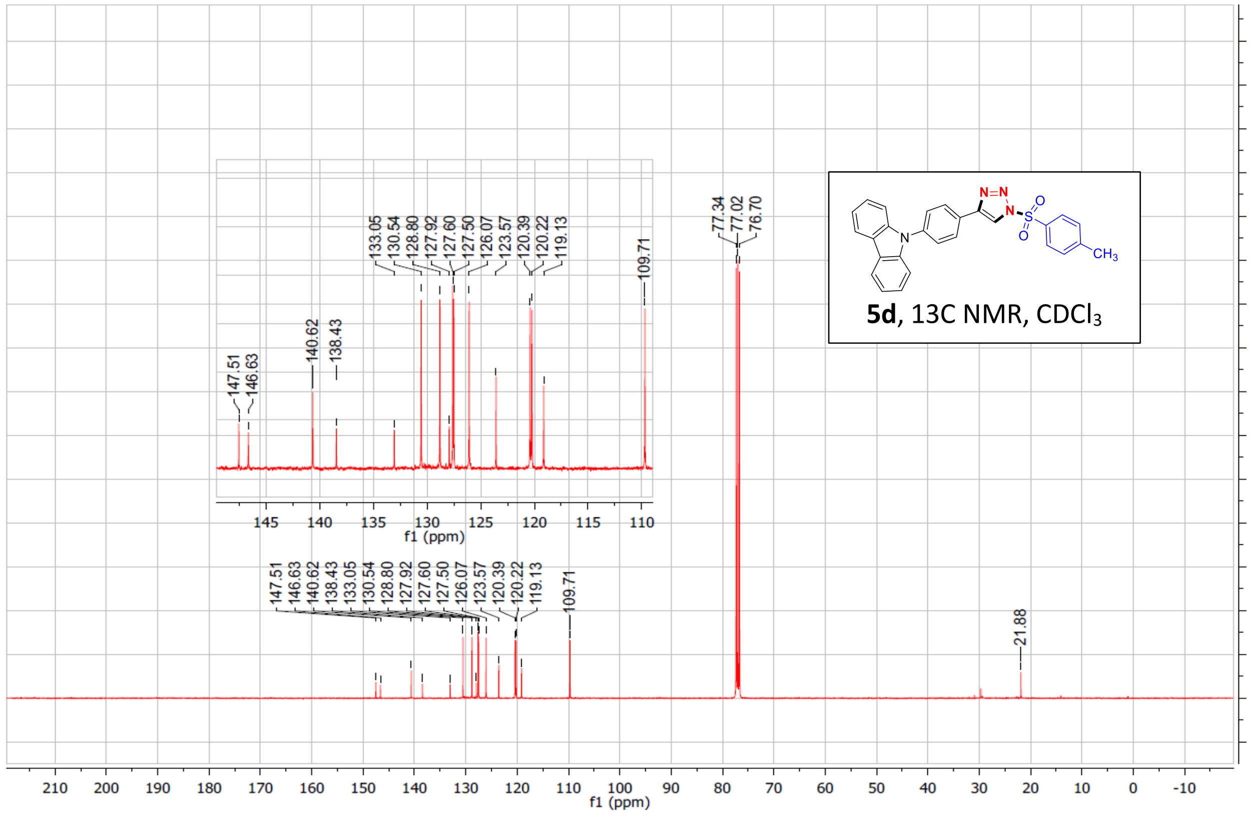
5d, 13C NMR, CDCl3
147.51
146.63
140.62
138.43
133.05
130.54
128.80
127.92
127.60
127.50
126.07
123.57
120.39
120.22
119.13
109.71
77.34
77.02
76.70
21.88
f1 (ppm)

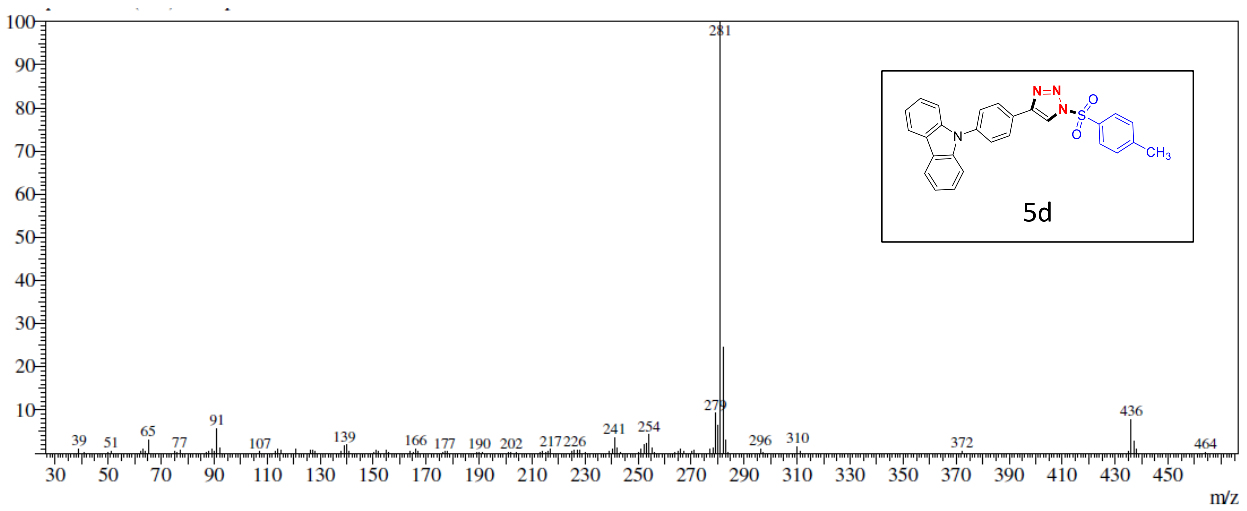
5d
m/z

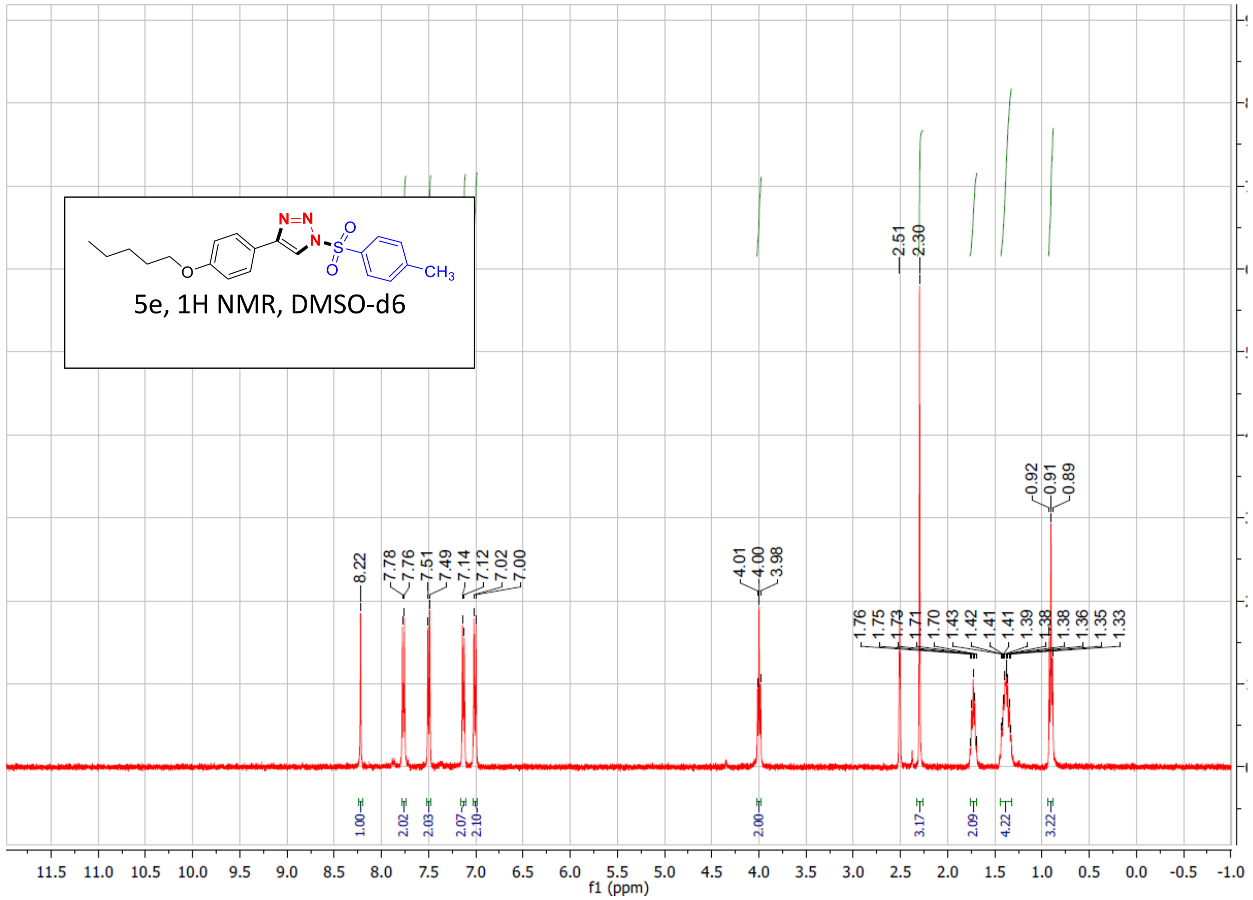
5e, 1H NMR, DMSO-d6
f1 (ppm)

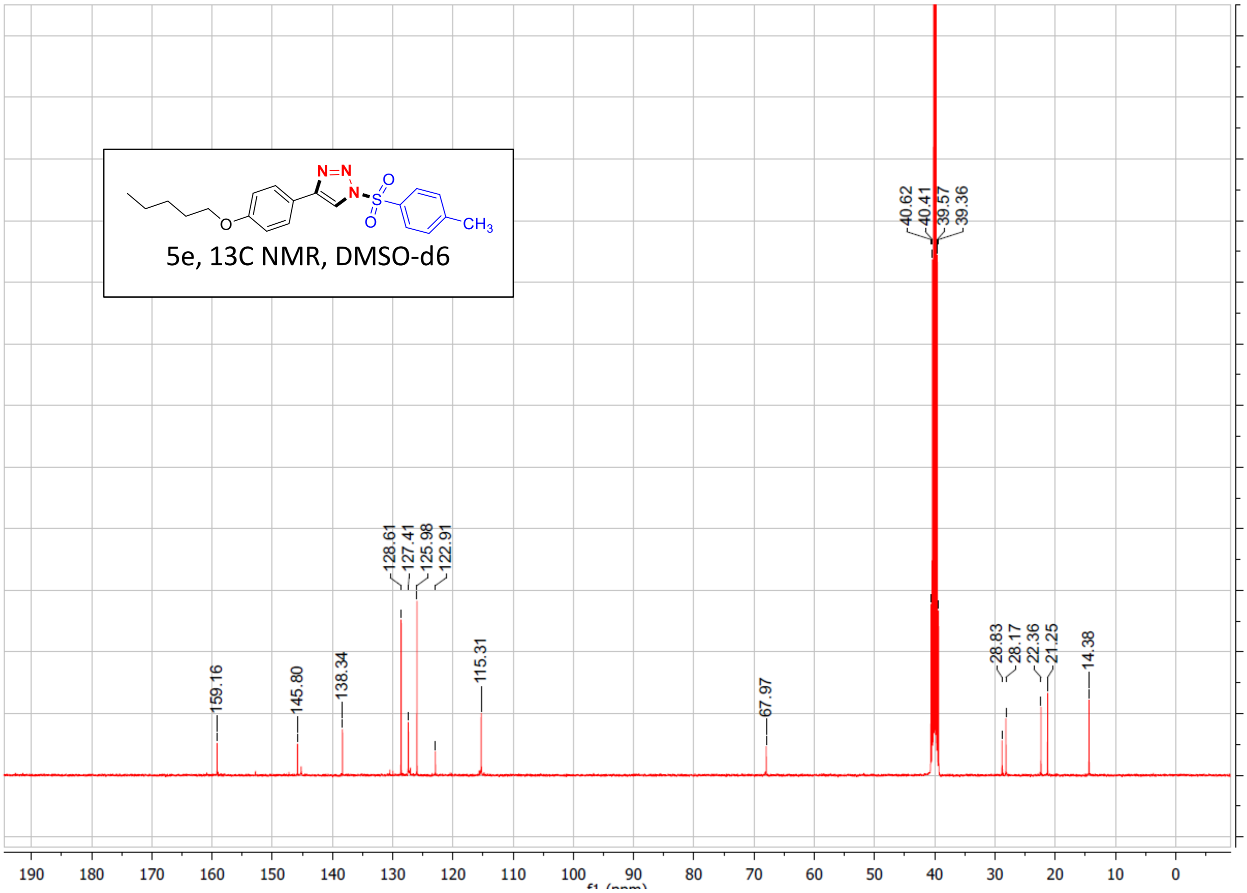
5e, 13C NMR, DMSO-d6
159.16
145.80
138.34
128.61
127.41
125.98
122.91
115.31
67.97
40.62
40.41
39.57
39.36
28.83
28.17
22.36
21.25
14.38
190 180 170 160 150 140 130 120 110 100 90 80 70 60 50 40 30 20 10 0

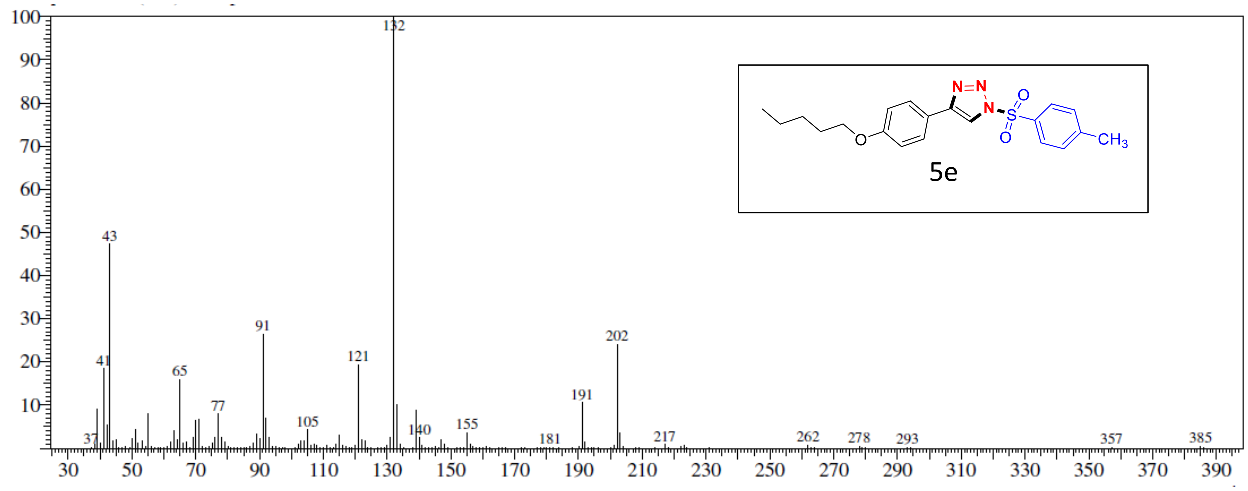
5e
132
43
41
65
91
77
105
121
140
155
181
191
202
217
262
278
293
357
385
37
100 90 80 70 60 50 40 30 20 10
30 50 70 90 110 130 150 170 190 210 230 250 270 290 310 330 350 370 390

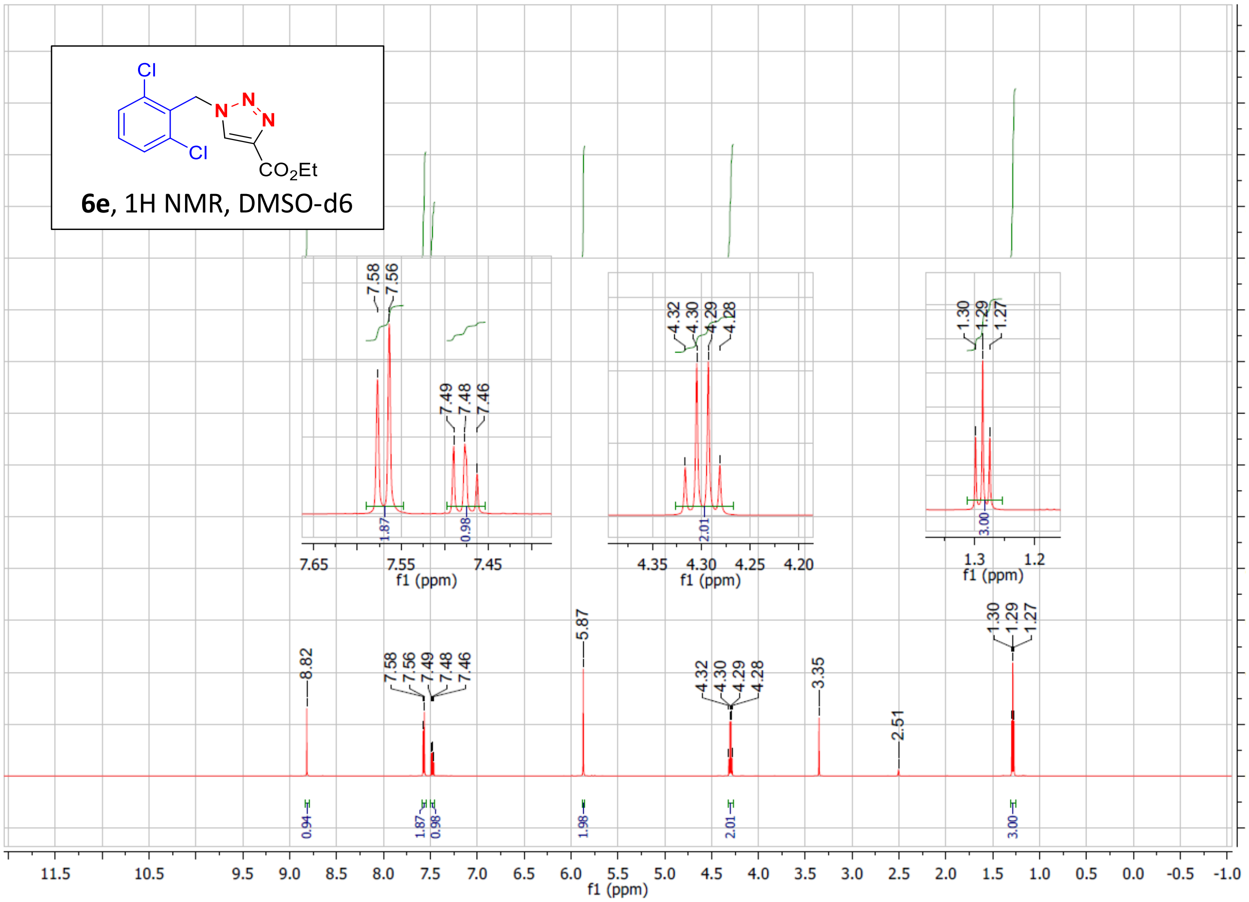
Cl
N
N
N
Cl
CO2Et
6e, 1H NMR, DMSO-d6
8.82
7.58
7.56
7.49
7.48
7.46
5.87
4.32
4.30
4.29
4.28
3.35
2.51
1.30
1.29
1.27
0.94
1.87
0.98
1.98
2.01
3.00
f1 (ppm)

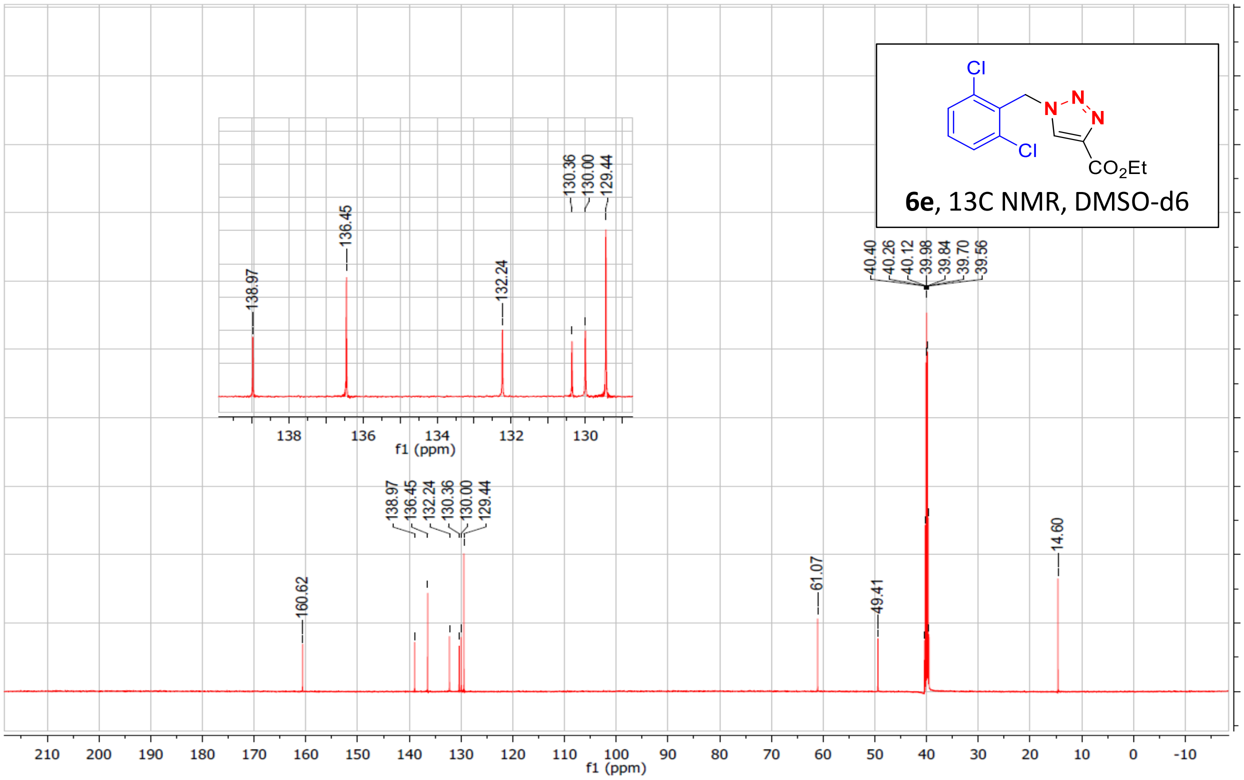
Cl
N
N
N
Cl
CO2Et
6e, 13C NMR, DMSO-d6
160.62
138.97
136.45
132.24
130.36
130.00
129.44
61.07
49.41
40.40
40.26
40.12
39.98
39.84
39.70
39.56
14.60
f1 (ppm)

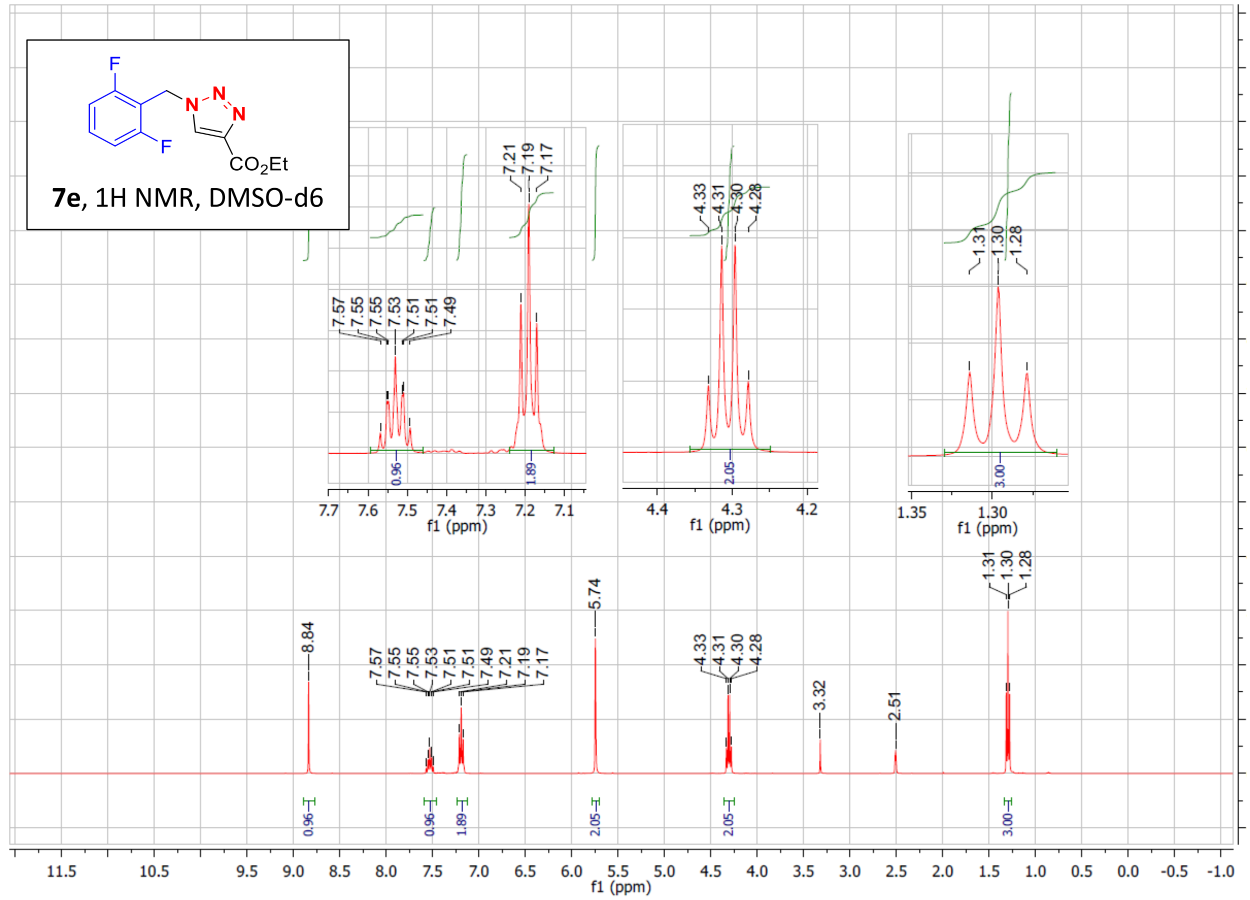
7e, 1H NMR, DMSO-d6
CO2Et
F
F
N
N
N
8.84
7.57
7.55
7.55
7.53
7.51
7.51
7.49
7.21
7.19
7.17
5.74
4.33
4.31
4.30
4.28
3.32
2.51
1.31
1.30
1.28
0.96
0.96
1.89
2.05
2.05
3.00
f1 (ppm)

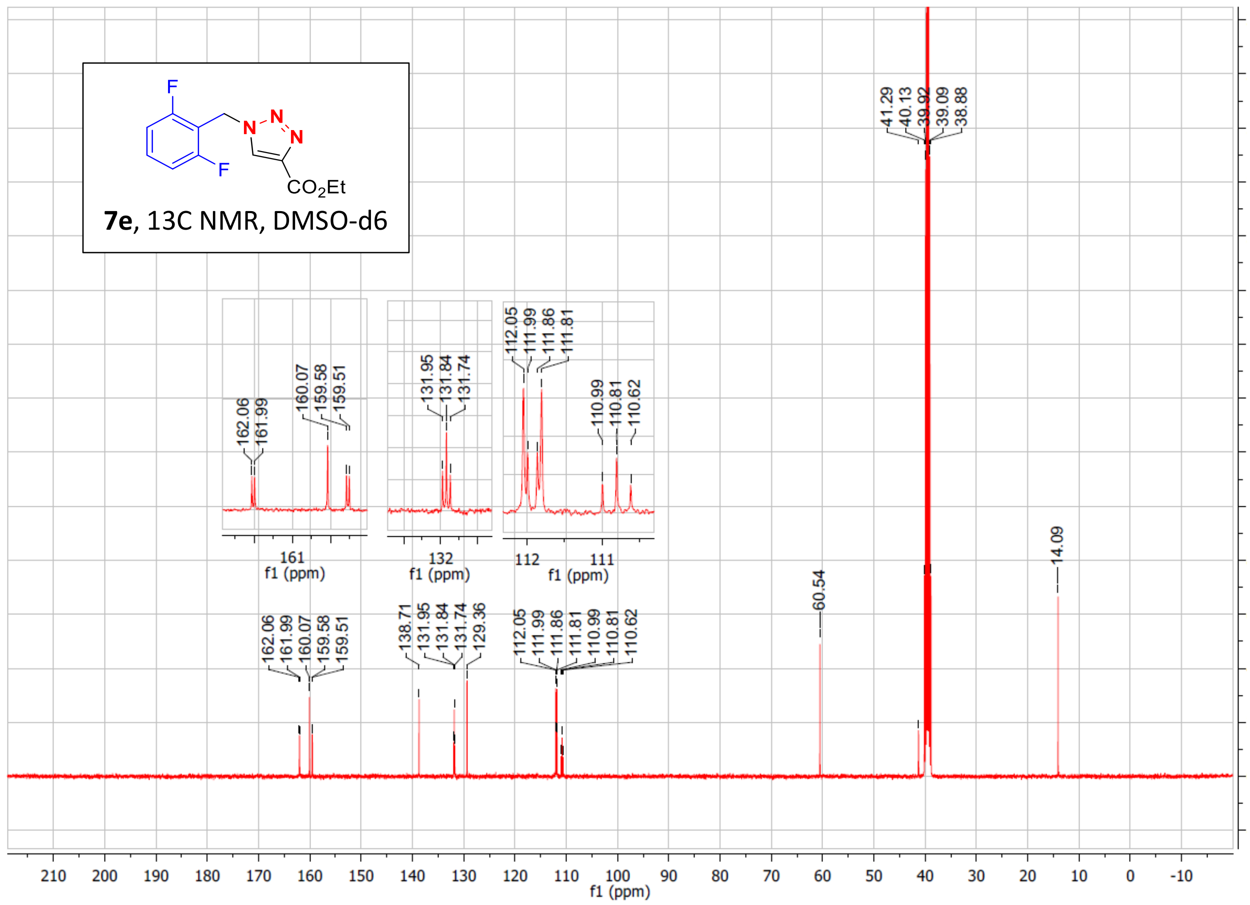

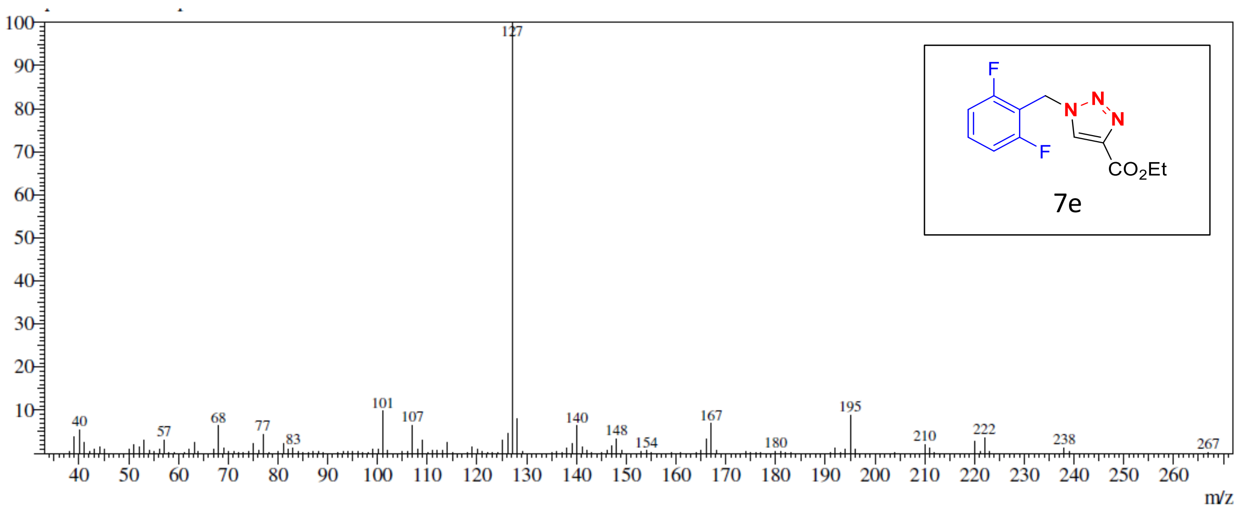

Figure S4: Spectral data (^{1}H, ^{13}C, GC-MS) for synthesized compounds

E. Single Crystal XRD data for the compound 3b.

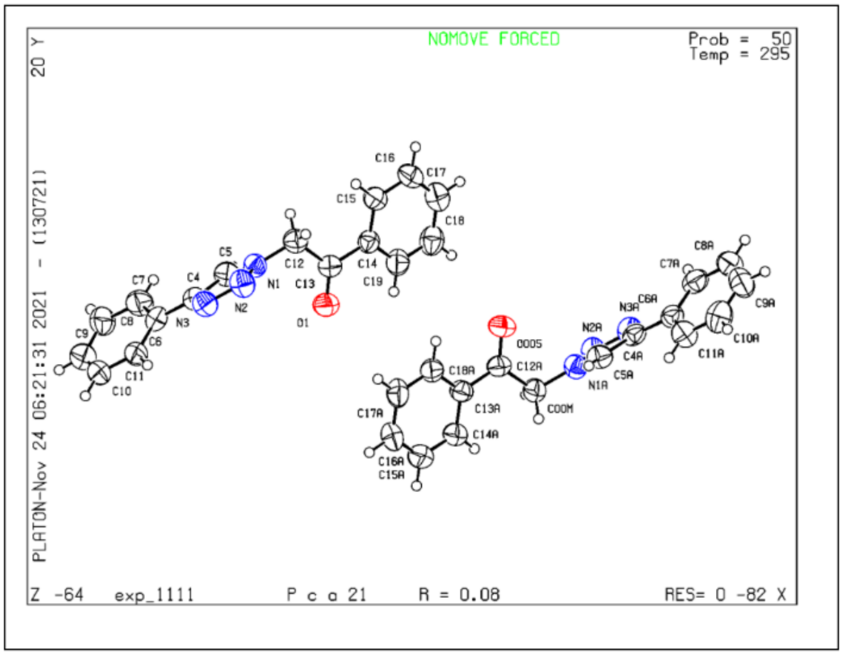

Figure S5: ORTEP diagram of compound **3b**.

Bond precision: C-C = 0.0131 A Wavelength=0.71073

Cell:	a=12.9892(12)	b=5.4807(7)	c=37.293(5)
	alpha=90	beta=90	gamma=90
Temperature:	295 K		

	Calculated	Reported
Volume	2654.9(5)	2654.9(5)
Space group	P c a 21	P c a 21
Hall group	P 2c -2ac	P 2c -2ac
Moiety formula	C16 H13 N3 O	C16 H13 N3 O
Sum formula	C16 H13 N3 O	C16 H13 N3 O
Mr	263.29	263.29
Dx,g cm-3	1.317	1.317
Z	8	8
Mu (mm-1)	0.085	0.085
F000	1104.0	1104.0
F000’	1104.41	
h,k,lmax	16,6,46	16,6,46
Nref	5430[2760]	4252
Tmin,Tmax	0.965,0.977	0.107,1.000
Tmin’	0.964	

Correction method= # Reported T Limits: Tmin=0.107 Tmax=1.000
AbsCorr = MULTI-SCAN

Data completeness= 1.54/0.78 Theta(max)= 26.369

R(reflections)= 0.0819(2393) wR2(reflections)= 0.2598(4252)

S = 1.023 Npar= 369

F. References

1. Curphey, T. J., PREPARATION OF *p*-TOLUENESULFONYL AZIDE. A CAUTIONARY NOTE, *Organic Preparations and Procedures International*, **1981**, *13*, 112-115.
